# EZH2 Inhibition Promotes Tumor Immunogenicity in Lung Squamous Cell Carcinomas

**DOI:** 10.1158/2767-9764.CRC-23-0399

**Published:** 2024-02-13

**Authors:** Tanner J. DuCote, Xiulong Song, Kassandra J. Naughton, Fan Chen, Daniel R. Plaugher, Avery R. Childress, Abigail R. Gellert, Erika M. Skaggs, Xufeng Qu, Jinze Liu, Jinpeng Liu, Fei Li, Kwok-Kin Wong, Christine F. Brainson

**Affiliations:** 1 Department of Toxicology and Cancer Biology, University of Kentucky, Lexington, Kentucky.; 2 Department of Biostatistics, Virginia Commonwealth University, Richmond, Virginia.; 3 Massey Cancer Center, Virginia Commonwealth University, Richmond, Virginia.; 4 Department of Cancer Biostatistics, University of Kentucky, Lexington, Kentucky.; 5 Laura and Isaac Perlmutter Cancer Center, NYU Langone Health, New York University, New York, New York.; 6 Markey Cancer Center, University of Kentucky, Lexington, Kentucky.

## Abstract

Two important factors that contribute to resistance to immune checkpoint inhibitors (ICI) are an immune-suppressive microenvironment and limited antigen presentation by tumor cells. In this study, we examine whether inhibition of the methyltransferase enhancer of zeste homolog 2 (EZH2) can increase ICI response in lung squamous cell carcinomas (LSCC). Our *in vitro* experiments using two-dimensional human cancer cell lines as well as three-dimensional murine and patient-derived organoids treated with two inhibitors of the EZH2 plus IFNγ showed that EZH2 inhibition leads to expression of both MHC class I and II (MHCI/II) expression at both the mRNA and protein levels. Chromatin immunoprecipitation sequencing confirmed loss of EZH2-mediated histone marks and gain of activating histone marks at key loci. Furthermore, we demonstrate strong tumor control in models of both autochthonous and syngeneic LSCC treated with anti-PD1 immunotherapy with EZH2 inhibition. Single-cell RNA sequencing and immune cell profiling demonstrated phenotypic changes toward more tumor suppressive phenotypes in EZH2 inhibitor–treated tumors. These results indicate that EZH2 inhibitors could increase ICI responses in patients undergoing treatment for LSCC.

**Significance:**

The data described here show that inhibition of the epigenetic enzyme EZH2 allows derepression of multiple immunogenicity factors in LSCC, and that EZH2 inhibition alters myeloid cells *in vivo*. These data support clinical translation of this combination therapy for treatment of this deadly tumor type.

## Introduction

Lung squamous cell carcinoma (LSCC) is a common subtype of non–small cell lung cancer (NSCLC) that historically has limited therapeutic options (1–3). The FDA recently approved first-line PD1/PD-L1 targeting immunotherapy for patients with LSCC (4). This therapy blocks the immune-evasion PD1/PD-L1 interaction, allowing for tumor-reactive T cells to expand and destroy the tumor. However, durable responses are seen in only approximately 20% of patients with advanced-stage LSCC (5). Efforts to increase the response rates in individuals have focused on combination therapies. Enhancer of zeste homolog 2 (EZH2) is a histone methyltransferase that catalyzes histone H3 lysine 27 trimethylation (H3K27me3), a mark associated with gene silencing (6). The FDA-approved EZH2 inhibitor tazemetostat (7, 8), as well as tool compounds including GSK126 (9), are specific EZH2 inhibitors that serve to decrease H3K27me3, derepress genes, and may lead to improved immunotherapy responses though several mechanisms.

To study LSCC in immunocompetent hosts, several autochthonous genetically engineered mouse models have been established. One such model was generated through biallelic deletion of the tumor suppressors *Pten* and *Lkb1* (a.k.a *Stk11*; ref. 10). Tumors from these mice were shown to have transcriptional similarity to human LSCC, had high expression of PD-L1 on the tumor-propagating cells, and had predominant populations of tumor-associated neutrophils (10). It is widely believed that tumor-associated neutrophils, in particular neutrophils that are sometimes described as granulocytic myeloid-derived suppressor cell, can promote tumor growth by creating a lymphocyte-suppressive microenvironment (11). Mechanisms through which neutrophils suppress T cells include high expression of arginase and reactive oxygen species (11). However, some neutrophils are thought to be tumor eliminating, and can create a prolymphocyte environment through production of TNFα and CXCL10, and antigen presentation (11). The neutrophil to lymphocyte ratio appears to strongly predict response to immunotherapy, suggesting that in most NSCLCs, neutrophils are T-cell suppressive (12).

One essential mechanism for T-cell activation is costimulation of antigens presented by MHCI and MHCII. Many tumor cells have evolved to repress antigen presentation machinery to evade the immune system surveillance (13). It has been reported that patient tumors with high expression of MHCII demonstrate greater response to anti-PD-1 checkpoint inhibitors in melanoma (14). In other cancer types, it has been demonstrated that both MHCI and MHCII can be regulated by the chromatin-modifying enzyme EZH2 (13, 15–18).

Here, we utilized several murine and human models of LSCC to understand whether and how EZH2 inhibition will boost immunotherapy responses. We found that inhibition of EZH2 catalytic activity with either GSK126 or EPZ6438 in the presence of IFNγ was able to derepress numerous genes encoding antigen presentation constituents and the pro-T cell cytokines CXCL9/10/11 in both human and murine tumoroids. Chromatin immunoprecipitation sequencing (ChIP-seq) in human patient-derived tumoroids (PDT) further delineated the patterns of epigenetic changes in response to EZH2 inhibition and IFNγ treatment. In both autochthonous and syngeneic grafts, EZH2 inhibition alone or with immunotherapy led to excellent tumor control. Single-cell RNA sequencing (scRNA-seq) and flow cytometry showed changes consistent with more immunogenic tumor cells and a more pro-T cell tumor microenvironment. Together, these data strongly support the addition of EZH2 inhibition to immunotherapy regimens that have now become first-line treatment for many patients with LSCC.

## Materials and Methods

### Animal Work

The studies described in this article employ genetically engineered mouse models of squamous lung cancer through the biallelic deletion of the genes *Lkb1 and Pten.* Both alleles are flanked by loxP sites and are deleted by Cre-recombinase administration. Experimental animals are inoculated with 2.9–5 × 10^7^ PFU adeno-CMV-Cre (University of Iowa, Iowa City, IA) through intranasal instillation and are monitored for tumor burden via MRI starting around 40 weeks after infection. Once tumors were detected, animals were randomized and placed onto one of four treatment arms. GSK126 was formulated by adding solid GSK126 (MedChem Express/Xcess Bio) to Captisol, chopped very finely, then added to sterile saline pH 4.6, and the solution was sonicated. Anti-PD1 clone RMP1–14 (BioXCell #BP0146, RRID:AB_10949053 or #BE0146, RRID:AB_10949053) and IgG2a isotype control (BioXCell #BP0089m, RRID:AB_1107769) were diluted with InVivoPure pH 7.0 dilution buffer (BioXCell #IP0070) or InVivoPure pH 6.5 dilution buffer (BioXCell # IP0065) to a concentration of 1.25 µg/µL. Animals were administered GSK126 at 300 mg/kg i.p. twice per week, 5–7 mg/kg anti-mouse PD1 intraperitoneal immunotherapy three times per week, or a combination of both GSK126 and anti-PD1. Placebos used for treatment arms were Captisol in sterile saline and rat IgG2a isotype antibody. Treatment regimen was 4–6 weeks and animals were monitored for tumor burden every 2 weeks via MRI. Both male and female mice were used and no sex differences were observed. For injection of tumoroids, dissociated cells were counted and resuspended in 1:1 v:v saline and Matrigel solution and approximately 250,000 cells were injected into each flank of the parental mouse strain, or NSG mice (JAX strain #005557 NOD.Cg-Prkdcscid Il2rgtm1Wjl/SzJ). The tumoroid culture used was from female, and both male and female recipient mice were used and no sex differences were observed. Tumors were measured by caliper and at 6–7 weeks postinjection, treatment began on mice with measurable tumors. To prepare formulation of EPZ6438 (MedChem Express #HY-13803/CS-1758), EPZ6438 was added to a solution of 0.1% Tween 80 and 0.5% sodium carboxymethylcelluose and the solution was sonicated. EPZ6438 was administered 250 mg/kg by gavage twice daily for 14 days. All experiments were approved by the Dana-Farber Cancer Institute or University of Kentucky Institutional Animal Care and Use Committees.

### Cell Lines

Cell lines were maintained according to University of Kentucky biosafety guidelines. All human cell lines were cultured in RPMI1640 (Gibco, #11875-093), supplemented with 8% FBS (VWR), penicillin/streptomycin (Gibco, #15140-122), and 4 mmol/L GlutaMAX (Gibco, #35050-061) at 37°C and 5% CO_2_. All cell lines were tested regularly for *Mycoplasma* with MycoAlert PLUS Mycoplasma Detection Kit (Lonza) last performed on August 2, 2022 and prophylactic treatment with Plasmocin (InvivoGen, #ant-mpt) was used routinely. Cell lines A549 (RRID: CVCL_0023), H520 (RRID: CVCL_1566), HCC15 (RRID: CVCL_2057), HCC95 (RRID: CVCL_5137), were verified by short tandem repeat analysis with CellCheck9 by IDEXX laboratories before beginning of experiments and used within 10 passages of authentication. A549 and H520 were originally from ATCC and HCC15 and HCC95 were from Dr. Meyerson's laboratory at Dana-Farber Cancer Institute.

### Flow Cytometry Analysis and Sorting

For all two-dimensional (2D) flow cytometry experiments, cells were trypsinized from culture plates and incubated with antibodies at 1:100 dilution in PBS + 10% FBS (PF10) for 15 minutes at room temperature. The cells were then resuspended in 300 µL of PF10 + 4′,6-diamidino-2-phenylindole (DAPI; 1:250). Antibodies for human cell analysis: EpCAM-FITC (BD Biosciences, #347197, RRID:AB_400261), PD-L1-PE (eBioscience, #12-5983-42, RRID:AB_11042286), CD49f- Alexa Fluor 647 (BD Biosciences, #562473, RRID:AB_11153684), NGFR-PECy7 (BioLegend, #345110, RRID:AB_11203542), CD49f-FITC (Invitrogen, #11-0495-82, RRID:AB_11150059), HLA-DR-APCCy7 (BioLegend, #307618, RRID:AB_493586), and HLA-A,B,C-APC (BioLegend, #311410, RRID:AB_314879). Antibodies used for mouse studies: I-A/I-E-PerCP-Cy5.5 (BioLegend #107626, RRID:AB_2191071), H-2K^d^/H-2D^d^-Alexa Fluor 647 (BioLegend #114712, RRID:AB_493063), NGFR (Cell Signaling #8238S, RRID:AB_10839265), anti-Rabbit-FITC (Invitrogen #F2765, RRID:AB_2536525), PD-L1-PE (BioLegend #124308, RRID:AB_2073556), Sca1-APCCy7 (BioLegend #108126, RRID:AB_10645327), EpCAM-PECy7 (BioLegend #118216, RRID:AB_1236471), CD49f-FITC (Invitrogen #11-0495-82, RRID:AB_11150059). All antibodies were bound at room temperature for 10 minutes at a dilution of 1:100, with the exception of Sca1 that was used at 1:50. Syngeneic grafts were dissected, minced, dissociated with collagenase/dispase (SIGMA 10269638001, 6 mg/mL) for 45 minutes at 37°C and filtered through 40 µm cell strainers. Antibodies used for staining were: anti-PD1-BV421 (BioLegend #135218, RRID:AB_2561447), anti-CD8-BV711 (BioLegend #100747, RRID:AB_11219594), anti-CD4-BV785 (BioLegend #100552, RRID:AB_2563053), anti-CD45-FITC (BioLegend #103108, RRID:AB_312973), anti-CD3-BB700 (BioLegend #100328, RRID:AB_893318), anti-Rat-IgG2A-PECF594 (BioLegend #405432, RRID:AB_2687101), Ly6G-BV786 (BioLegend #127645, RRID:AB_2566317), F4/80-PECF594 (BioLegend #123146, RRID:AB_2564133), CD11b-PECy7 (BioLegend # 101216, RRID:AB_312799) with the live-dead stain Zombie UV (BioLegend 423108). AnnexinV staining was performed with the BioLegend AnnexinV-FITC, 7AAD kit (#655942) on bone marrow samples (described below) according to the manufacturer's instructions. All analysis was done on BD LSRII or Symphony machines. FlowJo software was used to calculate percentages of cells and for many experiments, two different stains or experiments were used for each biological replicate and each considered as an n for the experiment as indicated in figure legends.

### Bone Marrow Histopaque Gradient, Cytospin, and Staining

Following euthanasia, mouse legs were removed at the acetabulum and placed into magnesium/calcium-free PBS (Cytiva, #SH30256.02). The bones were cleaned and flushed into a 1.5 mL tube using magnesium/calcium-free PBS. The remaining cells were pelleted via pulse centrifugation then were subject to red blood cell lysis in 250 µL of red blood cell lysis. Following red blood cell lysis, the cells were washed with 1 mL of calcium/magnesium-free PBS then pelleted with pulse spin centrifugation. Cells were then plated for apoptosis at 500,000 cells in 12-well plates in DMEM/F12 media (Thermo Fisher Scientific, 11330032) with an additional 4 mmol/L Glutamax (Thermo Fisher Scientific, 35050061), 5 µg/mL ITS (insulin/transferrin/selenium, SIGMA, I3146), and 8%–9% FBS (VWR, 97068-085). Remaining cells were resuspended in 1 mL of calcium/magnesium-free PBS. For Histopaque gradient, in a 5 mL flow cytometry tube, 1.5 mL of Histopaque 1119 (SIGMA #RNBK6705) was added at room temperature and 1.5 mL of Histopaque 1077 (SIGMA #RNBL3022) at room temperature was carefully pipette on top. Samples were then added on top of the histopaque layers slowly to persevere the interface between the layers. The tubes were then spun at 25°C at 1,000 × *g* for 25 minutes with no brake. Following centrifugation, the cloudy interface was collected into a 1.5 mL tube and washed with 1 mL of magnesium/calcium-free PBS. The cells were resuspended in 1 mL of magnesium/calcium-free PBS and 150 µL was taken to perform cytopsin. For cytopsin, 150 µL cell suspension was pipetted into funnel and slides were spun at 550 rpms for 1 minute. The areas where cells had adhered were then circled with a wax pen and 150 µL of paraformaldehyde or formalin was added for 15 minutes. The fixing agent was tapped off and then 150 µL of PBS with 0.1% Triton-X was added for 15 minutes. The slides were stained using hematoxylin for 1 minute. The slides then proceed from 70% ethanol for 1 minute, 95% ethanol for 1 minute, 100% ethanol for 3 minutes, xylene for 3 minutes, and finally xylene for 3 minutes. The slides were then allowed to dry and the cover slip was added using cytoseal (Thermo Fisher Scientific #527665). Slides were imaged at 60x and 500 nuclei from each mouse were called for differentiation state in a blinded fashion.

### Western Blotting

Cells were lysed with RIPA buffer (50 mmol/L Tris, 150 mmol/L NaCl, 0.5% Deoxycholate, 1% NP-40, 0.1% SDS, 1 mmol/L Dithiothreitol (DTT), and 1% protease/phosphatase inhibitor) and supernatant was cleared by centrifugation. Protein concentration was determined using the Pierce bicinchoninic acid assay kit (Thermo Fisher Scientific, 23227). Between 40 and 120 mg of each protein sample was boiled in Laemmli buffer with 10% β-Mercaptoethanol and equal percentages of each sample were run on 4%–15% polyacrylamide gels (Bio-Rad, 4561086). Resolved proteins were wet transferred to nitrocellulose membranes (Amersham), which were then blocked in a 5% BSA (VWR, 9048-46-8) solution made in 1x TBST buffer (20 mmol/L Tris base, 0.15 mol/L NaCl, 0.1% Tween, adjusted to pH 7.6). Membranes were then incubated with antibody solutions prepared in 5% BSA overnight. Antibodies used were (H3K27me3 Cell Signaling Technology C26B11 #9733S, RRID:AB_2616029 1:500, EZH2 Cell Signaling Technology 5246S, RRID:AB_10694683 1:200, B2M Millipore MABF1968, RRID:AB_2941849 1:2,000, HLA-DR,DQ,DP AbCAM ab7856, RRID:AB_306142 1:500, Total Histone H3 AbCAM ab179, RRID:AB_302613 1:5,000). After washing, secondary antibodies were added (Novus anti-Rabbit-HRP NB7160, RRID:AB_524669 and anti-Mouse-HRP NB7539, RRID:AB_10126266), incubated, and washed. Bands were visualized with West Plus Pico ECL (Thermo Fisher Scientific) and exposed to Hyperfilm ECL film (Amersham). Protein molecular weights were determined with Precision Plus Protein Kaleidoscope Prestained Protein Standards (Bio-Rad).

### MRI of Genetically Engineered Mouse Models

After adeno-Cre instillation, tumors were monitored via MRI and when tumor burden was measurable, mice were placed on one of four arms of a treatment regimen. Animals were anaesthetized via inhalation of isoflurane and kept warm on heated waterbed, vitals were monitored via cardiac and respiratory cycle (SA instruments), and recorded every 10 minutes while the animal was under anesthesia. The SA instruments pneumatic respiratory monitor was used to remove breathing artifacts by gating on the respiratory cycle. The Bruker ClinScan system used to scan the animals had 12 cm of actively shielded gradients, maximum strength 630 mT/m, and a slew rate of 6,300 T/m/second. This instrument is a 7T system with 2 × 2 array coil and 2D gradient echoT1-weighted sequences. The parameters used for imaging are as follows: 18 slices, TR = 170 ms, TE = 2.4 ms, α = 38°, Navg = 3, FOV 26 × 26 mm^2^, 1 mm thickness, matrix size 256 × 256, for a voxel size of 0.102 × 0.102 × 1.0 mm. In 2021, the system was upgraded to a Bruker Biospec system. For this upgraded machine, the Bruker IntraGate software was used to remove respiratory and cardiac motion with parameters: 18 slices, TR = 8.96ms ms, TE = 3.4 ms, α = 10°, oversampling = 28, FOV 26 × 26 mm2, 1 mm thickness, matrix size 192 × 192, 10 minutes. Models were then built on Slicer three-dimensional (3D) software to calculate tumor volume.

### Tumor Cell 3D Culture

Murine tumoroids were seeded in DMEM/F12 media (Thermo Fisher Scientific, 11330032) with an additional 4 mmol/L Glutamax (Thermo Fisher Scientific, 35050061), 5 µg/mL ITS (insulin/transferrin/selenium, SIGMA, I3146) and 8%–9% FBS (VWR, 97068-085), 12.5 mg/mL bovine pituitary extract (Invitrogen, 13028-014), 0.1 mg/mL cholera toxin (SIGMA, C-8052), 25 ng/mL mEGF (Invitrogen, 53003018), and 25 ng/mL rmFGF2 (R&D Systems, 3139-FB/CF). Tumoroids were seeded and maintained in growth factor reduced and phenol red-free Matrigel (Corning, 47743-722) in transwells with 0.4 µm pore size (Corning). Human tumoroids were seeded in DMEM/F12 media (Thermo Fisher Scientific, 11330032) with an additional 4 mmol/L Glutamax (Thermo Fisher Scientific, 35050061), 20 ng/mL FGF7 (VWR 10771-958), 50 ng/mL FGF10 (VWR 10772-106), 40 ng/mL Noggin (VWR 10772-456), 500 nmol/L A83-01 (R&D Systems 2939), 5 µmol/L Y-27632 (Abmole Y-27632), 500 nmol/L SB202190 (SIGMA S7067), B27 Supplement (Gibco 1750-44), 1.25 mmol/L N-acetylcysteine (SIGMA A9165), 5 mmol/L Nicotinamide (SIGMA N0636), and penicillin/streptomycin (Invtirogen 15140-122; ref. 19). Tumoroid cultures were established in the presence of plasmocin (Invivogen Ant-mpt-1). Tumoroids were seeded and allowed to become established before starting treatment. Tumoroids were placed on six different treatment arms: DMSO as vehicle control, GSK126 (5 µmol/L), EPZ6438 (5 µmol/L), IFNγ (20 ng/mL), or combination of GSK126 (5 µmol/L) and IFNγ (20 ng/mL) or EPZ6438 (5 µmol/L) and IFNγ (20 ng/mL). Tumoroids were fed every 2 days for 11 days total, adding in IFNγ on day 9.

### ChIP-seq

To perform ChIP analysis, we followed the ChIP Cell Fixation protocol provided by Active Motif. Cells were fixed by adding 1/10th volume of freshly prepared solution of 11% Formaldehyde (SIGMA #F-8775), 0.1 mol/L NaCl (Thermo Fisher Scientific #S271-10), 1 mmol/L Ethylenediaminetetraacetic acid (EDTA) pH 8.0 (Invitrogen #AM9261), 50 mmol/L HEPES pH 7.9 (SIGMA #H0887), diluted in H_2_O to the dissociated cell in media, and incubated for 15 minutes at room temperature. The samples were then quenched by added a 1/20th volume of 2.5 mol/L Glycine (SIGMA #G-7403). Next, the samples were washed with cold 0.5% IGEPAL CA-630 (SIGMA #I8896) in PBS pH 7.4 and centrifuged at 800 × *g* for 10 minutes at 4°C. The supernatant was removed and the cell pellets were washed in cold 0.5% IGEPAL-PBS and centrifuged for another 10 minutes at 4°C. Cells were then resuspended with 100 mmol/L phenylmethylsulfonylfluoride in ethanol, and centrifuged once again. Supernatant was removed and pellets were snap frozen with liquid nitrogen and stored at −80°C. These samples were sent to Active Motif for ChIP-seq using the antibodies H3K27me3 (Active Motif 39155, RRID:AB_2561020, 4 µL antibody per 40 µg chromatin), H3K4me3 (Active Motif 39159, RRID:AB_2615077, 4 µL antibody per 40 µg chromatin) or H3K27ac (Active Motif 39133, RRID:AB_2561016 4 µL antibody per 40 µg chromatin). Quality control and read alignment was performed by ActiveMotif. Briefly, the 75-bp single-end sequence reads were mapped to the human reference genome hg38 using the bwa samse with default settings. Reads that had >2 mismatches and multimapping reads were removed followed by PCR deduplication. The resulting bam files were normalized to account for the differences in the sequencing depth. Samples within each antibody group were reduced by random sampling to the number of unique alignments present in the smallest sample. Because the 5′-ends of the aligned reads represent the ends of the ChIP/IP fragments, the tags were extended *in silico* using Active Motif software at their 3′-ends to a length of 200 bp. To identify the density of fragments along the genome, the genome was divided into 32-nt bins and the number of fragments in each bin was determined. The MACS2 version v2.1.0 peak finding algorithm was used to identify regions of ChIP-seq enrichment over background, with *P*-value threshold of enrichment 1E-07 for all datasets. Genomic regions known to have low sequencing confidence were removed using blacklisted regions defined by the ENCODE project. The selected peak intervals were annotated to the nearest transcription start sites (TSS) using the KnownGene hg38 TSS annotation. To compare peak metrics, overlapping intervals were grouped into merged regions, defined by the start coordinate of the most upstream interval and the end coordinate of the most downstream interval. In locations where only one sample has an interval, this interval defines the merged region. Peak distribution patterns were obtained using seqplots across all merged intervals from −5 kb to +5 kb to include distal promoters and regulatory regions. Heat maps were generated for visualization of tag distributions, which are mapped across target regions. The average values for all target regions in heat maps were calculated and plotted in histograms. Peaks unique to each genotype or conserved in multiple genotypes were annotated by GREAT (20) to associate each genomic region with all genes whose regulatory domain it overlaps. The resulting gene lists were used to identify significantly enriched gene signatures from gene set enrichment analysis (GSEA) curated signature gene sets.

### Histology and HALO

Mice were euthanized and lungs were inflated with 10% buffered formalin overnight, then stored in 70% ethanol. Tissues were embedded in paraffin and sectioned at the Markey Cancer Center Biospecimens Procurement and Translational Pathology Shared Resource Facility (BPTP SRF). Hematoxylin and eosin (H&E)-stained slides were scanned at 20x or 40x with an Aperio slide scanner and images were used for further analysis. To validate that the subcutaneous grafts contained similar cellular proportions to lung tumors, we used our previously described nuclear phenotyper (21, 22) shown in Fig. 5C. CD8 IHC was performed by the BPTP SRF by staining with Ventana Discovery Ultra, onboard deparaffinization, followed by antigen retrieval with Ventana Discovery CC1 (Roche 950-500) using standard conditions. Primary antibody was applied at 1:250 dilution (Cell Signaling Technology #98941, RRID: AB_275637) at 37°C for 1 hour, followed by incubation with Ventana anti-rabbit-HQ (Roche 760-4815, RRID: AB_2811171) for 20 minutes, and Ventana anti-HQ-HRP (Roche 760-4820, RRID: AB_3068525). The staining was then amplified using Ventana Discovery TSA Amplification Kit (Roche 760-052) for 16 minutes, followed by linking with Discovery Amplification Multimer-HRP (Roche 760-4602) for 20 minutes and DAB detection. Slides were counterstained with Meyer's hematoxylin, blued, and permanently mounted. Slides were scanned at 20x and the HALO software was again used to quantify cells that were positive for CD8 stain using the same annotations delineating tumor tissue as the matched H&E slides used for the nuclear phenotyping.

### scRNA-seq

Lungs were harvested from mice and dissociated by finely mincing with scissors and triturating with a 5 mL serologic pipette filled with Ca^2+^/Mg^2+^-free PBS. To enrich for immune cell populations, they were then stained with EpCAM-PECy7 (BioLegend #118216, RRID:AB_1236471) and CD31-APC (BioLegend catalog no. 102510, RRID:AB_312917), bound to beads and run through Miltenyi LS columns (#130-042-401) on a magnet. The flow through was collected and cells were captured for reverse transcription by a 10x Genomics Chromium controller. Bone marrow cells were isolated on a histopaque gradient as described above. Reverse transcription was performed using the 10X Genomics Single Cell 3′ v3 Kit. Libraries were prepared and sequenced, and the sequencer-produced Chromium single-cell data and then the Cell Ranger toolkit version 3.1 (10X Genomics) was used to demultiplex samples from raw sequencing reads to gene-count matrices with alignment to the mm10 genome (v93). To perform the downstream analysis, such as cell type identification and differential gene expression analysis, Seurat (V3) R package (23) was employed to aggregate the gene-count matrices from all samples and provide the analytic insight. A five-step process was performed using Seurat package: (i) For quality control and data preprocessing, we discarded genes expressed in fewer than three cells and discarded the low-quality cells that had less than 100 genes expressed or percentage of mitochondrial transcripts >7. The count matrices were then log-transformed. (ii) For sample merging and feature selection, we combined the log-transformed matrices of each sample and applied the variance-stabilizing transformation (vst) method to remove cell-to-cell variation. The top 1,500 genes were selected for sample integration. (iii) For dimension reduction and clustering, we applied “PCA” to reduce the dimensionality of the merged data to 50 principal components, and then performed a shared nearest neighbor modularity optimization-based algorithm to identify clusters of cells. We utilized the Uniform Manifold Approximation and Projection (UMAP; ref. 24) technique to visualize the clustering results as shown in Fig. 6A. (iv) Cell cluster identities were called by examining highly expressed genes in each cell cluster (Supplementary Table S3). (v) For differential gene expression analysis for each cell population, we used a differential expressed gene (DEG) identification method in Seurat, namely “MAST” (25), to identify the upregulated/downregulated sets of genes between treatment groups in the three major populations, neutrophils, macrophage/dendritic cells, and tumor cells. In the Fig. 6B, the distribution of cell clusters across genotypes was assessed by z-test and *P* values were adjusted for multiple hypothesis testing.

### RNA Isolation

To perform RNA isolation Absolutely RNA Miniprep kits were used (Agilent #400805). Cell pellets were resuspended in lysis buffer plus 0.7% β-mercaptoethanol and stored at −80°C. An equal volume of 70% nuclease-free ethanol was added to each sample and the solution was added to a column. Columns were washed once with low-salt wash buffer once and DNase digestion was performed for 15 minutes at 37°C. The column was then washed once with high-salt buffer, followed by two more low-salt washes. The RNA was eluted with prewarmed elution buffer for 2 minutes and stored at −80°C.

### qRT-PCR and Sequencing

Concentration of RNA samples was quantified by NanoDrop 8000 spectrophotometer (Thermo Fisher Scientific #ND-8000-GL). To perform cDNA synthesis, random hexamers (50 ng/µL) and dNTPs (10 mmol/L) were mixed in a 1:1 ratio and 2 µL was placed in each PCR tube. Next, 1,000 ng of RNA was added and volume was brought up to 12 µL, placed in C1000 Touch thermal cycler (Bio-Rad), and run for 5 minutes at 65°C. A master mix of reagents was made for each sample (5x reverse transcription buffer, 50 mmol/L MgCl_2_ (Invitrogen #AM9530), 0.1 mol/L dithiothreitol, 40 U/µL RNaseOUT (Invitrogen #100000840), 200 U/µL SuperScript III (Invitrogen #56575). The cDNA protocol was performed on thermal cycler as follows: 25°C for 10 minutes, 50°C for 50 minutes, 70°C for 15 minutes. After the 70°C step, 1 µL of RNase H (Ambion #AM2293) was added to each tube and protocol continued for 20 minutes at 37°C. After genertion, cDNA is diluted 1:5 before performing qPCR and stored at −80°C. To perform qPCR master mixes were made for each gene of interest using TaqMan Fast Advanced Master Mix (Invitrogen #4444964) and TaqMan qPCR Assays, then run on QuantStudio 3 (applied biosystems #A28567). Data were analyzed by calculating *Gene of Interest*(Ct_reference_ − Ct_exprimental_) − *Gapdh*(Ct_reference_ − Ct_experimental_) and the data were graphed on the log_2_ scale. Library preparation and sequencing were performed by the Beijing Genomics Institute (BGI Group) using DNBseq to a depth of 24 million 100 bp paired-end reads. Sequencing reads were trimmed and filtered using Trimmomatic (V0.39; ref. 26) to remove adapters and low-quality reads. Reads from human samples were mapped to Ensembl GRCh38 transcript annotation (release 98), and mouse samples to Ensembl GRCm38 (mm10) transcript annotation (release 82), using RSEM (27). Gene expression data normalization and differential expression analysis were performed using the R package edgeR (28).

### Statistical Analysis and Reproducibility

Statistical analyses were carried out using GraphPad Prism or Microsoft Excel. Unless otherwise stated, all numerical data are presented as mean ± SEM. For graft experiments *n* = tumors or antibody stains (experimental replicates), and for autochthonous experiments, *n* = mouse or tumors, as indicated in figure legends. For grouped analyses, one-way ANOVA with Holm-Šídák multiple comparisons correction was used, and for paired analyses a two-tailed equal variance *t* test was used. A *P* value (or adjusted *P* value) less than 0.05 was considered statistically significant.

### Data Availability Statement

All data presented in this article are available from the corresponding author upon reasonable request. The sequencing data are available at NCBI Gene Expression Omnibus under the super-series GSE233665.

The RNA sequencing (RNA-seq) data are available under accession number: GSE233468
The ChIP-seq data are available under accession number: GSE233469
The scRNA-seq data are available under accession number: GSE233665

## Results

### EZH2 Inhibition Allows Upregulation of MHCI and MHCII in Multiple Models of LSCC

**FIGURE 1:**
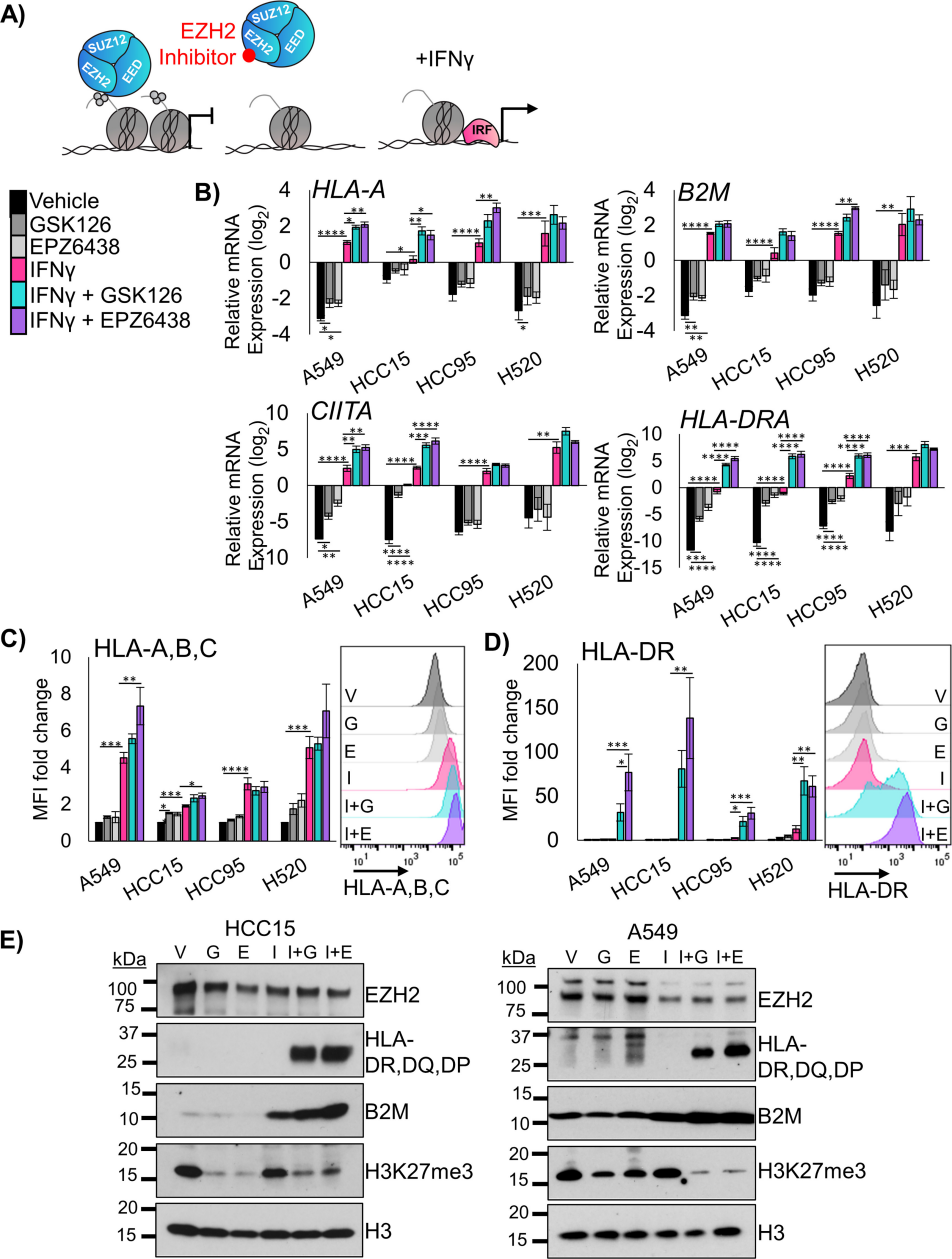
EZH2 inhibition allows upregulation of MHCI and MHCII in 2D human LSCC cell lines. **A,** Schematic for proposed mechanism: Inhibition of EZH2 methyltransferase activity by the drugs GSK126 or EPZ6438 will lead to derepression of antigen presentation genes that can then be more effectively activated by IFNγ. **B,** qRT-PCR in the indicated four human lung cancer cell lines treated for 7 days with vehicle or 5 µmol/L EZH2 inhibition with 20 ng/mL IFNγ added on day 5 for the genes *B2M*, *HLA-A*, *CIITA,* and *HLA-DRA*, mean ± SEM is graphed, *n* = 4 individual cultures, *, *P* < 0.04; **, *P* < 0.008; ***, *P* < 0.0009; ****, *P* < 0.0001 by one-way ANOVA with pairwise comparisons and Holm-Šídák *post hoc* test. **C,** Flow cytometry analysis of indicated four human lung cancer cell lines treated for 7 days with vehicle or 5 µmol/L EZH2 inhibition with 20 ng/mL IFNγ added on day 5 for the cell surface proteins HLA-A,B,C and HLA-DR; mean ± SEM is graphed; *n* = 4 individual cultures; *, *P* < 0.04; **, *P* < 0.006; ***, *P* < 0.0009; ****, *P* < 0.0001 by one-way ANOVA with pairwise comparisons and Holm-Šídák's *post hoc* test. Representative histograms from HCC15 cell lines are shown, G = GSK126, E = EPZ6438, I = IFNγ, I+G = IFNγ+GSK126, and I+E = IFNγ+EPZ6438. **D,** Western blotting of A549 and HCC15 cell lines treated for 7 days with vehicle or 5 µmol/L EZH2 inhibition with 20 ng/mL IFNγ added on day 5 for the proteins B2M, HLA-DR, DQ, DP, EZH2, H3K27me3 and total histone H3. Data are representative of two individual cultures. See also Supplementary Fig. S1.

The polycomb repressive complex 2 (PRC2) plays a central role in gene repression, including genes involved in immunogenicity (13, 15–18). To understand the tumor-cell intrinsic effects of EZH2 inhibition, we treated four different NSCLC cell lines with the EZH2 inhibitors GSK126 or EPZ6438 for 5 days, followed by 2 days of EZH2 inhibition with IFNγ. We reasoned that this would allow for the derepression of numerous loci in the cells, and that IFNγ treatment could then activate IFN responsive genes (Fig. 1A). Analysis of mRNA expression in these cultures showed that the MHCI genes *B2M* and *HLA-A* were robustly upregulated by IFNγ in all cell lines, and that EZH2 inhibition led to further *HLA-A* upregulation in three out of four cell lines (Fig. 1B). The MHCII genes *CIITA* and *HLA-DRA* showed a similar pattern, with significant stepwise increases in HLA-DRA expression with EZH2 inhibition, IFNγ treatment and combination (Fig. 1B). We also examined gene expression of *CD274* (encoding PD-L1) and the putative LSCC stem cell marker NGFR (Supplementary Fig. S1A). Consistent with our previous work (29), we observed that *NGFR* was upregulated by EZH2 inhibitor in A549 cells. PD-L1 results show that while IFNγ can reproducibly upregulate this T-cell suppressor, only in the HCC15 cell line does EZH2 inhibition drive additional expression. To examine whether the upregulation in genes led to increased cell surface protein expression, flow cytometry was used. An antibody against the MHCI proteins HLA-A,B,C showed a stepwise increase in expression levels, with the highest levels observed in IFNγ and EZH2 inhibitor cotreated cultures (Fig. 1C). NGFR showed upregulation at the protein level in three cell lines, but not in HCC95 that already expresses very high NGFR levels (Supplementary Fig. S1B). PD-L1 expression was upregulated significantly by IFNγ in A549 and HCC15 lines, and again HCC15 was the only cell line for which PD-L1 expression was further boosted by EZH2 inhibition. Strikingly, MHCII protein HLA-DR was dramatically increased in the cultures treated with a combination of IFNγ and EZH2 inhibitor, even in the H520 cells in which EZH2 inhibition drove a negligible increase in HLA-DRA mRNA (Fig. 1D). Finally, we confirmed changes in B2M and HLA-DR, DQ, DP and efficacy of EZH2 inhibition by H3K27me3 levels by Western blotting (Fig. 1E).

**FIGURE 2:**
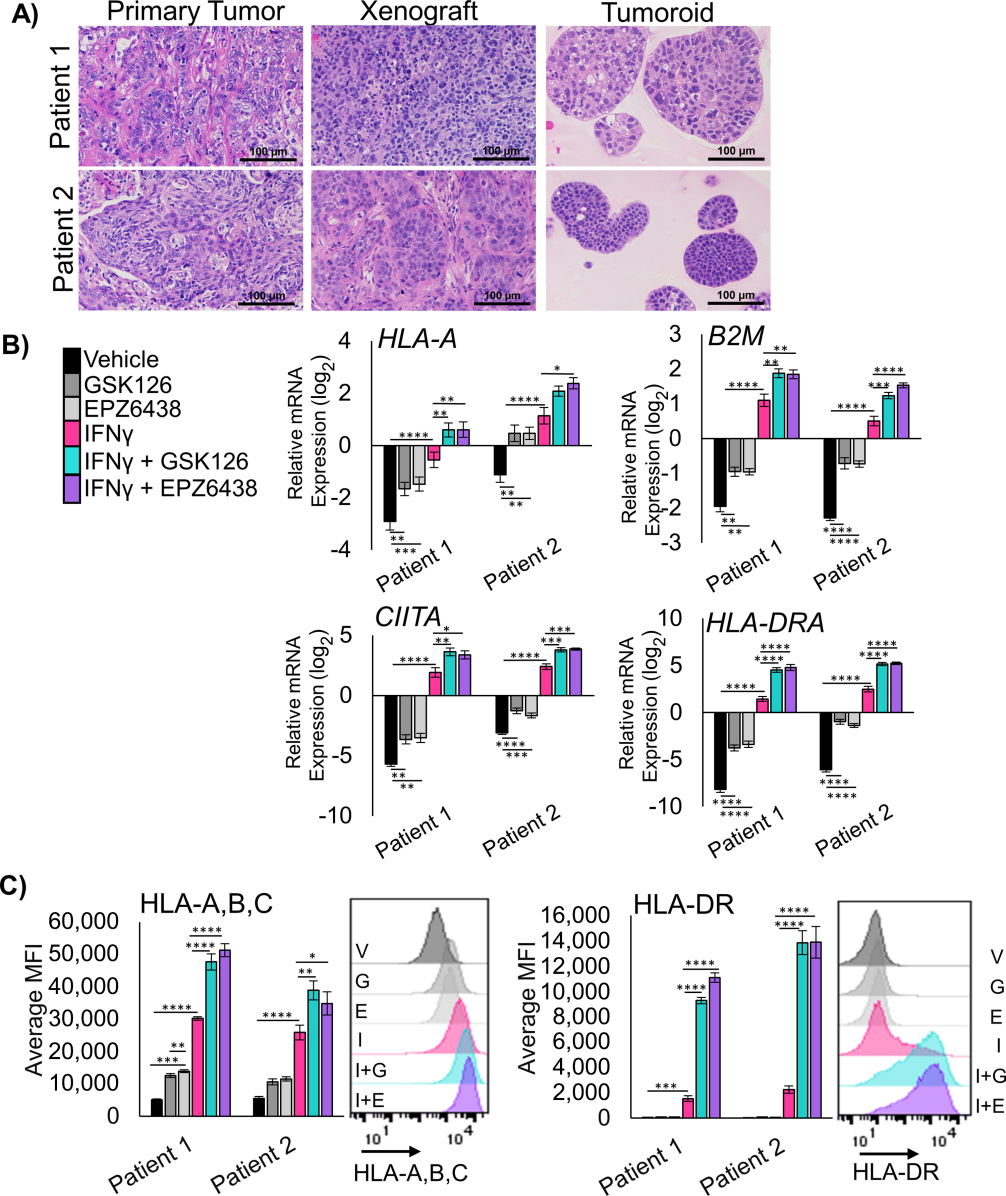
EZH2 inhibition allows upregulation of MHCI and MHCII in human LSCC PDTs. **A,** H&E staining of primary squamous cell carcinoma tissue, xenograft tissue from primary patient tissue, and tumoroids generated from xenografts, scale bars = 100 µm. **B,** qRT-PCR in two unique PDT cultures treated for 11 days with 5 µmol/L EZH2 inhibition with 20 ng/mL IFNγ added on day 9 for the genes *HLA-A*, *B2M*, *CIITA*, and *HLA-DRA*; mean ± SEM is graphed; *n* = 4; *, *P* < 0.03; **, *P* < 0.005; ***, *P* < 0.0008; ****, *P* < 0.0001 by one-way ANOVA with multiple comparisons and Holm-Šídák *post hoc* test. **C,** Flow cytometry analysis of both PDTs treated for 11 days with 5 µmol/L EZH2 inhibition with 20 ng/mL IFNγ added in on day 9 for cell surface proteins HLA-A,B,C and HLA-DR; mean ± SEM is graphed; *n* = 4 biological replicates; *, *P* = 0.043; **, *P* < 0.004; ***, *P* < 0.0009; ****, *P* < 0.0001 by one-way ANOVA with multiple comparisons and Holm-Šídák *post hoc test*. Representative histograms for Patient 1 are shown, G = GSK126, E = EPZ6438, I = IFNγ, I+G = IFNγ+GSK126, and I+E = IFNγ+EPZ6438. See also Supplementary Fig. S2.

Three-dimensional (3D) cultures allow for growth of tumor cells that cannot proliferate in 2D, and these cultures can retain the epigenetic state of *in vivo* tumors (21). Therefore, we developed two PDT cultures from distinct patients with LSCC. We treated these PDTs with the EZH2 inhibitors GSK126 and EPZ6438 for 9 days, followed by 2 days of EZH2 inhibition with IFNγ. In tumoroids treated with EZH2 inhibitor and IFNγ, B2M and HLA-A mRNA were both increased (Fig. 2A), as well as *CD274* and *NGFR* gene expression (Supplementary Fig. S2A). Similarly to the 2D cultures, the most striking results were with *CIITA* and *HLA-DRA* (Fig. 2B). By flow cytometry, both HLA-A,B,C and HLA-DR have significantly higher expression in tumoroids treated with both EZH2 inhibition and IFNγ (Fig. 2C and D). Cell surface expression of NGFR and PD-L1 proteins were changed very minimally by the treatments (Supplementary Fig. S2B). These results in 3D tumoroid cultures and the 2D cultures strongly implicate that derepression of MHCII is one of the primary outcomes of EZH2 inhibition in murine and human LSCC.

### RNA- and ChIP-seq Reveal Regulation of Both MHC and Cytokine Expression in Tumor Cells Treated with EZH2 Inhibitors

**FIGURE 3:**
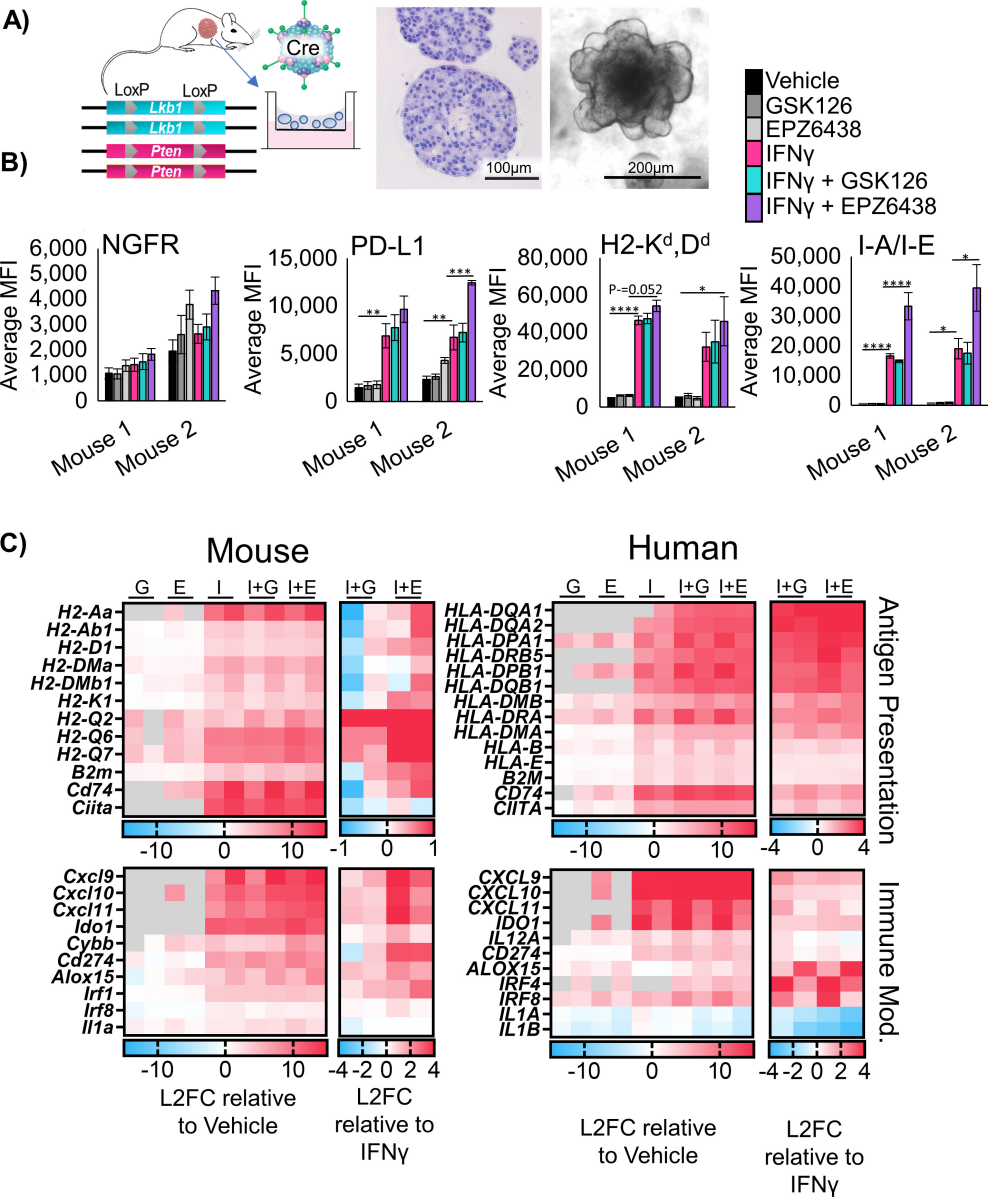
Murine LSCC organoids share derepression of MHC and pro-T cell cytokines with human models. **A,** Schematic: Generation of murine tumoroids in air-liquid interface from tumor induced in *Lkb1/Pten* mice by adenoCre administration, showing H&E stain of tumoroids, scale bar = 100 µm, and brightfield microscopy, scale bar = 200 µm. **B,** Flow cytometry analysis of two separate murine tumoroid models treated for 11 days with 5 µmol/L EZH2 inhibition with 20 ng/mL IFNγ added on day 9 stained for cell surface expression of NGFR, PD-L1, H2K^d^,D^d^, and I-A/I-E, *n* = 5 individual experiments except mouse 2 I-A/I-E and PD-L1; *n* = 4 individual experiments; *, *P* < 0.031; **, *P* < 0.006; ***, *P* = 0.0002; ****, *P* < 0.0001 by one-way ANOVA with multiples comparisons and Holm-Šídák *post hoc* test. **C**, Heat maps of log_2_ fold change in expression level from patient-derived and murine tumoroids treated for 11 days with 5 µmol/L EZH2 inhibition with 20 ng/mL IFNγ added in on day 9, G = GSK126, E = EPZ6438, I = IFNγ, I+G = IFNγ+GSK126, and I+E = IFNγ+EPZ6438. For each map, the first columns are sample 1, the second columns are sample 2. Expression relative to vehicle control (left) and relative to IFNγ only (right) are depicted. See also Supplementary Fig. S3.

To effectively test immunotherapies *in vivo*, hosts with intact immune systems must be used. Genetically engineered mouse models meet this requirement by allowing for tumor formation in the autochthonous setting. We previously reported that conditional biallelic deletion of the genes *Pten* and *Lkb1* (a.k.a *Stk11*) with inhaled adeno-Cre virus leads to lung squamous cell carcinoma (10). From these squamous tumors, we developed tumoroid cultures (Fig. 3A). Tumoroids treated with IFNγ were able to robustly upregulate all MHCI and MHCII genes tested, with further upregulation of the HLA-A ortholog *H2-K1* and *B2 m* with EPZ6438 and IFNγ (Supplementary Fig. S3A). Similar to human models, *Ngfr* was also upregulated by EZH2 inhibition. Next, we performed flow cytometry, and observed significant increases in the MHCII protein I-A/I-E, in cultures treated with IFNγ and EZH2 inhibitor relative to IFNγ alone (Fig. 3B). Furthermore, both H2-K^d^,D^d^ and PD-L1 expression were highest in tumoroids treated with both EZH2 inhibition and IFNγ.

To further confirm gene programs that are regulated by EZH2 inhibition in a conserved fashion in both mouse and human lung SCC tumoroids, we performed RNA-seq on all four tumoroid models. We compared genes upregulated by IFNγ and EZH2 inhibition relative to vehicle treatment, and genes upregulated by combination of EZH2 inhibition and IFNγ relative be to IFNγ (Fig. 3C). In addition to genes involved in antigen presentation, we observed a conservation of upregulation of the pro-T cell cytokines *CXCL9/10/11* when EZH2 inhibition and IFNγ treatments were combined. In human cells, there was also a downregulation of the proneutrophil cytokines *CXCL1/2/3*, and the cytokines *IL1A* and *IL1B* in response to treatment with EZH2 inhibition and IFNγ. We next performed GSEA (30) and observed a decrease in MYC targets, E2F targets, and DNA repair gene programs in response to EZH2 inhibition. In addition, we saw an increase in pathways involved in the inflammatory response and IFN response in response to EZH2 inhibition, and both effects were maintained when IFNγ was also added (Supplementary Fig. 3B; Supplementary Table S1).

**FIGURE 4:**
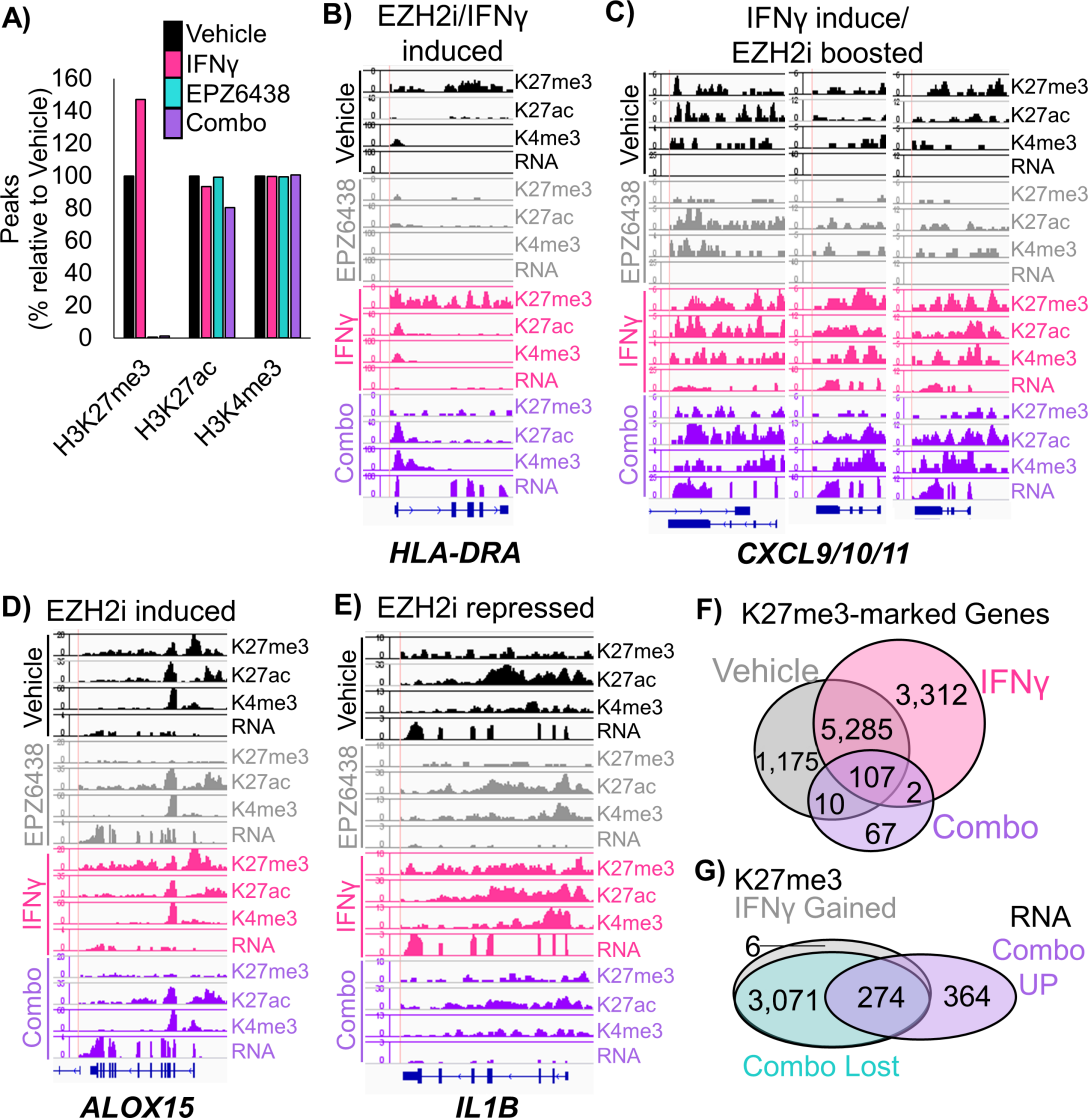
ChIP-seq of human patient-derived organoids confirms direct regulation of MHC and pro-T cell cytokines by EZH2. **A,** Peaks called at FDR 1E-7 for ChIP-seq using the chromatin marks H3K27me3, H3K27ac, and H3K4me3 in PDTs from the indicated treatment groups. Wiggle plots for H3K27me3, H3K27ac, and H3K4me3 histone mark enrichments, and matched RNA-seq tracks in PDTs from the indicated treatment groups for the genes: *HLA-DRA* (**B**), *CXCL9/10/11* (**C**), *ALOX15* (**D**), *IL1B* (**E**), H3K27me3 (**F**) peaks were called for each treatment group and GREAT was used to identify associated genes, which were then depicted by Venn diagram. **G,** H3K27me3 peaks that were gained or increased more than 2-fold with IFNγ treatment, and lost with EPZ6438 treatment were linked to associated genes by GREAT. The Venn diagram shows the overlap of these genes with those significantly upregulated in combination treated versus IFNγ-treated tumoroids. See also Supplementary Fig. S4.

To confirm the direct targets of EZH2 inhibition, we performed ChIP-seq on the Patient 1 PDT model treated with vehicle, IFNγ, EPZ6438 or a combination of IFNγ and EPZ6438. We assessed enrichment of chromatin bound to H3K27me3, H3K27ac, and H3K4me3 histone marks. We observed that IFNγ treatment increased the number of peaks bound by H3K27me3 by 47% and that H3K27me3 peaks were nearly completely ablated by treatment with EPZ6438 (Fig. 4A; Supplementary Fig. S4A and S4B). Several patterns of epigenetic gene regulation emerged from this analysis. One pattern was observed at MHCII genes, and involved a loss of H3K27me3 with EZH2 inhibitor treatment, but only with IFNγ and EZH2 inhibitor together did the loci produce transcript (Fig. 4B; Supplementary Fig. S4C). Another pattern was that IFNγ alone increased H3K27me3, H3K27ac, H3K4me3, and gene expression, and EZH2 inhibition with IFNγ boosted gene expression further by reduction of H3K27me3. This happened at loci including the *CXCL9/10/11* cluster (Fig. 4C). A smaller group of genes, including *ALOX15*, showed upregulated transcription when EZH2 was inhibited regardless of IFNγ treatment (Fig. 4D). *ALOX15* is involved in resolving inflammatory states, and therefore, may be able to reduce a protumor inflammatory environment (31). Finally, there were genes including *IL1B* that were expressed in the vehicle control cells, and transcriptionally turned off by EZH2 inhibition, despite loss of H3K27me3 at the loci (Fig. 4E). Given that IL1β is a known driver of myeloid cell recruitment and immunosuppression (32), a decrease in its expression could lead to more effective T-cell responses.

We were intrigued that ChIP-seq analysis revealed a global increase in H3K27me3 in tumoroids treated with IFNγ. In the literature, we could only find one example of this potentiation of PRC2 activity by IFNγ in human macrophages (33). We first examined peaks and associated genes that gained and lost H3K27me3 in our PDT model and contrasted vehicle, IFNγ and combo treatments. We observed that there were 3729 H3K27me3 peaks corresponding to 3,312 genes that were present only in the IFNγ-treated cells (Fig. 4F). There were also 767 H3K27me3 peaks, corresponding to 1,175 genes, unique to vehicle control cells. These results suggest that H3K27me3 peaks are being both gained and repositioned in the chromatin in response to IFNγ treatment. Next, we assessed both peaks that were gained and those that were increased more than 2-fold, and found 3773 H3K27me3-enriched peaks corresponding to 3,351 genes in IFNγ treatment compared with vehicle (Fig. 4G). Of these genes, the majority (99%) did not change in gene expression in IFNγ compared with vehicle cells, while 33 genes were upregulated and three genes were downregulated in RNA expression. Intriguingly, 989 of these genes (30%) also gained H3K27ac marks with IFNγ treatment, suggesting the PRC2 activity was directed to these genes to help repress the IFNγ response. When EZH2 inhibition was combined with IFNγ, of the 3,773 peaks corresponding to 3,351 genes that gained H3K27me3 with INFγ treatment, all but eight peaks corresponding to six genes were lost. In the combination treatment, of the 638 genes that were significantly upregulated compared with IFNγ treatment in these same tumoroids, 43% (274) of the genes had gained H3K27me3 peaks with IFNγ treatment and lost those peaks with combo treatment, with 32% (206) of the genes also gaining H3K27ac peaks. The major pathways for the 274 genes regulated in this fashion included Inflammatory responses, Cell adhesion and signaling, TP53 and apoptosis, Nervous system, and Polycomb targets (Supplementary Table S2). Together, these data indicate that IFNγ treatment increases Polycomb-mediated gene repression, which can be relieved by treatment of cells with EZH2 inhibitor and drive cells to become more immunogenic.

### Treatment of LSCC Tumor-bearing Mice with EZH2 Inhibitor and Anti-PD1 Results in Strong Tumor Control

**FIGURE 5:**
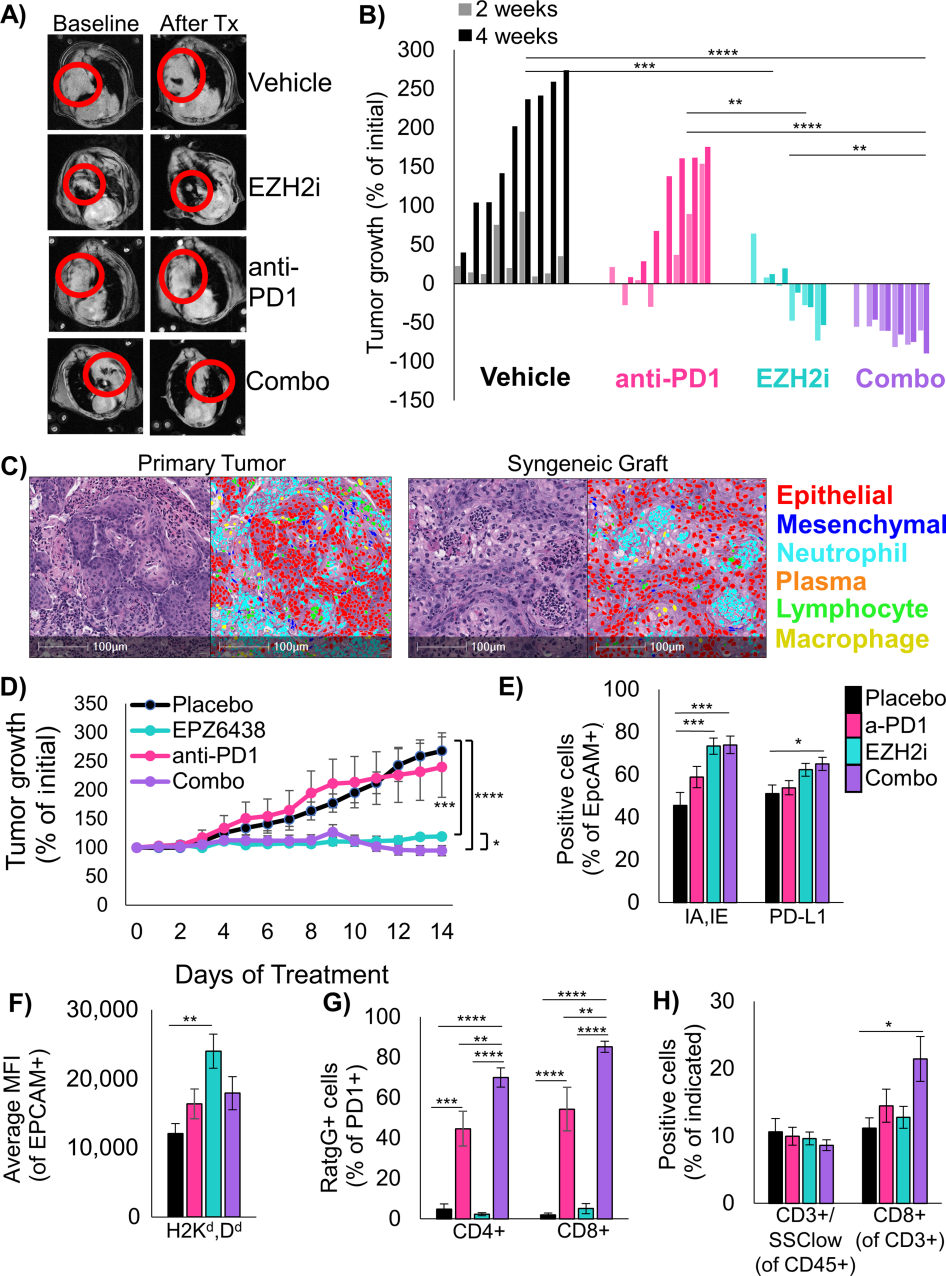
EZH2 inhibition alone and combined with immunotherapy is effective at controlling tumor burden in mouse models of LSCC. **A,** Representative MRI scans of autochthonous mice from each treatment arm at baseline and after treatment. **B,** Waterfall plot showing change in tumor volume for each mouse on all treatment arms, ****, *P* < 0.0001; ***, *P* < 0.001; **, *P* < 0.01; *, *P* < 0.05 by one-way ANOVA with multiple comparisons and Holm-Šídák *post hoc* test on log_2_-transformed values. **C,** H&E and HALO nuclear phenotyper images showing the cells within an autochthonous *Lkb1/Pten* tumor and a syngeneic graft seeded from *Lkb1/Pten* tumoroids. **D,** Percentage tumor growth from the syngeneic mouse model during 14 days of indicated treatments. ***, *P* = 0.0004; ****, *P* < 0.0001 by one-way ANOVA with multiple comparisons and Holm-Šídák *post hoc* test, *, *P* = 0.024 by two-tailed *t* test on log_2_-transformed values, Mice/tumors n are placebo = 4/8, EPZ6438 = 5/9, anti-PD1 = 6/8, combo = 5/9, mean ± SEM. is plotted. **E,** Flow cytometry analysis of dissociated tumors from the syngeneic grafts from the indicated treatment arms at day 14. Percentage of EpCAM+ cells expressing IA/IE or PD-L1 are graphed, mean ± SEM is plotted, placebo *n* = 7, EZH2 inhibitor *n* = 7, anti-PD1 *n* = 8, combo *n* = 7 with two experimental replicates each, *, *P* = 0.035; ***, *P* = 0.0008 by one-way ANOVA with multiple comparisons and Holm-Šídák *post hoc* test. **F,** From the same tumor grafts, MFI for HLA-A in the EpCAM+ cells was graphed, mean ± SEM is plotted, placebo *n* = 7, EZH2 inhibitor *n* = 7, anti-PD1 *n* = 8, combo *n* = 7 with 2 experimental replicates each, **, *P* = 0.0015 by one-way ANOVA with multiple comparisons and Holm-Šídák *post hoc* test. **G,** From tumor grafts, PD1+/CD3+/CD4+ cells and PD1+/CD3+/CD8+ were gated and percentage of cells bound to Rat-IgG2A antibody are graphed, mean ± SEM is plotted, placebo *n* = 6, EZH2 inhibitor *n* = 6, anti-PD1 *n* = 7, combo *n* = 7; **, *P* < 0.006; ***, *P* = 0.0001; ****, *P* < 0.0001 by one-way ANOVA with multiple comparisons and Holm-Šídák *post hoc* test. **H,** From the grafts, percentage of CD3+/SSC-low cells within the CD45+ fraction and percentage of CD8+ cells within the CD3+ fraction were graphed, please see Supplementary Fig. S5C for representative gates, placebo *n* = 8, EZH2 inhibitor *n* = 8, anti-PD1 *n* = 9, combo *n* = 7 with two experimental replicates each, *, *P* = 0.0197; ****, *P* < 0.0001 by one-way ANOVA with multiple comparisons and Holm-Šídák *post hoc* test. See also Supplementary Fig. S5.

To study the effects of EZH2 inhibition in combination with immunotherapy *in vivo*, we induced tumors to grow in the lungs in *Lkb1/Pten* mice by adeno-Cre inhalation and after 40–50 weeks, we randomized tumor-bearing mice onto four treatment arms. We treated mice for 4 weeks with magnetic resonance imaging of the thoracic cavity at baseline, 2 weeks, and 4 weeks to quantify lung tumor burden (Fig. 5A). Consistent with *LKB1* mutation predicting poor response to immunotherapy (34–36), anti-PD1 treatment alone had only a small impact on tumor growth (Fig. 5B). In contrast, treatment of mice with the EZH2 inhibitor GSK126 showed excellent tumor control with some tumor regression, and treatment with EZH2 inhibitor and anti-PD1 lead to significant tumor regression in all mice tested. Although these results were exciting, the experiments did not use the newly FDA-approved EZH2 inhibitor tazemetostat. To test tazemetostat, we established a syngeneic graft model by injecting *Lkb1/Pten* tumoroids into the flanks of the parental mouse strain and observed tumors forming over the course of 2 months. By nuclear phenotyping, we observed that graft tumors closely resembled those in the autochthonous model, including the predominant infiltration of neutrophils (Fig. 5C; Supplementary Fig. S5A). After 14 days of treatment, tumors treated with EPZ-6438 alone had not increased in volume, and combination treated tumors grew initially, but began to regress at day 9, and ended with slightly lower tumor volumes than EZH2 inhibition alone (Fig. 5D). Although this difference was significant for relative tumor growth, the effect size was very small and overall tumor sizes did not differ between the EZH2i and combo groups at endpoint (Supplementary Fig. S5A). We also observed, by nuclear phenotyping on residual tumors, an increase in the percentage of lymphocytes in both the syngeneic grafts and autochthonous tumors from combination treated mice compared with placebo-treated mice (Supplementary Fig. S5B). Curiously, by IHC stain for CD8 is residual tumors, CD8^+^ cell abundance was dramatically higher in the subcutaneous grafts than in the autochthonous lung tumors (Supplementary Fig. S5C). Although CD8 percentages were not higher in combination treated mice using this assay, it is important to note one important caveat of this approach was that the smallest tumors, where tumor reduction was most robust, were used for flow cytometry and sequencing assays described in the next sections. Therefore, the residual tumors are biased for the larger tumors that did not shrink.

To better understand the cell types present, grafts from each treatment arm were dissociated and analyzed by flow cytometry for a panel of immune cell markers (Supplementary Fig. S5D). Consistent with our *in vitro* data, tumors in mice treated with both EZH2 inhibitor and anti-PD1 had a significant increase in MHCII I-A/I-E positive cells, with a smaller less significant increase in PD-L1 positive cells (Fig. 5E). In the EZH2 single agent–treated mice, tumor cells showed higher levels of H2-K^d^D^d^, which were reduced in the combination treated tumors potentially through targeting and clearance of MHC-high cells (Fig. 5F). MHCI downregulation has been identified as a mechanism of immune checkpoint inhibitor resistance (37), and so the cells surviving combination treatment may have overcome treatment through this mechanism. With this flow cytometry panel, we were also able to validate the binding of the anti-PD1 antibody to PD1 positive CD4- and CD8-positive cells. Interestingly, T cells in the EZH2 inhibitor with anti-PD1 treated mice had higher percentages of PD1+ cells bound to antibody than anti-PD1 only treated mice (Fig. 5G). Although total T-cell proportions were not different, there was a significant increase in CD8^+^ T cells in combination treated mice compared with placebo (Fig. 5H). There was also a trend toward fewer CD11B+/Ly6G+ or Ly6G+/F4/80–tumor-associated neutrophils, but these differences did not reach significance. One population that was significantly increased in the combination treated mice relative to all other cohorts was a Ly6G+/F4/80+ myeloid cell, and this could indicate a conversion of neutrophils into macrophages by EZH2 inhibition which is a phenomenon described in both human and mouse (refs. 38, 39; Supplementary Fig. S5E). To test whether the immune system was a critical mediator of EPZ6438 single therapy response, we injected the same tumoroid line used to generate syngeneic grafts into NSG mice. Surprisingly, although we injected the same cell preparations only an hour later into parental mice, the 6 NSG mice we injected had no tumor formation over the course of 4 months (Supplementary Fig. S5F). This result suggests a requirement of a functional immune system for these tumoroids to form grafts—a phenomenon that we will further explore in future studies.

### scRNA-seq Confirm Mechanisms Through Which EZH2 Inhibition Drives Increased Tumor Immunogenicity

**FIGURE 6:**
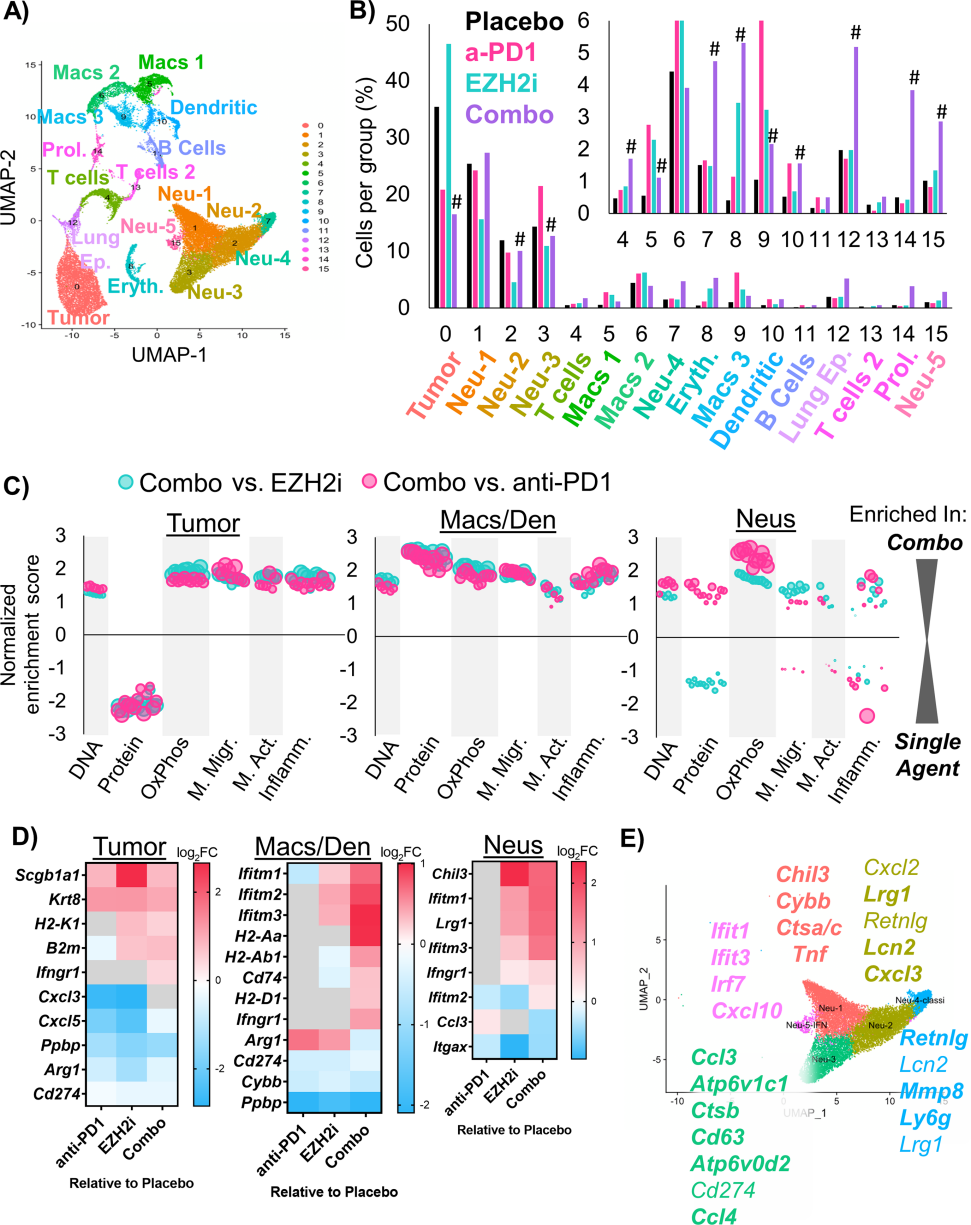
scRNA-seq highlights neutrophil heterogeneity shifts in response to EZH2 inhibition combined with immunotherapy. **A,** Annotated UMAP plot showing the 16 different populations within lung tumors of the *Lkb1/Pten* model of LSCC after treatment with placebo, GSK126, anti-PD1, or combined GSK126 with anti-PD1. **B,** Percentage of cells per treatment group graphed for all populations, # indicates adjusted *P* < 0.0012 by proportion z-test. **C,** GSEA depicting gene sets that are enriched or depleted in Tumor, Macrophage/Dendritic Cells, or Neutrophils in mice treated with EZH2 inhibitor and anti-PD1 contrasted with either treatment alone. Normalized enrichment scores are plotted and bubble sizes estimate FDR. See also Supplementary Table S2. **D,** Heat maps showing DEGs among tumor, macrophages, and dendritic cells, and neutrophils between GSK126, anti-PD1, and combination treated mice compared with placebo. **E,** UMAP of five neutrophil populations showing selected genes that are highly expressed in each cluster. See also Supplementary Fig. S6.

Finally, to assess the transcriptional heterogeneity of cell types in the tumors and to learn how transcriptional programs were changed by treatment, we performed scRNA-seq. We analyzed fresh autochthonous lung tumors after 4 weeks of therapy, and lungs of mice that were on placebo or EZH2 inhibition for 2 weeks that had no tumors as controls. From the tumors and total lungs, we identified 16 unique cell populations that we annotated on the basis of conserved markers (Fig. 6A; Supplementary Table S3). Analysis of cell proportions in each treatment group revealed significant decreases in tumor cells and some macrophage and neutrophil populations, and significant increases in T cells, cycling cells, normal lung, and some neutrophil populations (Fig. 6B). Next, we analyzed the DEGs in three major groups, the tumor cells (malignant epithelial cells), the macrophage and dendritic cells, and the neutrophils. By GSEA, we observed a dramatic decrease in protein synthesis pathways in tumors cells from combination treatment compared with either single treatment, and increases in DNA pathways, which could reflect cell cycle arrest or DNA damage. Pathways involved in oxidative phosphorylation were upregulated in all three cell populations, and pathways involved in IFN signaling were upregulated in the tumor cells and the macrophage/dendritic cells with combination treatment (Fig. 6C; Supplementary Fig. S6A; Supplementary Tables S4 and S5). To understand the individual genes changed by treatment, we also assessed differential expression of each cell type in the treated groups relative to placebo (Fig. 6D). Tumor cells that were treated with EZH2 inhibitor alone or in combination with anti-PD1 had increased *H2-K1*, *B2m*, and *Ifngr1* expression, and decreased expression of neutrophil-recruiting chemokines such as *Cxcl3*, *Cxcl5*, and *Ppbp* (a.k.a *Cxcl7*). Moreover, macrophage and dendritic cells had increased MHCII and IFN-response genes, while neutrophils showed upregulation in IFN-response genes in the combo group relative to both EZH2 inhibition and anti-PD1 treatments alone.

It was surprising that the neutrophil populations remained predominant in tumors treated with EZH2 inhibition and immunotherapy, given the large amount of data suggesting that neutrophils prevent proper immunotherapy response. Therefore, we next interrogated the gene expression profiles of the five identified neutrophil populations (Fig. 6D; Supplementary Table S6). On the basis of the literature (11), we believe that there are three populations of neutrophils in our dataset that may promote tumor elimination. These three populations, Neu1, Neu4, and Neu5, express genes such as *Tnf*, *Cxcl10*, and multiple IFN-response genes. Interestingly, we observed increases in these populations in the group treated with the combined therapy. Moreover, we saw significant decreases in Neu2 and Neu3 populations, which we believe to be immunosuppressive. These two populations expressed genes *Cxcl3*, *Ccl3*, *Ccl4, Atp6v1c1*, *Atp6v0d2*, which have been associated with a tumor promoting phenotype. However, Neu3 also has an MHCII antigen presentation gene (*H2-Eb1*), suggesting it may be a “hybrid” tumor promoting and tumor eliminating phenotype. Demonstrating a conservation among models, genes bolded in our figure correspond to genes identified by other groups in neutrophil populations from lung cancers by scRNA-seq (40–42).

To understand whether neutrophils were also altered in the bone marrow when an EZH2 inhibitor was administered, we performed scRNA-seq on the bone marrow of the same mice used for the tumor analysis (Supplementary Fig. S6A). Using markers from a recent scRNA-seq report on neutrophil development (43), we observed that Neu1 is the most mature, with Neu6+7 containing myelocytes and promyelocytes (Supplementary Fig. S6B). Our Neu3 population was enriched for expression of *Mmp8* and *Retnlg*, which corresponded to band cells in the previous article. Using this information, we marked Neu1+2 as mature and hypersegmented, Neu3 as banded, BM-Neu4 as meta-myelocyte, Neu5 as myelocyte, and Neu6–8 as promyelocytes. By proportion analysis, significantly more neutrophils were mature, and significantly fewer neutrophils were in the meta-myelocyte stage in mice treated with EZH2 inhibitor alone or in combination with anti-PD1 compared with placebo-treated mice (Supplementary Fig. S6C). These EZH2 inhibitor induced changes were also observed in the non–tumor-bearing control mice, suggesting that the effect of EZH2 inhibition on the bone marrow is tumor independent (Supplementary Fig. S6D). To validate this result, we also examined nuclear morphology of the populations by cytospin, and again observed more mature and fewer myelocyte cells in EZH2 inhibitor–treated mice compared with placebo-treated mice (Supplementary Fig. S6E). Similarly, bone marrow cells isolated from the syngeneic graft-bearing mice treated with EZH2 inhibitor were more apoptotic, suggesting a more mature phenotype (Supplementary Fig. S6F). Together, these data show a systemic shift in neutrophil identity away from an immature myeloid suppressor cell phenotype toward a more mature tumor eliminating phenotype, and suggest that neutrophils can be compatible with immunotherapy in the right contexts.

## Discussion

**FIGURE 7:**
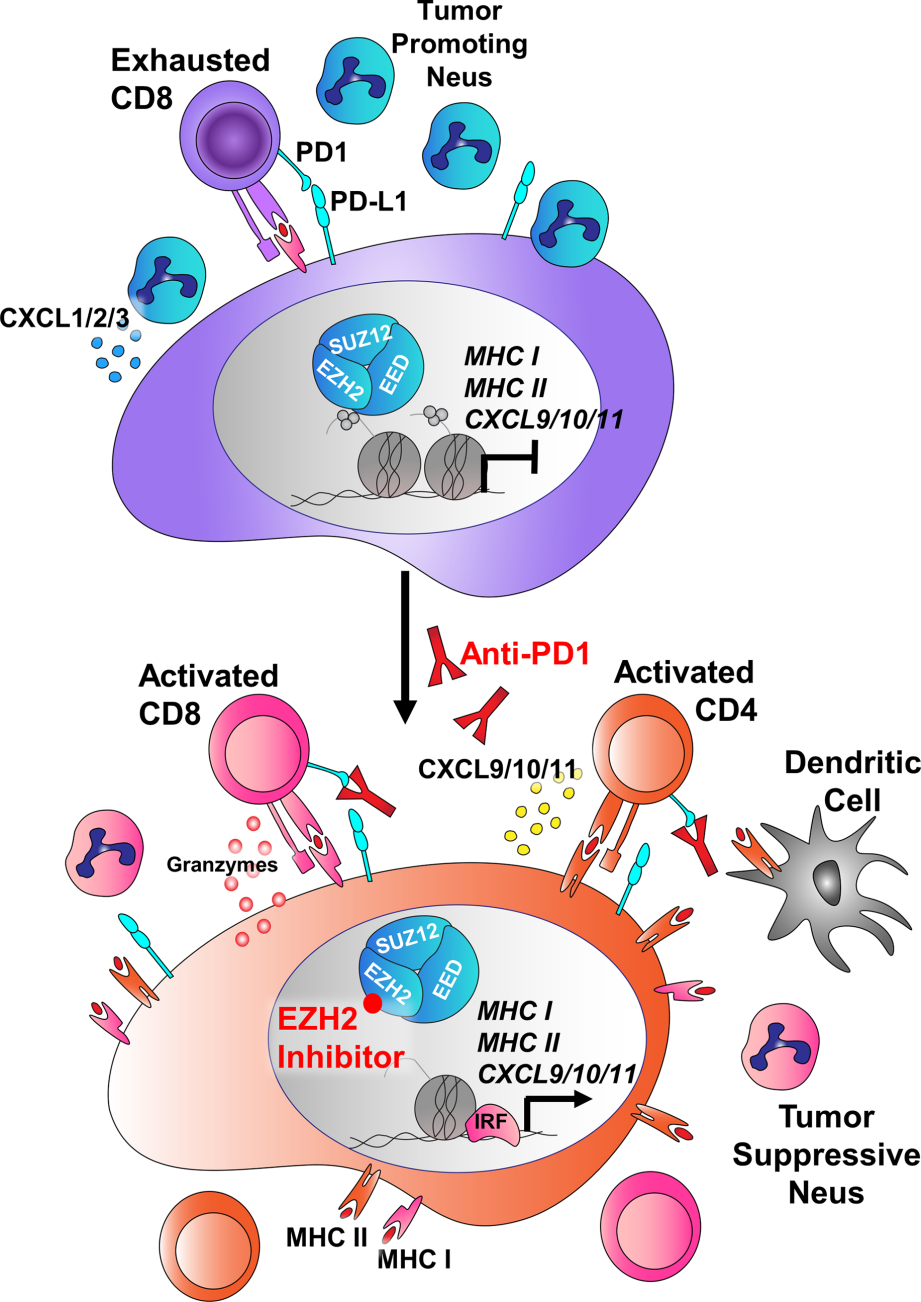
Schematic of tumor cell-intrinsic and microenvironmental consequences of EZH2 inhibition that boost immunotherapy response in LSCC. In LSCC, tumors can evade the immune system through expression of PD-L1, inhibiting T-cell activation. In addition, these tumors secrete high levels of *CXCL1/2/3* (mouse orthologs *Cxcl3/5/7*) that attract T cell suppressive neutrophils, and can express high levels of arginase, that further drive T-cell suppression. In response to EZH2 inhibition, the tumors upregulate MHCI and MHCII antigen presentation machinery, and switch from expression of *CXCL1/2/3* to expression of the T cell promoting cytokines *CXCL9/10/11* and the inflammatory resolution molecule *ALOX15*. *IL1B* and *Arg1* are downregulated, and the neutrophils surrounding the tumor take on more tumor-repressive phenotypes. When anti-PD1 antibody is added, the net result is tumor regression through immune targeting of tumor cells.

Here, we demonstrate that inhibition of EZH2 in LSCC can boost immunotherapy in several ways (Fig. 7). In both 2D and 3D *in vitro LSCC* models, treatment with EZH2 inhibition and IFNγ led to increases in MHCI/II and proinflammatory cytokine expression. In 3D PDTs, ChIP-seq confirmed a switch from repressive to active chromatin at these genes in response to treatment. Studies have found that *IFNG*, *CXCL9*, and *CD274* expression in tumor specimens correlate to stronger immunotherapy responses (44). Mirroring data in urothelial cells (15), we found that the pro-T cell cytokines *CXCL9/10/11* are strongly induced by a combination of IFNγ and EZH2 inhibition in both human and murine tumoroids, and that this gene cluster is a direct PRC2 target in human lung cancer cells. Next, we employed both autochthonous and syngeneic models of LSCC driven by biallelic deletion of the tumor suppressors *Lkb1* and *Pten*. We observed significant tumor control in both the anti-PD1 with EZH2 inhibitor combination, as well as EZH2 inhibition alone. Using scRNA-seq and immune cell profiling, we identified increases in MHCI/II expression and a shift toward tumor-eliminating neutrophils within tumors. Furthermore, the T-cell suppressive *Arg1* was significantly downregulated in autochthonous tumors treated with EZH2 inhibition. Finally, we found that neutrophil populations shifted toward IFN-responsive and TNFα-expressing populations in tumors, and that neutrophils in the bone marrow, even in tumor-free mice, had more mature phenotypes in response to EZH2i. These data point to multiple overlapping mechanisms through which immunologically “cold” tumors can be induced to become “hot” through EZH2 inhibition.

In addition to strongly influencing the tumor immune microenvironment, EZH2 inhibition drove dramatic increases in expression of both MHCI and MHCII *in vitro*. Several studies have also observed that EZH2 plays a major role in repression of MHCI in head and neck, small cell lung cancer and melanoma (16–18), and MHCII in urothelial and AML cancers (15, 45). Furthermore, MHCII expression correlates with increased ICI response rates in melanoma, and depletion of MHCII in a NSCLC can covert tumors from ICI sensitive to resistant (14, 46). While anti-PD1 ICI is thought to activate CD8^+^ T cells to target the tumor through MHCI interactions, CD4^+^ T cells can kill MHCII+ cells though FAS/FASL interactions. Given that EZH2 inhibition appears to regulate both MHCI and class II in squamous cell carcinoma cells, this may help to delay or prevent acquired resistance that occurs more readily when only one molecular pathway is altered. Furthermore, our data suggest an efficacy of EZH2 inhibition as a single therapy in our model *in vivo*, and this observation could be used to test EZH2 inhibition as a single agent clinically in patients with LSCC who cannot tolerate immunotherapy.

Importantly, the model we used is deficient for LKB1, which is correlated with immunotherapy resistance and in accordance with this clinical observation, the model had only a minimal response to anti-PD1 as a single therapy (34–36). However, most of these data are from non-squamous histology tumors and may rely upon KRAS mutational status (47). Our data suggest that when an LKB1-deficient tumor is squamous in epigenetic state, that EZH2 inhibition combined with immunotherapy will be an effective treatment approach. What is less clear is whether tumors that are adenocarcinoma in histology will respond as effectively, and more research is needed. MHCI and MHCII expression are intrinsic properties of specific alveolar and bronchiolar lung cells (48, 49). Therefore, these molecules may already be expressed in adenocarcinomas, depending on the tumor cell-of-origin and EZH2 inhibition may not be able to boost the expression levels. We also focused on models with significant neutrophil infiltration. Our recent study of human lung cancers showed that macrophages and plasma cells can also predominate in tumors (22), and it will be important to test EZH2 inhibition in the context of diverse tumor immune microenvironments moving forward.

One important limitation of this study is the correlative nature of our *in vivo* experiments that do not separate tumor cell-intrinsic from immune cell–dependent mechanism of EZH2 inhibition. While our *in vitro* studies indicate that EZH2 inhibition and IFNγ are both required for MHCII upregulation, *in vivo* the changes seen in the tumor cells may be secondary to immune modulation by EZH2 inhibition. Future studies will focus on understanding whether T-cell activation and subsequent IFNγ signaling are major drivers of increased MHCI and MHCII on tumor cells *in vivo*, and whether cells other than T cells, such as neutrophils, are required for tumor stasis in response to EZH2 inhibitor. Another aspect of immunotherapy response that was not addressed in the current study is the role of tumor mutation burden and neoantigens. In general, genetically engineered mouse models are thought to have relatively low tumor mutation burden (TMB) (50). However, studies have suggested that EZH2 inhibition can increase genotoxic stress in other models (51), and this idea should be explored in lung cancer. The effect of EZH2 inhibition in models with varied TMBs would also be worthwhile. Despite this limitation, this work demonstrates several mechanisms through which EZH2 inhibition can boost ICI responses in lung cancer. The EZH2 inhibitor tazemetostat was recently FDA approved, and is already in clinical trials with ICIs for urothelial tumors (NCT03854474). Importantly, these data suggest that EZH2 inhibition plus ICI, or EZH2 inhibition alone could be viable options specifically for LSCC, and these data serve as strong premise for clinical investigation. Finally, the model systems we have characterized will be useful tools to explore further mechanisms of ICI response and resistance in LSCC.

## Supplementary Material

Supplementary Table 1 Supplementary Table 1 shows Gene Set Enrichment Analysis for differentially expressed mRNAs in mouse and human lung squamous cell carcinoma tumoroids treated with EZH2 inhibitor and IFN-gamma.

Supplementary Figure 1 Supplementary Figure 1 shows changes in NGFR and CD274 (PD-L1) gene and protein expression in human lung cancer cell lines in response to EZH2 inhibitor and interferon-gamma treatment.

Supplementary Table 2 Supplementary Table 2 shows Gene Set Enrichment Analysis on genes with mRNA up-regulated and H3K27me3 peaks lost in combination treatment vs IFN-gamma alone in human lung squamous cell carcinoma tumoroids.

Supplementary Figure 2 Supplementary Figure 2 shows changes in NGFR and CD274 (PD-L1) gene and protein expression in human lung cancer tumoroids in response to EZH2 inhibitor and interferon-gamma treatment.

Supplementary Table 3 Supplementary Table 3 shows gene highly expressed in each of the 16 clusters called in the single cell RNA-sequencing from murine lung and lung squamous cell carcinoma samples.

Supplementary Figure 3 Supplementary Figure 3 shows changes in gene expression and Gene Set Enrichment Analysis in lung squamous cell carcinoma tumoroids in response to EZH2 inhibitor and interferon-gamma treatment.

Supplementary Table 4 Supplementary Table 4 shows Gene Set Enrichment Analysis of differentially expressed mRNAs in combination-treated tumors vs. single agent-treated tumors for three major cell sub-clusters in the single cell RNA-sequencing.

Supplementary Figure 4 Supplementary Figure 4 shows additional ChIP-sequencing data from human lung squamous cell carcinoma tumoroids.

Supplementary Table 5 Supplementary Table 5 shows Gene Set Enrichment Analysis of differentially expressed mRNAs in therapy-treated tumors vs. placebo-treated tumors for three major cell sub-clusters in the single cell RNA-sequencing.

Supplementary Figure 5 Supplementary Figure 5 shows additional analysis of tumors from EZH2 inhibition and anti-PD1 treated mice.

Supplementary Table 6 Supplementary Table 6 shows gene highly expressed in each of the 4 neutrophil clusters called in the single cell RNA-sequencing from murine lung and lung squamous cell carcinoma samples.

Supplementary Figure 6 Supplementary Figure 6 shows analysis of bone marrow from mice treated with EZH2 inhibition and anti-PD1.
