# Supplementary material for: EZH2 Inhibition Promotes Tumor Immunogenicity in Lung Squamous Cell Carcinomas
Source: Cancer Res Commun. 2024 Feb 13;4(2):388–403. doi: 10.1158/2767-9764.CRC-23-0399 (PMC10863487; doi:10.1158/2767-9764.CRC-23-0399)
Supplement: Supplementary Table 1 — shows Gene Set Enrichment Analysis for differentially expressed mRNAs in mouse and human lung squamous cell carcinoma tumoroids treated with EZH2 inhibitor and IFN-gamma. [file crc-23-0399-s01.pdf]

Supplemental Table 1: GSEA on RNAseq of Tumoroids, related to Figure 3
NES=Normalized Enrichment Score, FDR=False Discovery Rate

MSigDB Signature Name	Patient 1		Patient 2		Patient 1		Patient 2	
	GSK126 vs Veh		*GSK126 vs Veh*		*EPZ6438 vs Veh*		*EPZ6438 vs Veh*	
	NES	FDR q-val	NES	FDR q-val	NES	FDR q-val	NES	FDR q-val
HALLMARK_MYC_TARGETS_V1	-2.274	0.000	-2.505	0.000		1.000	-2.127	0.000
HALLMARK_MYC_TARGETS_V2	-1.482	0.079	-1.940	0.014	-1.711	0.017	-1.743	0.026
HALLMARK_DNA_REPAIR	-1.356	0.085	-1.413	0.097	-0.833	0.644	-1.207	0.217
HALLMARK_UNFOLDED_PROTEIN_RESPONSE	-1.360	0.096	-1.273	0.160	-1.542	0.039	-1.142	0.268
HALLMARK_E2F_TARGETS	-0.922	0.688	-1.357	0.117		1.000	-0.632	0.966
HALLMARK_REACTIVE_OXYGEN_SPECIES_PATHWAY	-1.868	0.008	-1.594	0.058	-1.818	0.007	-1.625	0.035
HALLMARK_OXIDATIVE_PHOSPHORYLATION	-2.657	0.000	-2.152	0.000	-2.161	0.004	-2.125	0.000
HALLMARK_INFLAMMATORY_RESPONSE	1.501	0.025	0.821	0.927	1.242	0.183	-1.659	0.036
HALLMARK_INTERFERON_GAMMA_RESPONSE	1.273	0.164	0.999	0.793	1.479	0.036	0.639	1.000
	GSK+IFN vs IFN		*GSK+IFN vs IFN*		*EPZ+IFN vs IFN*		*EPZ+IFN vs IFN*	
	NES	FDR q-val	NES	FDR q-val	NES	FDR q-val	NES	FDR q-val
HALLMARK_MYC_TARGETS_V1	-1.753	0.024	-2.284	0.000	-1.801	0.026	-2.111	0.000
HALLMARK_MYC_TARGETS_V2	-1.527	0.062	-2.244	0.000	-1.637	0.051	-2.139	0.000
HALLMARK_DNA_REPAIR	-1.064	0.420	-1.540	0.069	-1.121	0.361	-1.419	0.117
HALLMARK_UNFOLDED_PROTEIN_RESPONSE	0.892	0.977	-0.829	0.814	0.716	1.000	-0.648	0.965
HALLMARK_E2F_TARGETS	-1.001	0.478	-1.530	0.054	-0.480	1.000	-0.730	0.984
HALLMARK_REACTIVE_OXYGEN_SPECIES_PATHWAY	-1.763	0.032	-1.321	0.149	-1.806	0.038	-1.398	0.096
HALLMARK_OXIDATIVE_PHOSPHORYLATION	-2.091	0.002	-1.393	0.107	-2.064	0.000	-1.557	0.067
HALLMARK_INFLAMMATORY_RESPONSE	1.503	0.036	0.882	0.934	1.178	0.421	-1.413	0.100
HALLMARK_INTERFERON_GAMMA_RESPONSE	1.722	0.002	1.066	0.698	1.385	0.118	0.912	1.000
	**Mouse 1**		**Mouse 2**		**Mouse 1**		**Mouse 2**	
	GSK126 vs Veh		*GSK126 vs Veh*		*EPZ6438 vs Veh*		*EPZ6438 vs Veh*	
	NES	FDR q-val	NES	FDR q-val	NES	FDR q-val	NES	FDR q-val
HALLMARK_MYC_TARGETS_V1	-1.948	0.001	-1.404	0.086	-1.701	0.089	-1.465	0.092
HALLMARK_MYC_TARGETS_V2	-1.635	0.017	-1.463	0.064	-1.543	0.110	-0.778	1.000
HALLMARK_DNA_REPAIR	-1.572	0.025	-0.815	0.981	-1.542	0.073	-1.315	0.167
HALLMARK_UNFOLDED_PROTEIN_RESPONSE	-1.348	0.112	-1.000	0.690	-1.313	0.178	-0.693	0.931
HALLMARK_E2F_TARGETS	-2.393	0.000	-2.560	0.000	-0.597	0.980	-2.231	0.003
HALLMARK_REACTIVE_OXYGEN_SPECIES_PATHWAY	-1.054	0.424	0.979	0.591	-1.317	0.218	0.695	0.960
HALLMARK_OXIDATIVE_PHOSPHORYLATION	0.534	0.998	0.499	0.999	-1.198	0.258	-0.728	1.000
HALLMARK_INFLAMMATORY_RESPONSE	2.178	0.000	1.233	0.227	1.811	0.000	1.662	0.006
HALLMARK_INTERFERON_GAMMA_RESPONSE	1.772	0.014	1.614	0.032	1.775	0.001	1.811	0.000
	GSK+IFN vs IFN		*GSK+IFN vs IFN*		*EPZ+IFN vs IFN*		*EPZ+IFN vs IFN*	
	NES	FDR q-val	NES	FDR q-val	NES	FDR q-val	NES	FDR q-val
HALLMARK_MYC_TARGETS_V1	-1.618	0.050	-2.248	0.000	-2.003	0.008	-2.443	0.000
HALLMARK_MYC_TARGETS_V2	-2.020	0.007	-2.006	0.000	-1.930	0.007	-1.969	0.005
HALLMARK_DNA_REPAIR	-0.938	0.889	-1.046	0.469	-1.350	0.144	-1.591	0.030
HALLMARK_UNFOLDED_PROTEIN_RESPONSE	-1.559	0.044	-1.936	0.001	-1.732	0.017	-1.955	0.004
HALLMARK_E2F_TARGETS	-1.185	0.458	-2.775	0.000	-1.024	0.392	-2.832	0.000
HALLMARK_REACTIVE_OXYGEN_SPECIES_PATHWAY	-0.911	0.884	0.768	0.945	-1.309	0.143	-0.828	0.799
HALLMARK_OXIDATIVE_PHOSPHORYLATION	0.510	0.998	-0.779	1.000	-1.114	0.294	-1.681	0.023
HALLMARK_INFLAMMATORY_RESPONSE	2.196	0.000	1.512	0.108	1.894	0.000	1.895	0.000
HALLMARK_INTERFERON_GAMMA_RESPONSE	1.888	0.001	1.426	0.121	1.663	0.007	1.937	0.000
