# Supplementary material for: EZH2 Inhibition Promotes Tumor Immunogenicity in Lung Squamous Cell Carcinomas
Source: Cancer Res Commun. 2024 Feb 13;4(2):388–403. doi: 10.1158/2767-9764.CRC-23-0399 (PMC10863487; doi:10.1158/2767-9764.CRC-23-0399)
Supplement: Supplementary Figure 1 — shows changes in NGFR and CD274 (PD-L1) gene and protein expression in human lung cancer cell lines in response to EZH2 inhibitor and interferon-gamma treatment. [file crc-23-0399-s02.pdf]

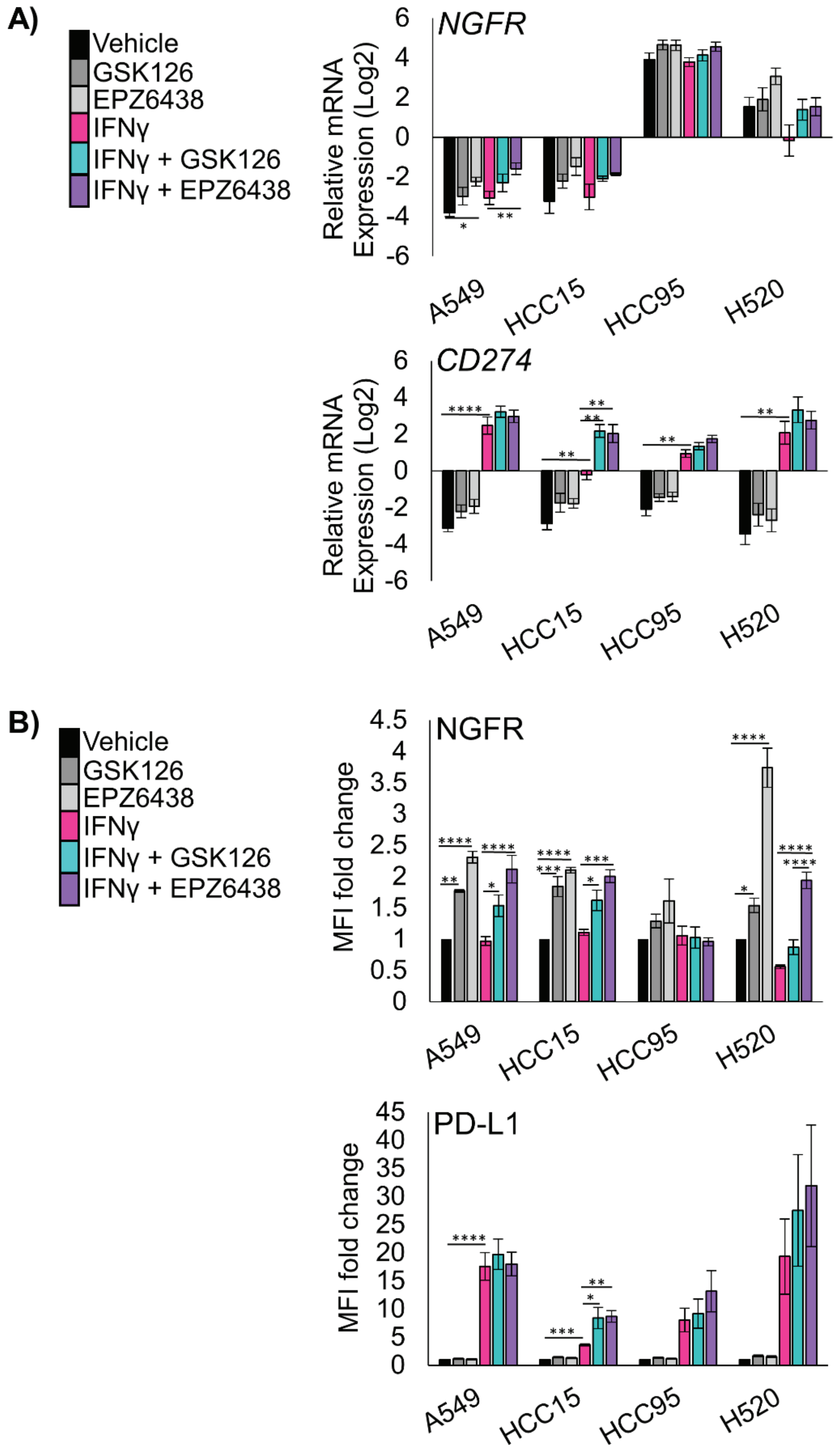

Supplementary Figure 1: Related to Figure 1

A) RT-qPCR in the indicated four human lung cancer cell lines treated for 7 days with vehicle or 5μM EZH2 inhibition with 20ng/mL IFNγ added on day 5 for the genes *NGFR* and *CD274*, mean +/- SEM is graphed, n = 4 individual experiments, * indicated p=0.0481 **p<0.0098, ****p<0.0001 by one-way ANOVA with pairwise comparisons and Holm-Šídák's *post hoc* test. **B)** Flow cytometry analysis of indicated four human lung cancer cell lines treated for 6 days with vehicle or 5μM EZH2 inhibition with 20ng/mL IFNγ added on day 5 for the cell

surface proteins NGFR and PD-L1, mean +/- SEM is graphed, n = 4 individual experiments, * indicated p = 0.04, **p=0.0033, ***p<0.0003, ****p<0.0001 by one-way ANOVA with pairwise comparisons and Holm-Šídák's *post hoc* test.
