# Supplementary material for: EZH2 Inhibition Promotes Tumor Immunogenicity in Lung Squamous Cell Carcinomas
Source: Cancer Res Commun. 2024 Feb 13;4(2):388–403. doi: 10.1158/2767-9764.CRC-23-0399 (PMC10863487; doi:10.1158/2767-9764.CRC-23-0399)
Supplement: Supplementary Table 2 — shows Gene Set Enrichment Analysis on genes with mRNA up-regulated and H3K27me3 peaks lost in combination treatment vs IFN-gamma alone in human lung squamous cell carcinoma tumoroids. [file crc-23-0399-s03.pdf]

Supplemental Table 2: GSEA on Genes That were Up-regulated and Lost H3K27me3 Peaks in Combination Treatment vs IFNγ alone, related to Figure 4

Group	MSigDB Signature Name	GeneRatio	BgRatio	P Value	Adj. P value	FDR q value
Inflammatory Responses	MANNE_COVID19_COMBINED_COHORT_VS_HEALTHY_DONOR_PLATELETS_DN	16/272	228/21697	3.32E-08	4.54878E-06	3.8288E-06
	MANNE_COVID19_ICU_VS_HEALTHY_DONOR_PLATELETS_DN	13/272	162/21697	1.4E-07	1.74026E-05	1.46481E-05
	REACTOME_INTERFERON_GAMMA_SIGNALING	8/272	93/21697	2.23E-05	0.001175233	0.000989219
	REACTOME_PD_1_SIGNALING	5/272	28/21697	2.32E-05	0.001175233	0.000989219
	REACTOME_MHC_CLASS_II_ANTIGEN_PRESENTATION	9/272	126/21697	3.07E-05	0.00150398	0.001265932
	KEGG_ASTHMA	5/272	30/21697	3.29E-05	0.001564249	0.001316661
	KEGG_ALLOGRAFT_REJECTION	5/272	37/21697	9.37E-05	0.003565296	0.003000985
	KEGG_GRAFT_VERSUS_HOST_DISEASE	5/272	41/21697	0.000155	0.005362662	0.004513866
Cell Adhesion and Signaling	KEGG_CELL_ADHESION_MOLECULES_CAMS	12/272	133/21697	1.18E-07	1.54175E-05	1.29773E-05
	ZWANG_TRANSIENTLY_UP_BY_2ND_EGF_PULSE_ONLY	50/272	1941/21697	7.24E-07	6.61582E-05	5.56867E-05
	ONDER_CDH1_TARGETS_2_UP	12/272	257/21697	0.000104	0.003918949	0.003298662
	KEGG_FOCAL_ADHESION	11/272	199/21697	4.51E-05	0.001992359	0.001677011
TP53 and Apoptosis	PEREZ_TP53_TARGETS	40/272	1198/21697	1.49E-08	2.39558E-06	2.01641E-06
	BRUINS_UVC_RESPONSE_VIA_TP53_GROUP_A	30/272	884/21697	7.99E-07	6.84384E-05	5.7606E-05
	PEREZ_TP53_AND_TP63_TARGETS	11/272	208/21697	6.73E-05	0.002792285	0.002350325
	HANN_RESISTANCE_TO_BCL2_INHIBITOR_UP	5/272	36/21697	8.19E-05	0.003298434	0.002776362
Nervous System	REACTOME_NEURONAL_SYSTEM	27/272	410/21697	2.36E-12	6.46166E-10	5.43892E-10
	REACTOME_TRANSMISSION_ACROSS_CHEMICAL_SYNAPSES	16/272	269/21697	3.22E-07	3.39241E-05	2.85546E-05
	REACTOME_NERVOUS_SYSTEM_DEVELOPMENT	23/272	580/21697	1.28E-06	0.000103351	8.69924E-05
	REACTOME_NEUROTRANSMITTER_RECEPTORS_AND_POSTSYNAPTIC_SIGNAL	10/272	205/21697	0.000276	0.008316188	0.006999911
Polycomb Targets	BENPORATH_ES_WITH_H3K27ME3	76/272	1114/21697	3.02E-35	8.26451E-32	6.95641E-32
	BENPORATH_SUZ12_TARGETS	70/272	1033/21697	4.01E-32	5.49216E-29	4.62287E-29
	MIKKELSEN_MEF_HCP_WITH_H3K27ME3	51/272	590/21697	4.28E-28	3.9059E-25	3.28768E-25
	BENPORATH_EED_TARGETS	64/272	1058/21697	1.75E-26	1.19586E-23	1.00658E-23
	MEISSNER_BRAIN_HCP_WITH_H3K4ME3_AND_H3K27ME3	64/272	1073/21697	3.81E-26	2.08627E-23	1.75606E-23
	MIKKELSEN_MCV6_HCP_WITH_H3K27ME3	43/272	437/21697	5.7E-26	2.60336E-23	2.1913E-23
	BENPORATH_PRC2_TARGETS	49/272	649/21697	2.32E-24	9.07209E-22	7.63617E-22
	MIKKELSEN_NPC_HCP_WITH_H3K27ME3	26/272	345/21697	2.83E-13	9.6866E-11	8.15342E-11
	MEISSNER_NPC_HCP_WITH_H3K4ME2_AND_H3K27ME3	26/272	350/21697	3.95E-13	1.20105E-10	1.01095E-10
	MEISSNER_BRAIN_HCP_WITH_H3K27ME3	20/272	271/21697	2.52E-10	6.27713E-08	5.28359E-08
	MIKKELSEN_NPC_HCP_WITH_H3K4ME3_AND_H3K27ME3	17/272	210/21697	1.42E-09	3.23121E-07	2.71978E-07
	MEISSNER_NPC_HCP_WITH_H3K4ME3_AND_H3K27ME3	13/272	143/21697	3.19E-08	4.54878E-06	3.8288E-06
