# Supplementary material for: EZH2 Inhibition Promotes Tumor Immunogenicity in Lung Squamous Cell Carcinomas
Source: Cancer Res Commun. 2024 Feb 13;4(2):388–403. doi: 10.1158/2767-9764.CRC-23-0399 (PMC10863487; doi:10.1158/2767-9764.CRC-23-0399)
Supplement: Supplementary Figure 2 — shows changes in NGFR and CD274 (PD-L1) gene and protein expression in human lung cancer tumoroids in response to EZH2 inhibitor and interferon-gamma treatment. [file crc-23-0399-s04.pdf]

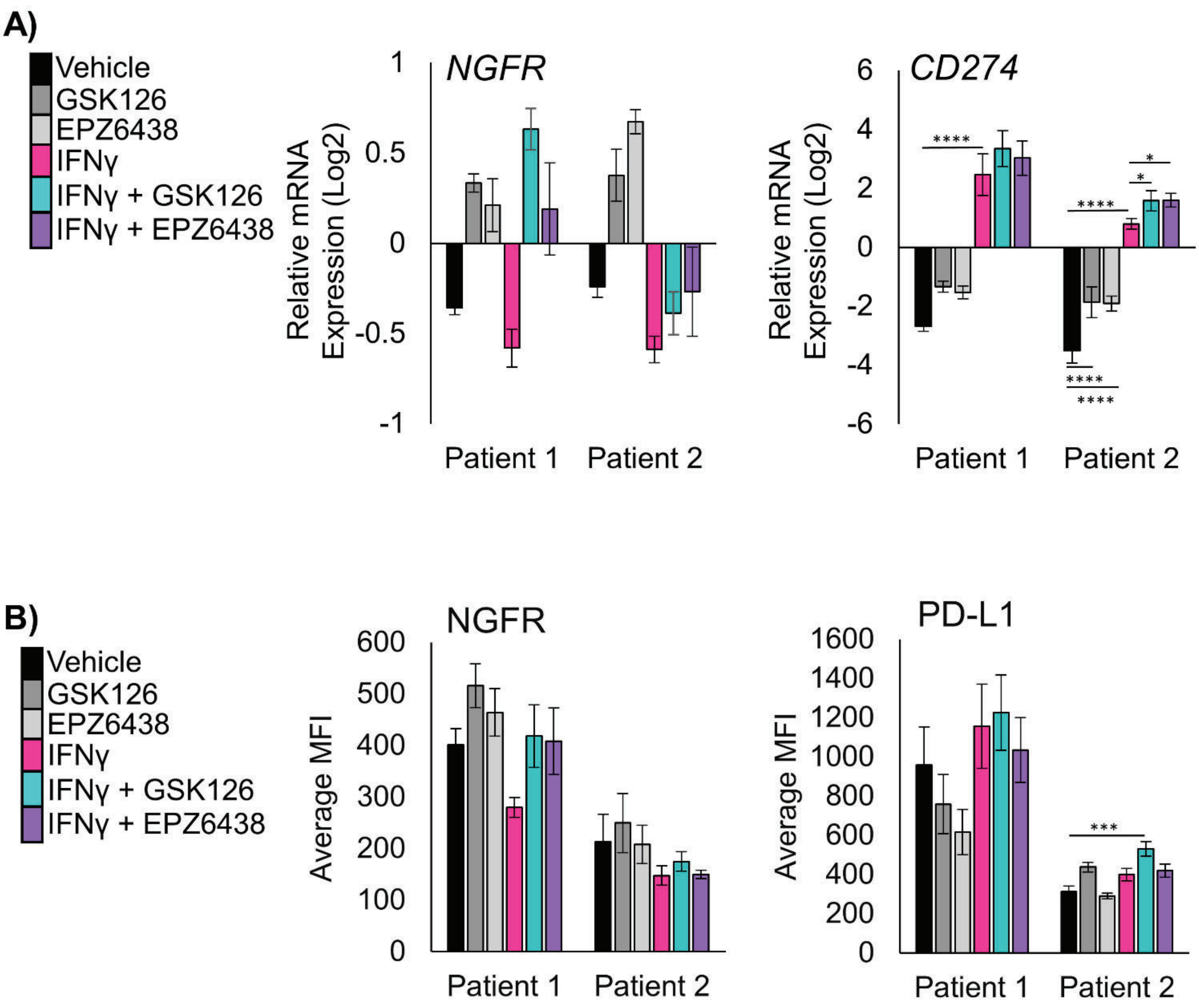

Supplementary Figure 2: Related to Figure 2

A) RT-qPCR in the indicated two unique patient-derived tumoroid cultures treated for 11 days with 5μM EZH2 inhibition with 20ng/mL IFNγ added on day 9 for the genes *NGFR* and *CD274*, mean +/- SEM is graphed, n = 4 individual experiments, * indicates p<0.03, ****p<0.0001 by one-way ANOVA with pairwise comparisons and Holm-Šídák's *post hoc* test. **B)** Flow cytometry analysis of indicated two unique patient-derived tumoroid cultures treated for 11 days with 5μM EZH2 inhibition with 20ng/mL IFNγ added on day 9 for the cell surface proteins NGFR and PD-L1, mean +/- SEM is graphed, n = 4 individual experiments, *** indicates p=0.0002 by one-way ANOVA with pairwise comparisons and Holm-Šídák's *post hoc* test.
