# Supplementary material for: EZH2 Inhibition Promotes Tumor Immunogenicity in Lung Squamous Cell Carcinomas
Source: Cancer Res Commun. 2024 Feb 13;4(2):388–403. doi: 10.1158/2767-9764.CRC-23-0399 (PMC10863487; doi:10.1158/2767-9764.CRC-23-0399)
Supplement: Supplementary Table 3 — shows gene highly expressed in each of the 16 clusters called in the single cell RNA-sequencing from murine lung and lung squamous cell carcinoma samples. [file crc-23-0399-s05.pdf]

Supplemental Table 3: Genes Highly Expressed in All 16 Clusters, Related to Figure 6
Log2FC=Log2-fold change between cluster and all others, pct=percentage of cells expressing

Cluster	Gene Symbol	Log2FC	pct.1	pct.2	P value	Adj P value	Cluster	Gene Symbol	Log2FC	pct.1	pct.2	P value	Adj P value
Tumor	Krt19	4.685	0.977	0.814	0.0E+00	0.0E+00	Dendritic	Cd74	4.390	0.964	0.555	0.0E+00	0.0E+00
	Ppbp	4.050	0.781	0.673	0.0E+00	0.0E+00		H2-Ab1	4.061	0.934	0.629	0.0E+00	0.0E+00
	S100a7a	3.910	0.908	0.872	0.0E+00	0.0E+00		C1qa	3.857	0.988	0.581	0.0E+00	0.0E+00
	S100a10	3.589	0.968	0.700	0.0E+00	0.0E+00		H2-Aa	3.618	0.918	0.556	0.0E+00	0.0E+00
	Ly6d	3.380	0.853	0.767	0.0E+00	0.0E+00		C1qb	3.455	0.982	0.503	0.0E+00	0.0E+00
	Prss27	3.341	0.899	0.825	0.0E+00	0.0E+00		Apoe	3.074	0.985	0.781	0.0E+00	0.0E+00
	Krt18	3.333	0.824	0.698	0.0E+00	0.0E+00		C1qc	2.985	0.988	0.640	0.0E+00	0.0E+00
	Plet1	3.301	0.954	0.684	0.0E+00	0.0E+00		Ms4a6c	2.243	0.930	0.407	0.0E+00	0.0E+00
	Nupr1	3.285	0.892	0.671	0.0E+00	0.0E+00		Ms4a7	2.238	0.969	0.433	0.0E+00	0.0E+00
	S100a16	3.221	0.951	0.730	0.0E+00	0.0E+00		Ctss	1.932	0.990	0.654	0.0E+00	0.0E+00
	Krt23	3.208	0.894	0.638	0.0E+00	0.0E+00		Cst3	1.844	0.987	0.817	0.0E+00	0.0E+00
	Ly6c1	2.943	0.832	0.652	0.0E+00	0.0E+00		Gatm	1.752	0.937	0.517	0.0E+00	0.0E+00
	Atp1b1	2.870	0.888	0.682	0.0E+00	0.0E+00		Lair1	1.530	0.895	0.546	0.0E+00	0.0E+00
	Irf7	2.800	0.937	0.588	0.0E+00	0.0E+00		Pld4	1.525	0.899	0.187	0.0E+00	0.0E+00
	Isg15	2.772	0.869	0.644	0.0E+00	0.0E+00		Tmem119	0.828	0.959	0.256	0.0E+00	0.0E+00
	Tspan1	2.562	0.857	0.516	0.0E+00	0.0E+00		Cxcl9	0.789	0.946	0.644	0.0E+00	0.0E+00
	Hspa5	2.539	0.951	0.818	0.0E+00	0.0E+00		Ccl8	0.651	0.962	0.382	0.0E+00	0.0E+00
	Glrx	2.448	0.869	0.594	0.0E+00	0.0E+00		Retnla	0.821	0.957	0.743	2.9E-289	4.4E-286
	Emp1	2.302	0.920	0.778	0.0E+00	0.0E+00		Cybb	1.677	0.924	0.514	1.9E-288	2.9E-285
	Hopx	2.294	0.766	0.477	0.0E+00	0.0E+00		Gngt2	1.535	0.964	0.637	4.8E-284	7.2E-281
	Cd81	2.276	0.909	0.547	0.0E+00	0.0E+00		Cxcl16	1.511	0.983	0.701	5.4E-279	8.1E-276
	Arg1	2.255	0.872	0.739	0.0E+00	0.0E+00		Fyb	1.709	0.949	0.688	1.7E-277	2.5E-274
	Scgb1a1	2.246	0.831	0.809	0.0E+00	0.0E+00		Pou2f2	1.956	0.849	0.421	1.4E-270	2.1E-267
	Lmo7	2.226	0.849	0.750	0.0E+00	0.0E+00		Acp5	1.404	0.968	0.607	4.2E-268	6.3E-265
	Ceacam1	2.214	0.888	0.649	0.0E+00	0.0E+00		Lgmn	1.372	0.964	0.624	2.1E-257	3.2E-254
	Dusp14	2.072	0.836	0.675	0.0E+00	0.0E+00		Aif1	2.087	0.853	0.573	2.8E-255	4.3E-252
	Wnt4	2.037	0.882	0.722	0.0E+00	0.0E+00		Atp1a1	1.325	0.957	0.700	3.7E-254	5.6E-251
	Clu	2.031	0.918	0.761	0.0E+00	0.0E+00		Lyz2	0.748	0.994	0.755	9.3E-252	1.4E-248
	Epcam	2.006	0.848	0.601	0.0E+00	0.0E+00		Trf	1.377	0.891	0.499	4.4E-247	6.7E-244
	Anxa1	2.002	0.919	0.772	0.0E+00	0.0E+00		Ms4a6d	1.230	0.895	0.733	2.9E-240	4.4E-237
	Cldn3	1.963	0.803	0.773	0.0E+00	0.0E+00		Csf1r	1.731	0.824	0.437	2.0E-233	3.1E-230
	2310007B03Ril	1.887	0.861	0.663	0.0E+00	0.0E+00		Bcl2a1d	1.057	0.884	0.505	3.6E-223	5.4E-220
	Pdzk1ip1	1.882	0.850	0.786	0.0E+00	0.0E+00		Fcgr1	0.866	0.839	0.480	3.3E-217	5.0E-214
	Prss22	1.850	0.702	0.557	0.0E+00	0.0E+00		Napsa	1.159	0.904	0.579	1.7E-213	2.5E-210
	Aqp5	1.785	0.676	0.741	0.0E+00	0.0E+00		Ly86	1.085	0.807	0.287	7.3E-213	1.1E-209
	Cldn7	1.777	0.732	0.679	0.0E+00	0.0E+00		Ctsc	1.394	0.881	0.644	2.9E-207	4.4E-204
	Jdp2	1.736	0.810	0.711	0.0E+00	0.0E+00		Gm2a	1.234	0.909	0.624	5.8E-207	8.7E-204
	Plpp1	1.699	0.783	0.653	0.0E+00	0.0E+00		Mafb	1.508	0.853	0.588	4.2E-199	6.3E-196
	Avpi1	1.689	0.843	0.712	0.0E+00	0.0E+00		Ccr2	0.905	0.788	0.255	3.8E-195	5.7E-192
	Nccrp1	1.658	0.754	0.730	0.0E+00	0.0E+00		Ifitm3	1.495	0.943	0.695	1.8E-194	2.6E-191
	Errfi1	1.618	0.894	0.564	0.0E+00	0.0E+00		Pgls	0.758	0.921	0.629	5.1E-182	7.6E-179
	Plat	1.601	0.627	0.504	0.0E+00	0.0E+00		Itgb5	0.923	0.849	0.676	1.1E-177	1.6E-174
	Gsta4	1.599	0.796	0.689	0.0E+00	0.0E+00		Rpl3	1.055	0.970	0.708	1.1E-177	1.7E-174
	Tacstd2	1.567	0.736	0.538	0.0E+00	0.0E+00		Gpx1	0.883	0.965	0.833	4.2E-173	6.3E-170
	Calr	1.558	0.945	0.734	0.0E+00	0.0E+00		Clec4a3	1.528	0.770	0.440	5.1E-172	7.6E-169
	Tubb4b	1.556	0.918	0.674	0.0E+00	0.0E+00		Cd72	1.163	0.717	0.163	1.4E-171	2.1E-168
	Wwtr1	1.537	0.890	0.574	0.0E+00	0.0E+00		Cx3cr1	1.589	0.759	0.360	7.3E-171	1.1E-167
	Fxyd3	1.529	0.782	0.582	0.0E+00	0.0E+00		Tgfbi	0.964	0.915	0.601	2.9E-170	4.4E-167
	Csrp1	1.520	0.819	0.671	0.0E+00	0.0E+00		Asah1	0.779	0.879	0.564	1.0E-169	1.5E-166
	Spns3	1.512	0.823	0.552	0.0E+00	0.0E+00		Ptma	0.975	0.965	0.726	1.6E-165	2.4E-162
	Mall	1.506	0.859	0.721	0.0E+00	0.0E+00		Smpdl3a	1.034	0.904	0.622	3.0E-163	4.5E-160
	Gprc5a	1.498	0.799	0.586	0.0E+00	0.0E+00		Ucp2	0.852	0.973	0.726	5.8E-162	8.7E-159
	Herpud1	1.429	0.790	0.560	0.0E+00	0.0E+00		Bcl2a1a	0.700	0.859	0.545	1.3E-159	1.9E-156
	Ddit3	1.405	0.820	0.723	0.0E+00	0.0E+00		Cd300e	1.156	0.727	0.244	4.8E-158	7.1E-155
	Clec2g	1.386	0.818	0.436	0.0E+00	0.0E+00		Ctsz	0.849	0.994	0.790	2.0E-152	3.1E-149
	Dnajb1	1.374	0.893	0.567	0.0E+00	0.0E+00		Laptm5	0.768	0.989	0.732	2.3E-151	3.5E-148
	Cidea	1.304	0.610	0.536	0.0E+00	0.0E+00		Rassf4	0.995	0.789	0.576	1.5E-149	2.3E-146
	Lrrc26	1.302	0.812	0.724	0.0E+00	0.0E+00		Emp3	1.135	0.840	0.456	8.3E-148	1.2E-144
	Cldn23	1.294	0.734	0.615	0.0E+00	0.0E+00		Erp29	1.067	0.922	0.709	9.2E-146	1.4E-142
	Chac1	1.291	0.750	0.706	0.0E+00	0.0E+00		Sdf2l1	0.667	0.902	0.718	1.0E-143	1.5E-140
	Rdh10	1.290	0.787	0.518	0.0E+00	0.0E+00		Cd300c2	0.691	0.932	0.651	5.4E-142	8.1E-139
	Fam57a	1.279	0.733	0.754	0.0E+00	0.0E+00		Clec4a1	1.686	0.720	0.281	1.3E-141	1.9E-138
	Gadd45b	1.263	0.948	0.711	0.0E+00	0.0E+00		Ms4a6b	1.104	0.755	0.271	1.1E-138	1.6E-135
	Cops6	1.253	0.833	0.647	0.0E+00	0.0E+00		Tmem160	0.781	0.834	0.577	2.9E-137	4.4E-134
	Krt8	1.231	0.690	0.700	0.0E+00	0.0E+00		H2-DMa	1.252	0.756	0.394	3.1E-136	4.7E-133
	Gadd45g	1.225	0.659	0.573	0.0E+00	0.0E+00		Abhd12	0.845	0.790	0.432	6.7E-135	1.0E-131
	Fam110a	1.163	0.787	0.499	0.0E+00	0.0E+00		Hexb	1.403	0.863	0.751	6.5E-132	9.7E-129
	Pls3	1.149	0.784	0.594	0.0E+00	0.0E+00		S100a4	1.042	0.790	0.500	1.6E-130	2.3E-127
	Hbegf	1.114	0.731	0.762	0.0E+00	0.0E+00		Flna	0.830	0.835	0.487	2.2E-128	3.3E-125

	Phlda1	1.100	0.759	0.510	0.0E+00	0.0E+00
	Krt80	1.097	0.845	0.529	0.0E+00	0.0E+00
	Manf	1.096	0.869	0.829	0.0E+00	0.0E+00
	Parm1	1.078	0.778	0.721	0.0E+00	0.0E+00
	Hsd17b2	1.076	0.686	0.537	0.0E+00	0.0E+00
	Slco2a1	1.075	0.676	0.528	0.0E+00	0.0E+00
	2610528A11Ril	1.070	0.701	0.641	0.0E+00	0.0E+00
	Apol7a	1.050	0.698	0.453	0.0E+00	0.0E+00
	Mlph	1.045	0.754	0.665	0.0E+00	0.0E+00
	Sqstm1	1.023	0.873	0.581	0.0E+00	0.0E+00
	Pmaip1	0.991	0.742	0.669	0.0E+00	0.0E+00
	Oasl1	0.984	0.620	0.575	0.0E+00	0.0E+00
	Plac8	0.977	0.735	0.523	0.0E+00	0.0E+00
	Ifi202b	0.963	0.703	0.648	0.0E+00	0.0E+00
	Rbp1	0.960	0.608	0.545	0.0E+00	0.0E+00
	2010109I03Rik	0.930	0.607	0.662	0.0E+00	0.0E+00
	Tspan8	0.903	0.766	0.646	0.0E+00	0.0E+00
	Yod1	0.898	0.649	0.569	0.0E+00	0.0E+00
	Cald1	0.888	0.677	0.690	0.0E+00	0.0E+00
	Plk2	0.881	0.723	0.493	0.0E+00	0.0E+00
	Ephb3	0.877	0.717	0.631	0.0E+00	0.0E+00
	Oit1	0.851	0.751	0.566	0.0E+00	0.0E+00
	Hsp90aa1	0.846	0.911	0.832	0.0E+00	0.0E+00
	Klk8	0.932	0.547	0.488	6.8E-289	1.0E-285
	Hsp90b1	1.266	0.827	0.781	2.1E-272	3.1E-269
	Tsc22d1	0.850	0.640	0.773	8.6E-178	1.3E-174
	Clic5	1.114	0.581	0.733	7.7E-159	1.2E-155
	Ifit1bl1	1.446	0.487	0.470	6.4E-149	9.6E-146
	Cxcl5	1.588	0.446	0.399	6.7E-125	1.0E-121
	Pigr	0.995	0.441	0.469	6.3E-68	9.5E-65
	Cxcl3	1.035	0.364	0.517	5.7E-03	1.0E+00
**Neu-1**	Cst3	1.532	0.929	0.800	0.0E+00	0.0E+00
	Gngt2	1.374	0.812	0.614	0.0E+00	0.0E+00
	Id2	1.172	0.925	0.753	0.0E+00	0.0E+00
	Ptgs1	1.172	0.772	0.678	0.0E+00	0.0E+00
	Laptm5	0.838	0.829	0.722	0.0E+00	0.0E+00
	Gm19951	0.675	0.840	0.656	0.0E+00	0.0E+00
	Ccl4	0.552	0.895	0.685	0.0E+00	0.0E+00
	Hist1h1e	0.491	0.794	0.590	0.0E+00	0.0E+00
	Asprv1	0.446	0.778	0.552	0.0E+00	0.0E+00
	Egr2	0.310	0.274	0.401	3.3E-290	5.0E-287
	B930036N10Ri	0.499	0.642	0.468	1.2E-283	1.8E-280
	Id1	1.043	0.832	0.665	4.7E-266	7.1E-263
	Gpx1	0.848	0.869	0.830	3.8E-265	5.7E-262
	Ms4a6d	0.565	0.805	0.725	7.4E-254	1.1E-250
	Csf1r	0.366	0.280	0.483	9.3E-226	1.4E-222
	Gpc3	0.286	0.688	0.670	3.7E-198	5.5E-195
	Cd300c2	1.210	0.716	0.649	5.2E-190	7.8E-187
	Hist1h1d	0.253	0.697	0.533	8.4E-188	1.3E-184
	Ptgs2	0.526	0.234	0.306	4.4E-180	6.6E-177
	Pmaip1	0.518	0.787	0.662	1.1E-178	1.6E-175
	Ccl3	0.592	0.738	0.624	1.2E-148	1.8E-145
	Osgin1	0.724	0.685	0.570	7.6E-146	1.1E-142
	Olr1	0.348	0.328	0.404	8.3E-139	1.2E-135
	Pi16	0.312	0.619	0.443	4.3E-134	6.5E-131
	Hexb	0.810	0.794	0.746	4.2E-133	6.3E-130
	Fcgr4	0.354	0.750	0.685	1.7E-126	2.6E-123
	Bcl2a1d	0.547	0.380	0.543	1.6E-123	2.4E-120
	Cd63	0.327	0.866	0.768	2.9E-120	4.4E-117
	Ier3	0.771	0.802	0.786	5.3E-113	8.0E-110
	Atp1a1	0.853	0.779	0.694	1.1E-110	1.7E-107
	Hk2	0.263	0.470	0.609	5.4E-104	8.0E-101
	P2ry6	0.401	0.710	0.682	1.5E-100	2.3E-97
	Lrg1	0.265	0.765	0.686	1.4E-95	2.1E-92
	Gm12840	1.099	0.321	0.411	2.0E-95	3.0E-92
	H1f0	0.599	0.700	0.568	2.2E-87	3.3E-84
	Naaa	0.359	0.655	0.568	4.8E-75	7.2E-72
	Ltc4s	0.796	0.644	0.599	1.2E-72	1.9E-69
	Reep5	0.646	0.749	0.711	5.2E-55	7.9E-52
	Egr1	0.842	0.617	0.544	1.1E-51	1.6E-48
	Asah1	0.261	0.666	0.555	1.3E-49	2.0E-46
	Gm2a	0.855	0.637	0.632	1.0E-45	1.5E-42
	Hist1h4i	1.255	0.646	0.671	1.8E-45	2.8E-42
	Chil1	0.513	0.593	0.475	2.1E-43	3.2E-40
	Tgm2	0.664	0.463	0.641	5.8E-43	8.6E-40

	Unc93b1	0.755	0.848	0.511	1.4E-127	2.0E-124
	Pid1	0.802	0.765	0.585	1.5E-127	2.2E-124
	Rras	0.921	0.760	0.544	7.2E-126	1.1E-122
	Itgb2	0.950	0.910	0.585	2.3E-124	3.5E-121
	Lilra5	1.100	0.724	0.321	2.1E-120	3.2E-117
	Mgst1	0.710	0.874	0.601	1.1E-119	1.6E-116
	Selenop	0.924	0.776	0.519	2.1E-115	3.2E-112
	mt-Nd1	0.722	0.963	0.666	1.7E-114	2.6E-111
	Fcgrt	0.886	0.735	0.282	7.0E-114	1.0E-110
	Plekho1	0.926	0.756	0.553	7.7E-112	1.2E-108
	Ndufa4	0.708	0.879	0.666	2.0E-111	3.0E-108
	Mef2c	1.025	0.724	0.384	3.3E-106	5.0E-103
	Irf8	1.231	0.760	0.635	2.2E-105	3.3E-102
	Npc2	0.824	0.958	0.889	2.4E-101	3.7E-98
	Plac8	1.352	0.759	0.557	6.2E-100	9.3E-97
	H2-DMb1	1.111	0.707	0.428	3.2E-94	4.9E-91
	Tifab	0.751	0.686	0.354	2.7E-81	4.1E-78
	Pltp	0.669	0.703	0.365	9.6E-81	1.4E-77
	Ccdc88a	1.007	0.688	0.547	2.3E-70	3.5E-67
	Ear2	1.003	0.647	0.320	8.7E-66	1.3E-62
	Ms4a4c	0.756	0.630	0.135	1.7E-64	2.5E-61
	Ace	1.683	0.644	0.536	6.6E-64	9.9E-61
	Evl	0.693	0.688	0.340	1.6E-61	2.4E-58
	Ifi27l2a	0.784	0.697	0.528	8.9E-55	1.3E-51
	Gm15987	0.810	0.635	0.261	1.1E-54	1.6E-51
	Klf4	0.724	0.648	0.422	4.9E-44	7.3E-41
	Slc12a2	1.262	0.706	0.578	4.8E-43	7.2E-40
	Adgre4	0.930	0.597	0.375	2.9E-42	4.4E-39
	Bst2	0.727	0.651	0.506	1.1E-30	1.7E-27
	Tmem176b	1.272	0.622	0.568	1.4E-22	2.1E-19
	Krt80	0.745	0.634	0.587	1.1E-20	1.6E-17
**B cells**	Igkc	4.317	0.964	0.279	0.0E+00	0.0E+00
	Ighm	3.141	0.908	0.317	0.0E+00	0.0E+00
	Cd74	3.059	0.992	0.554	0.0E+00	0.0E+00
	H2-Ab1	2.828	0.975	0.628	0.0E+00	0.0E+00
	H2-Aa	2.789	0.983	0.554	0.0E+00	0.0E+00
	Cd79a	2.550	0.908	0.408	0.0E+00	0.0E+00
	Cd79b	2.207	0.854	0.309	0.0E+00	0.0E+00
	Ebf1	2.190	0.862	0.157	0.0E+00	0.0E+00
	Iglc2	2.133	0.870	0.147	0.0E+00	0.0E+00
	Cd72	1.409	0.846	0.159	0.0E+00	0.0E+00
	Serpina3g	0.465	0.972	0.560	0.0E+00	0.0E+00
	Gatm	0.355	0.992	0.516	0.0E+00	0.0E+00
	Ighd	2.304	0.799	0.270	1.4E-299	2.1E-296
	Gm15987	0.802	0.838	0.255	1.2E-273	1.9E-270
	Jchain	1.341	0.826	0.370	9.8E-271	1.5E-267
	Ms4a1	1.914	0.819	0.197	6.8E-270	1.0E-266
	Ly6d	0.446	0.929	0.779	5.9E-245	8.9E-242
	Rpl3	1.155	0.991	0.707	7.0E-233	1.1E-229
	Mef2c	1.562	0.830	0.381	8.7E-231	1.3E-227
	Bank1	1.757	0.792	0.348	2.6E-210	3.9E-207
	Ptma	0.989	0.991	0.725	2.2E-205	3.3E-202
	Igha	1.645	0.780	0.378	1.0E-200	1.5E-197
	Ighg1	1.025	0.772	0.364	8.7E-190	1.3E-186
	H2-DMa	0.962	0.810	0.392	7.6E-178	1.1E-174
	Fam96a	0.433	0.939	0.556	5.6E-177	8.5E-174
	Gpr65	0.440	0.838	0.448	3.8E-174	5.7E-171
	Iglc3	1.588	0.712	0.157	2.9E-168	4.3E-165
	Napsa	1.338	0.843	0.582	3.3E-163	5.0E-160
	mt-Nd1	0.665	0.967	0.666	1.3E-152	1.9E-149
	Ets1	1.116	0.792	0.447	1.3E-150	1.9E-147
	Mzb1	1.617	0.728	0.330	2.9E-146	4.3E-143
	Rps2	0.667	0.989	0.892	8.8E-135	1.3E-131
	Fcmr	1.153	0.750	0.392	8.3E-132	1.2E-128
	Gm31243	1.017	0.709	0.181	9.7E-121	1.5E-117
	H2-DMb2	1.138	0.718	0.272	2.2E-119	3.3E-116
	Ranbp1	0.695	0.826	0.642	4.2E-105	6.4E-102
	Gm8369	0.449	0.297	0.105	1.4E-101	2.1E-98
	Gimap6	1.256	0.710	0.321	1.5E-98	2.3E-95
	4930523C07R	1.050	0.716	0.510	6.0E-92	9.0E-89
	Ptprcap	0.932	0.716	0.524	7.3E-82	1.1E-78
	Cd55	0.849	0.734	0.433	3.2E-81	4.8E-78
	Fcer2a	1.434	0.669	0.501	4.6E-75	6.9E-72
	Erp29	0.518	0.910	0.710	1.4E-73	2.1E-70
	Pax5	0.985	0.641	0.212	3.4E-73	5.1E-70

	Dpep2	0.374	0.652	0.687	7.8E-43	1.2E-39
	Ctsa	0.381	0.744	0.697	2.0E-42	2.9E-39
	Fgd4	0.251	0.688	0.624	1.5E-41	2.3E-38
	Fam20c	0.442	0.792	0.763	5.8E-41	8.7E-38
	Pou2f2	0.312	0.387	0.444	1.9E-34	2.9E-31
	Cks2	1.189	0.534	0.460	3.2E-33	4.8E-30
	Rhoc	0.380	0.529	0.605	3.6E-33	5.3E-30
	Fyb	0.514	0.751	0.686	7.6E-32	1.1E-28
	Fcgr2b	0.347	0.713	0.648	2.8E-31	4.1E-28
	C3	0.875	0.585	0.551	5.4E-30	8.1E-27
	Slc7a11	0.272	0.428	0.478	6.5E-28	9.7E-25
	Hist1h1c	0.763	0.664	0.606	5.7E-26	8.5E-23
	Tcirg1	0.280	0.550	0.636	9.7E-26	1.5E-22
	Abca1	0.438	0.768	0.731	1.2E-25	1.7E-22
	Rgs1	1.284	0.650	0.628	1.6E-25	2.4E-22
	Csf2rb	0.693	0.676	0.689	3.6E-25	5.5E-22
	Bcl2a1b	0.872	0.640	0.631	2.0E-23	2.9E-20
	Ccng1	0.266	0.520	0.595	7.3E-23	1.1E-19
	Agap1	0.867	0.586	0.610	2.6E-22	3.9E-19
	Erp29	0.301	0.600	0.739	1.2E-21	1.7E-18
	Slc6a6	0.338	0.463	0.522	2.5E-21	3.7E-18
	Tnf	0.725	0.327	0.308	9.4E-19	1.4E-15
	Hist1h2ap	0.263	0.508	0.479	6.2E-18	9.3E-15
	Gm20186	0.564	0.404	0.440	6.6E-18	9.9E-15
	Cfp	0.354	0.543	0.434	1.4E-17	2.2E-14
	Unc93b1	0.643	0.570	0.512	3.2E-16	4.8E-13
	Ptma	0.282	0.760	0.728	3.5E-15	5.2E-12
	Itgax	0.746	0.468	0.563	2.5E-14	3.8E-11
	Aprt	0.365	0.639	0.761	2.1E-12	3.1E-09
	Itgb2	0.303	0.503	0.614	2.2E-12	3.3E-09
	Ucp2	0.280	0.743	0.732	4.5E-11	6.8E-08
	Bcl2a1a	0.956	0.592	0.547	7.5E-10	1.1E-06
	Arhgap25	0.475	0.546	0.497	4.7E-08	7.1E-05
	G0s2	0.960	0.524	0.494	1.9E-06	2.8E-03
	Gm26870	0.339	0.577	0.569	5.6E-05	8.3E-02
	Cdkn1a	0.436	0.825	0.760	5.5E-04	8.2E-01
	Tgfbi	0.656	0.586	0.616	5.7E-04	8.5E-01
	Mpeg1	0.405	0.595	0.629	6.4E-04	9.6E-01
	Csf2ra	0.674	0.543	0.575	1.5E-03	1.0E+00
	Tnfrsf23	0.465	0.654	0.712	5.8E-03	1.0E+00
**Neu-2**	Gm5483	2.762	0.938	0.564	0.0E+00	0.0E+00
	BC100530	2.671	0.815	0.520	0.0E+00	0.0E+00
	Wfdc17	2.485	0.993	0.744	0.0E+00	0.0E+00
	Cxcl2	2.340	0.951	0.469	0.0E+00	0.0E+00
	Ifitm1	2.087	0.963	0.701	0.0E+00	0.0E+00
	Retnlg	2.057	0.960	0.576	0.0E+00	0.0E+00
	Wfdc21	1.768	0.945	0.605	0.0E+00	0.0E+00
	Lrg1	1.760	0.929	0.661	0.0E+00	0.0E+00
	G0s2	1.756	0.819	0.446	0.0E+00	0.0E+00
	Egr1	1.705	0.912	0.497	0.0E+00	0.0E+00
	Lcn2	1.667	0.911	0.561	0.0E+00	0.0E+00
	Ier3	1.643	0.972	0.758	0.0E+00	0.0E+00
	Slpi	1.397	0.904	0.602	0.0E+00	0.0E+00
	Adam8	1.286	0.729	0.572	0.0E+00	0.0E+00
	Ccl4	1.276	0.981	0.676	0.0E+00	0.0E+00
	F630028O10Ri	1.234	0.798	0.503	0.0E+00	0.0E+00
	Slfn4	1.160	0.942	0.505	0.0E+00	0.0E+00
	Id1	0.989	0.915	0.655	0.0E+00	0.0E+00
	Csf2rb	0.976	0.921	0.648	0.0E+00	0.0E+00
	Steap4	0.965	0.732	0.447	0.0E+00	0.0E+00
	Hcar2	0.955	0.882	0.648	0.0E+00	0.0E+00
	Prok2	0.868	0.790	0.548	0.0E+00	0.0E+00
	Olfm4	0.701	0.793	0.426	0.0E+00	0.0E+00
	Dgat2	0.563	0.795	0.603	0.0E+00	0.0E+00
	Tceal9	0.557	0.888	0.641	0.0E+00	0.0E+00
	Cxcl3	0.533	0.840	0.429	0.0E+00	0.0E+00
	Cd177	0.461	0.740	0.458	0.0E+00	0.0E+00
	Tnfrsf23	0.440	0.910	0.668	0.0E+00	0.0E+00
	Hk2	0.430	0.884	0.536	0.0E+00	0.0E+00
	Ccl6	0.398	0.910	0.592	0.0E+00	0.0E+00
	Cdkn1a	0.298	0.945	0.742	0.0E+00	0.0E+00
	Rgcc	0.260	0.926	0.710	0.0E+00	0.0E+00
	Slc7a11	0.868	0.715	0.429	1.1E-307	1.6E-304
	Mmp8	0.729	0.731	0.597	8.0E-292	1.2E-288
	Stfa2l1	2.238	0.682	0.448	1.4E-272	2.1E-269
	Ndufa4	0.509	0.843	0.667	4.6E-69	6.9E-66
	Sdc4	0.893	0.745	0.674	2.2E-67	3.3E-64
	Tnfrsf13c	0.888	0.622	0.177	2.7E-67	4.1E-64
	Scd1	1.063	0.661	0.348	8.6E-65	1.3E-61
	Hmgn1	0.833	0.723	0.652	7.2E-61	1.1E-57
	Hspe1	0.691	0.777	0.674	5.8E-60	8.7E-57
	Hes1	0.392	0.338	0.532	7.1E-59	1.1E-55
	Pxdc1	0.899	0.660	0.339	2.1E-58	3.2E-55
	Cd180	0.448	0.352	0.326	1.2E-54	1.8E-51
	Pgls	0.647	0.753	0.634	2.2E-54	3.3E-51
	Nap1l1	0.555	0.726	0.561	1.5E-44	2.2E-41
	Blk	0.482	0.380	0.328	1.6E-43	2.3E-40
	Bcar3	0.432	0.387	0.408	3.1E-43	4.7E-40
	Gimap3	0.924	0.632	0.218	1.3E-42	2.0E-39
	AC149090.1	1.234	0.659	0.455	1.3E-41	1.9E-38
	Irf8	0.826	0.671	0.638	8.6E-38	1.3E-34
	Pou2f2	0.590	0.656	0.428	5.8E-36	8.7E-33
	Ms4a4c	0.498	0.386	0.143	8.2E-36	1.2E-32
	Tubb5	0.433	0.727	0.599	1.1E-33	1.7E-30
	Ly86	0.735	0.619	0.293	8.3E-27	1.2E-23
	Aldh2	0.584	0.639	0.517	2.6E-26	3.9E-23
	S1pr1	0.415	0.391	0.514	1.3E-24	1.9E-21
	Id3	0.855	0.605	0.569	1.8E-24	2.7E-21
	Pou2af1	1.186	0.585	0.245	5.4E-24	8.1E-21
	Lmo2	0.870	0.616	0.400	1.0E-23	1.5E-20
	Gimap1	0.903	0.615	0.455	3.2E-23	4.9E-20
	C1qbp	0.416	0.684	0.663	3.0E-22	4.5E-19
	Slc25a4	0.458	0.662	0.537	2.2E-21	3.3E-18
	Gimap7	0.681	0.581	0.231	4.9E-21	7.3E-18
	Myo1e	0.370	0.454	0.610	3.7E-20	5.5E-17
	Siglecg	0.415	0.590	0.505	4.4E-17	6.6E-14
	Eprs	0.470	0.654	0.584	2.2E-16	3.3E-13
	Nucks1	0.526	0.636	0.534	2.7E-15	4.0E-12
	Plekho1	0.670	0.621	0.557	1.2E-14	1.9E-11
	Zbtb20	0.422	0.454	0.546	7.7E-14	1.2E-10
	Evl	0.561	0.600	0.343	3.7E-13	5.6E-10
	Snx5	0.608	0.621	0.551	2.3E-12	3.5E-09
	Ciita	0.638	0.441	0.281	1.7E-11	2.6E-08
	Blnk	0.663	0.596	0.738	2.1E-10	3.2E-07
	Sptbn1	0.654	0.623	0.580	6.2E-10	9.3E-07
	Iglc1	1.620	0.546	0.204	9.9E-09	1.5E-05
	Ms4a6c	0.618	0.593	0.418	1.4E-08	2.1E-05
	Cd2ap	0.534	0.624	0.597	1.7E-08	2.6E-05
	Cd69	0.605	0.558	0.334	5.9E-08	8.8E-05
	Ms4a6b	0.387	0.469	0.280	2.3E-07	3.4E-04
	Bin1	0.731	0.578	0.566	2.8E-07	4.2E-04
	Kcnq1ot1	0.583	0.624	0.657	3.6E-07	5.4E-04
	Odc1	0.433	0.478	0.572	3.6E-07	5.4E-04
	Vpreb3	1.232	0.539	0.135	4.7E-07	7.1E-04
	Nr4a1	0.505	0.424	0.317	1.1E-06	1.7E-03
	Rftn1	0.435	0.495	0.627	2.7E-06	4.1E-03
	Pdia4	0.591	0.566	0.537	7.0E-06	1.1E-02
	Gpr171	0.531	0.481	0.578	3.9E-05	5.9E-02
	Ikzf3	0.915	0.544	0.267	4.1E-05	6.2E-02
	Gpr183	0.771	0.535	0.267	4.2E-04	6.2E-01
	Cd38	0.484	0.588	0.584	8.6E-03	1.0E+00
**Lung Ep.**	Il33	1.978	0.892	0.537	0.0E+00	0.0E+00
	Areg	1.182	0.853	0.425	1.7E-291	2.6E-288
	Fxyd3	2.925	0.930	0.612	1.3E-275	2.0E-272
	Wfdc2	4.063	0.911	0.760	1.7E-275	2.5E-272
	Col1a1	1.424	0.721	0.220	4.9E-249	7.3E-246
	Dapl1	1.474	0.823	0.391	1.1E-243	1.6E-240
	Col1a2	1.973	0.756	0.381	8.7E-239	1.3E-235
	Btbd3	1.083	0.834	0.570	1.3E-220	1.9E-217
	Ly6d	1.756	0.948	0.779	8.8E-220	1.3E-216
	Hspb1	1.934	0.823	0.582	6.5E-217	9.7E-214
	Wfdc3	1.449	0.821	0.682	5.9E-205	8.8E-202
	Ckmt1	1.370	0.883	0.732	1.1E-200	1.7E-197
	Gpx2	1.928	0.819	0.571	1.7E-198	2.6E-195
	Cdh1	1.240	0.859	0.604	1.2E-197	1.8E-194
	Tmem176a	2.040	0.873	0.582	7.6E-197	1.1E-193
	Ehf	2.134	0.855	0.799	5.1E-196	7.6E-193
	Gsta4	2.610	0.871	0.705	1.8E-191	2.7E-188
	Them5	1.260	0.732	0.268	1.7E-187	2.5E-184
	Cldn7	1.072	0.896	0.684	1.1E-182	1.6E-179

	Osgin1	0.516	0.765	0.560	5.6E-269	8.5E-266
	Stfa2	1.396	0.680	0.497	3.6E-261	5.4E-258
	Il1f9	0.937	0.718	0.534	1.0E-248	1.6E-245
	Gadd45a	0.936	0.731	0.606	1.1E-241	1.7E-238
	Ifitm3	0.397	0.844	0.679	2.8E-228	4.3E-225
	Hip1	0.280	0.812	0.586	5.1E-221	7.7E-218
	Ifitm6	0.479	0.683	0.435	1.5E-216	2.2E-213
	Chil1	0.781	0.691	0.462	2.0E-216	2.9E-213
	Asprv1	1.206	0.707	0.570	1.3E-199	1.9E-196
	Tgm2	0.428	0.758	0.587	5.2E-159	7.8E-156
	Acod1	0.849	0.624	0.389	1.6E-127	2.5E-124
	Fyb	0.331	0.786	0.682	1.7E-118	2.6E-115
	Saa3	0.560	0.748	0.606	4.7E-83	7.1E-80
	Ly6g	0.520	0.596	0.425	4.1E-82	6.1E-79
	Tgfbi	0.391	0.696	0.596	1.9E-80	2.8E-77
	Gm5416	0.363	0.605	0.486	5.7E-76	8.5E-73
	Pi16	0.460	0.605	0.450	1.1E-70	1.6E-67
	Fcgr4	0.341	0.715	0.693	1.4E-60	2.0E-57
	Slc6a6	0.335	0.651	0.489	4.4E-51	6.6E-48
	Csf2ra	0.339	0.671	0.553	1.6E-47	2.4E-44
	Fgd4	0.308	0.677	0.628	5.9E-39	8.9E-36
	Stfa3	0.456	0.528	0.544	1.1E-08	1.7E-05
	Hacd4	0.323	0.496	0.524	8.5E-08	1.3E-04
	Ptgs2	0.592	0.407	0.275	1.3E-07	2.0E-04
	Fosb	0.422	0.476	0.366	3.8E-03	1.0E+00
**Neu-3**	Ccl3	2.965	0.920	0.610	0.0E+00	0.0E+00
	Hcar2	2.705	0.884	0.657	0.0E+00	0.0E+00
	Ccl4	2.320	0.868	0.702	0.0E+00	0.0E+00
	Nceh1	2.161	0.856	0.686	0.0E+00	0.0E+00
	Gadd45b	2.109	0.877	0.741	0.0E+00	0.0E+00
	Ifrd1	1.995	0.819	0.702	0.0E+00	0.0E+00
	Hilpda	1.887	0.836	0.728	0.0E+00	0.0E+00
	Atp6v1c1	1.859	0.857	0.741	0.0E+00	0.0E+00
	Zeb2	1.831	0.829	0.607	0.0E+00	0.0E+00
	Cd63	1.769	0.936	0.766	0.0E+00	0.0E+00
	Ftl1	1.745	1.000	0.953	0.0E+00	0.0E+00
	Id2	1.629	0.874	0.771	0.0E+00	0.0E+00
	F10	1.608	0.863	0.694	0.0E+00	0.0E+00
	Ctsz	1.414	0.936	0.780	0.0E+00	0.0E+00
	Ctsb	1.241	0.977	0.941	0.0E+00	0.0E+00
	Gas2l3	1.116	0.818	0.634	0.0E+00	0.0E+00
	Dhfr	0.942	0.789	0.739	0.0E+00	0.0E+00
	Lhfpl2	0.915	0.784	0.664	0.0E+00	0.0E+00
	Ctsd	0.494	0.987	0.963	0.0E+00	0.0E+00
	Fcgr2b	0.726	0.838	0.637	1.2E-307	1.8E-304
	Chka	0.537	0.738	0.519	3.0E-302	4.5E-299
	H2-Eb1	1.000	0.683	0.519	1.0E-285	1.5E-282
	Cd274	1.549	0.725	0.579	2.4E-265	3.6E-262
	Plcxd2	0.637	0.728	0.653	3.2E-265	4.8E-262
	Hexa	0.947	0.839	0.688	1.1E-248	1.7E-245
	Dock10	0.893	0.783	0.666	2.7E-239	4.1E-236
	Pdxk	0.597	0.726	0.574	7.5E-236	1.1E-232
	Cxcl2	1.230	0.759	0.512	6.9E-233	1.0E-229
	Psap	0.760	0.852	0.749	1.3E-230	1.9E-227
	Gns	1.339	0.782	0.688	1.3E-230	2.0E-227
	Lamp1	1.194	0.885	0.861	2.6E-229	3.9E-226
	Atf3	1.754	0.679	0.548	3.1E-224	4.7E-221
	Npc1	1.211	0.749	0.677	5.2E-220	7.8E-217
	Ccnf	0.536	0.662	0.612	4.8E-210	7.3E-207
	Plekhm2	1.096	0.785	0.726	1.7E-206	2.5E-203
	Rgs1	1.675	0.774	0.615	1.1E-205	1.6E-202
	Ier3	1.085	0.810	0.786	1.8E-195	2.7E-192
	9130230L23Rik	0.335	0.648	0.524	1.2E-192	1.8E-189
	P2rx7	0.626	0.760	0.609	9.9E-177	1.5E-173
	Fam20c	0.499	0.847	0.758	1.7E-170	2.6E-167
	Gstm1	0.562	0.697	0.550	1.0E-164	1.6E-161
	Hmox1	1.389	0.790	0.754	7.4E-133	1.1E-129
	Aprt	0.932	0.820	0.731	3.6E-132	5.4E-129
	Tnfrsf12a	0.301	0.721	0.595	1.8E-125	2.7E-122
	Tpp1	0.909	0.716	0.608	4.3E-113	6.5E-110
	Inhba	0.910	0.622	0.550	4.4E-112	6.5E-109
	Tst	0.398	0.688	0.590	1.2E-95	1.9E-92
	Tcirg1	1.119	0.670	0.616	9.4E-90	1.4E-86
	Canx	0.712	0.769	0.666	1.2E-87	1.8E-84
	Mpeg1	0.696	0.701	0.614	6.5E-80	9.7E-77
	Clu	2.509	0.912	0.787	1.7E-182	2.5E-179
	Avpi1	1.828	0.900	0.732	2.4E-182	3.6E-179
	Cp	1.550	0.766	0.560	1.4E-168	2.1E-165
	Cald1	1.304	0.837	0.684	1.2E-164	1.8E-161
	Mgp	2.757	0.817	0.644	3.8E-162	5.7E-159
	Cbr2	3.030	0.806	0.745	6.7E-160	1.0E-156
	Ybx3	1.340	0.857	0.598	6.7E-160	1.0E-156
	Far1	1.422	0.882	0.509	6.9E-160	1.0E-156
	Plac8	1.530	0.919	0.554	8.7E-160	1.3E-156
	Serping1	1.481	0.693	0.306	5.8E-159	8.7E-156
	Akr1b3	1.620	0.866	0.517	1.5E-157	2.2E-154
	Cd81	1.439	0.922	0.607	1.0E-155	1.5E-152
	Scgb1a1	3.980	0.915	0.811	7.6E-155	1.1E-151
	Id3	1.470	0.769	0.565	1.6E-149	2.5E-146
	Perp	1.659	0.834	0.733	1.2E-147	1.8E-144
	Ppp1r14b	1.481	0.892	0.660	6.9E-146	1.0E-142
	Tpm2	1.173	0.815	0.658	4.9E-138	7.4E-135
	Ceacam1	1.300	0.918	0.689	2.4E-135	3.5E-132
	Igfbp4	1.213	0.714	0.516	3.6E-125	5.4E-122
	Rpl3	1.482	0.892	0.712	1.4E-118	2.1E-115
	Ptprf	1.412	0.746	0.622	2.3E-117	3.4E-114
	Rps2	1.263	0.934	0.894	1.5E-115	2.2E-112
	Nenf	1.188	0.790	0.520	6.5E-109	9.7E-106
	Arg1	1.174	0.919	0.760	1.7E-105	2.6E-102
	Nedd4	1.526	0.696	0.323	1.8E-105	2.8E-102
	Dmkn	1.828	0.776	0.719	1.0E-104	1.6E-101
	Fabp5	2.134	0.923	0.803	7.8E-103	1.2E-99
	Gm26870	1.224	0.813	0.564	1.2E-101	1.8E-98
	Eln	1.022	0.671	0.350	3.1E-100	4.6E-97
	Scgb3a1	4.567	0.740	0.635	1.2E-99	1.8E-96
	Pam	1.544	0.788	0.651	1.9E-97	2.9E-94
	Aqp5	1.174	0.777	0.728	4.3E-96	6.5E-93
	Phlda1	1.105	0.823	0.551	4.7E-95	7.1E-92
	Prdx2	1.395	0.805	0.642	2.1E-89	3.1E-86
	Epcam	1.512	0.786	0.644	4.0E-89	6.0E-86
	Dcxr	1.400	0.768	0.619	4.5E-87	6.8E-84
	Serpinb6b	1.063	0.769	0.688	5.7E-86	8.6E-83
	Rbp1	1.619	0.722	0.552	4.9E-85	7.3E-82
	Muc5b	2.403	0.675	0.506	7.2E-84	1.1E-80
	Scd1	1.002	0.226	0.360	1.4E-83	2.2E-80
	Tsc22d1	1.408	0.780	0.747	1.2E-81	1.9E-78
	Socs2	1.111	0.732	0.667	2.5E-80	3.8E-77
	Selenbp1	2.144	0.707	0.645	4.2E-80	6.3E-77
	Krt8	1.959	0.720	0.698	5.2E-77	7.9E-74
	Atp5g1	1.265	0.834	0.645	8.9E-76	1.3E-72
	Prdx1	1.233	0.822	0.667	1.8E-74	2.6E-71
	Sftpb	1.227	0.664	0.439	2.9E-74	4.4E-71
	Retnla	1.246	0.738	0.750	1.6E-71	2.3E-68
	Nfib	1.180	0.680	0.619	2.7E-70	4.1E-67
	Aldh1a1	1.071	0.648	0.384	9.0E-69	1.3E-65
	Nupr1	1.463	0.795	0.711	1.2E-68	1.8E-65
	Ifitm3	1.335	0.809	0.700	6.7E-68	1.0E-64
	Sdc4	1.174	0.760	0.674	1.7E-65	2.5E-62
	Cldn3	1.119	0.776	0.778	5.3E-64	8.0E-61
	Scd2	1.073	0.705	0.541	6.5E-63	9.7E-60
	Timp3	1.525	0.657	0.569	1.1E-59	1.6E-56
	Scgb3a2	2.769	0.668	0.618	4.3E-58	6.5E-55
	Dbi	1.376	0.776	0.646	7.1E-52	1.1E-48
	Tmem176b	1.986	0.660	0.567	2.6E-51	3.9E-48
	Sdc1	1.649	0.700	0.709	1.3E-43	2.0E-40
	Cyp2f2	2.315	0.663	0.704	2.5E-42	3.7E-39
	Bgn	1.569	0.550	0.243	1.1E-39	1.7E-36
	Trf	1.356	0.684	0.507	1.6E-37	2.5E-34
	Wfdc18	1.473	0.361	0.563	6.2E-34	9.4E-31
	Sparc	2.443	0.542	0.358	1.0E-32	1.6E-29
	Foxq1	1.159	0.583	0.537	2.1E-25	3.2E-22
	Tff2	1.506	0.544	0.381	1.1E-24	1.7E-21
	Ndufc2	1.065	0.660	0.625	1.1E-23	1.7E-20
	Chchd10	1.241	0.644	0.644	2.8E-22	4.3E-19
	Igfbp5	1.952	0.347	0.441	9.8E-22	1.5E-18
	Ccnd1	1.370	0.572	0.514	2.1E-19	3.2E-16
	Tagln2	1.084	0.659	0.593	5.6E-18	8.5E-15
	Hmgn1	1.450	0.616	0.655	9.1E-17	1.4E-13
	Snhg18	1.056	0.551	0.593	1.2E-10	1.7E-07
	C3	1.081	0.651	0.554	3.2E-06	4.8E-03

	Tmem86a	0.586	0.724	0.687	1.8E-78	2.7E-75
	Osm	0.502	0.286	0.341	5.9E-76	8.8E-73
	C3	0.873	0.632	0.548	3.4E-72	5.1E-69
	Slc7a11	1.622	0.565	0.458	4.8E-70	7.2E-67
	Acod1	1.455	0.548	0.408	1.2E-65	1.8E-62
	Ftl1-ps1	0.517	0.338	0.440	5.7E-65	8.5E-62
	Naglu	0.833	0.616	0.547	7.7E-61	1.2E-57
	Gm5416	0.294	0.605	0.491	3.2E-55	4.8E-52
	Prok2	0.362	0.643	0.575	3.5E-50	5.2E-47
	Cd300c2	0.878	0.675	0.658	2.2E-49	3.3E-46
	Hk2	0.718	0.466	0.600	6.9E-44	1.0E-40
	Slc43a3	0.347	0.577	0.585	8.1E-44	1.2E-40
	Creg1	1.161	0.702	0.668	6.8E-39	1.0E-35
	Cldn1	0.605	0.582	0.623	1.3E-37	2.0E-34
	Hspa1b	0.760	0.659	0.609	2.5E-37	3.8E-34
	G0s2	0.733	0.597	0.488	6.5E-37	9.7E-34
	Tgm2	0.386	0.453	0.630	1.1E-35	1.7E-32
	Ctsa	0.424	0.748	0.700	1.5E-35	2.2E-32
	Cxcr1	0.882	0.643	0.718	9.1E-35	1.4E-31
	Gadd45g	0.961	0.634	0.584	2.1E-31	3.2E-28
	Cd68	0.694	0.714	0.715	6.3E-31	9.4E-28
	Tnfrsf23	0.997	0.661	0.707	8.2E-31	1.2E-27
	Hal	0.570	0.514	0.710	1.2E-30	1.8E-27
	Syngr1	0.408	0.721	0.777	1.4E-28	2.0E-25
	Ptgs2	0.446	0.299	0.293	4.0E-27	6.0E-24
	Slc37a2	0.659	0.609	0.589	5.6E-27	8.4E-24
	Dpp7	0.410	0.624	0.628	5.7E-25	8.6E-22
	Laptm5	0.357	0.748	0.739	3.4E-23	5.1E-20
	Hpgds	0.365	0.680	0.634	4.8E-21	7.2E-18
	Rps6ka2	0.407	0.521	0.487	7.5E-20	1.1E-16
	Slc6a6	0.352	0.448	0.520	6.6E-18	9.9E-15
	Syne1	0.775	0.511	0.619	2.1E-17	3.1E-14
	Vegfa	0.957	0.495	0.433	7.9E-17	1.2E-13
	Asprv1	0.304	0.639	0.584	1.2E-14	1.8E-11
	Sqstm1	0.695	0.608	0.639	3.5E-14	5.2E-11
	Tnf	0.606	0.311	0.311	1.1E-12	1.6E-09
	Gm26870	0.659	0.567	0.570	1.1E-11	1.7E-08
	Hspa9	0.318	0.651	0.648	1.1E-09	1.6E-06
	Osgin1	0.594	0.500	0.600	5.8E-08	8.7E-05
	Csf2rb	0.453	0.681	0.687	7.0E-07	1.1E-03
	Egr1	0.431	0.574	0.554	8.2E-07	1.2E-03
	Dhrs3	0.461	0.674	0.676	1.1E-06	1.7E-03
	Thbs1	1.309	0.451	0.550	7.4E-06	1.1E-02
	Cdkn1a	0.339	0.776	0.770	4.9E-05	7.3E-02
	Gadd45a	0.424	0.639	0.622	1.8E-04	2.6E-01
	Hist1h4i	0.531	0.648	0.669	3.2E-04	4.8E-01
	Slpi	0.477	0.653	0.644	3.7E-04	5.5E-01
	Ddit3	0.444	0.598	0.758	3.7E-04	5.6E-01
	Il1f9	0.929	0.529	0.564	5.8E-04	8.7E-01
	Sirpa	0.299	0.560	0.634	5.2E-03	1.0E+00
**T cells**	Cd3g	2.602	0.918	0.436	0.0E+00	0.0E+00
	Trbc2	2.530	0.904	0.229	0.0E+00	0.0E+00
	Il7r	2.308	0.820	0.669	0.0E+00	0.0E+00
	Bcl2	2.093	0.817	0.414	0.0E+00	0.0E+00
	Rpl3	1.972	0.988	0.699	0.0E+00	0.0E+00
	Cd3e	1.953	0.856	0.286	0.0E+00	0.0E+00
	Cd3d	1.904	0.890	0.375	0.0E+00	0.0E+00
	Ptprcap	1.902	0.828	0.511	0.0E+00	0.0E+00
	Gimap1	1.875	0.851	0.436	0.0E+00	0.0E+00
	Cxcr6	1.867	0.946	0.284	0.0E+00	0.0E+00
	Ctla2a	1.782	0.926	0.738	0.0E+00	0.0E+00
	Trac	1.722	0.781	0.126	0.0E+00	0.0E+00
	Gimap3	1.701	0.790	0.197	0.0E+00	0.0E+00
	AW112010	1.634	0.891	0.489	0.0E+00	0.0E+00
	Ets1	1.568	0.849	0.434	0.0E+00	0.0E+00
	Ms4a6b	1.553	0.803	0.254	0.0E+00	0.0E+00
	Skap1	1.513	0.806	0.163	0.0E+00	0.0E+00
	Lck	1.488	0.779	0.264	0.0E+00	0.0E+00
	Ramp1	1.368	0.783	0.405	0.0E+00	0.0E+00
	Rps2	1.301	0.993	0.889	0.0E+00	0.0E+00
	Nkg7	1.264	0.876	0.521	0.0E+00	0.0E+00
	Ptma	1.233	0.983	0.718	0.0E+00	0.0E+00
	Maf	1.184	0.936	0.701	0.0E+00	0.0E+00
	Thy1	1.172	0.878	0.165	0.0E+00	0.0E+00
	Hspe1	1.096	0.879	0.665	0.0E+00	0.0E+00

	Tst	1.319	0.571	0.602	1.0E-05	1.6E-02
	Pmepa1	1.556	0.563	0.622	5.8E-05	8.7E-02
	Fmo2	1.044	0.413	0.343	2.5E-04	3.8E-01
	Crip2	1.269	0.502	0.560	1.6E-03	1.0E+00
	Igfbp7	1.823	0.413	0.473	5.7E-03	1.0E+00
	Sftpd	1.945	0.489	0.461	8.2E-03	1.0E+00
**T cells 2**	Ccl5	6.355	0.988	0.505	0.0E+00	0.0E+00
	Nkg7	4.438	0.997	0.532	0.0E+00	0.0E+00
	AW112010	3.298	0.997	0.502	0.0E+00	0.0E+00
	Cd3g	2.927	0.963	0.453	0.0E+00	0.0E+00
	Klrd1	2.649	0.930	0.383	0.0E+00	0.0E+00
	Gimap4	2.407	0.904	0.222	0.0E+00	0.0E+00
	Cxcr6	2.039	0.977	0.308	0.0E+00	0.0E+00
	Ikzf3	1.577	0.956	0.261	0.0E+00	0.0E+00
	Pdcd1	1.413	0.989	0.234	0.0E+00	0.0E+00
	Rgs16	0.578	0.966	0.464	0.0E+00	0.0E+00
	Bcl2	2.127	0.963	0.426	1.1E-305	1.6E-302
	Klre1	1.502	0.905	0.518	5.3E-294	8.0E-291
	Gimap1	2.138	0.918	0.450	1.1E-276	1.6E-273
	Ptprcap	2.054	0.919	0.521	6.8E-265	1.0E-261
	Gzma	3.710	0.885	0.291	3.3E-263	5.0E-260
	Serpinb9	1.165	0.887	0.348	3.6E-245	5.4E-242
	Wls	1.997	0.925	0.525	5.8E-242	8.8E-239
	Ctsw	2.064	0.826	0.206	9.3E-235	1.4E-231
	Gpr171	0.922	0.902	0.568	2.0E-234	3.1E-231
	Camk2n1	0.674	0.893	0.584	6.5E-232	9.8E-229
	Klrb1c	0.994	0.792	0.241	1.3E-217	2.0E-214
	Thy1	1.399	0.805	0.194	2.8E-212	4.2E-209
	Itga1	1.174	0.858	0.540	2.9E-207	4.3E-204
	Lck	1.936	0.843	0.282	9.4E-198	1.4E-194
	Cd3e	1.702	0.850	0.307	4.9E-196	7.4E-193
	Bin1	1.028	0.889	0.559	4.2E-175	6.3E-172
	Rpl3	1.327	0.992	0.710	1.8E-173	2.7E-170
	mt-Nd1	1.057	0.991	0.669	1.2E-168	1.8E-165
	Ctla2a	1.995	0.849	0.746	4.1E-167	6.2E-164
	Gbp4	0.491	0.908	0.599	6.1E-164	9.2E-161
	Skap1	1.419	0.785	0.187	8.8E-160	1.3E-156
	Lgals1	1.245	0.915	0.575	1.1E-159	1.6E-156
	Gimap3	1.887	0.779	0.219	8.9E-157	1.3E-153
	Gpr65	0.821	0.870	0.451	4.5E-153	6.7E-150
	Ets1	1.314	0.838	0.449	9.4E-153	1.4E-149
	Ptma	1.057	0.983	0.728	1.0E-151	1.5E-148
	Il2rb	1.677	0.756	0.242	1.1E-147	1.7E-144
	Crip1	0.729	0.947	0.503	4.0E-140	5.9E-137
	Hspe1	0.981	0.928	0.671	4.3E-139	6.5E-136
	Gzmb	2.047	0.733	0.217	1.6E-136	2.4E-133
	Bcl2a1d	0.761	0.902	0.508	6.0E-129	9.1E-126
	Ly9	0.743	0.798	0.321	9.2E-125	1.4E-121
	Laptm5	0.968	0.994	0.735	1.4E-124	2.2E-121
	Trbc2	2.552	0.763	0.257	3.1E-120	4.7E-117
	Gimap6	1.119	0.771	0.323	5.1E-115	7.7E-112
	Rps2	0.852	0.998	0.893	4.5E-114	6.7E-111
	Trac	1.458	0.734	0.151	8.0E-114	1.2E-110
	AU020206	0.620	0.934	0.698	2.9E-110	4.4E-107
	Irf8	0.532	0.853	0.635	3.3E-106	4.9E-103
	H2afz	0.948	0.944	0.804	2.5E-104	3.7E-101
	Ccnd2	1.530	0.785	0.608	3.8E-104	5.7E-101
	Dut	0.533	0.774	0.448	1.5E-98	2.3E-95
	Emp3	0.746	0.876	0.460	5.1E-97	7.6E-94
	Id2	0.628	0.977	0.778	1.4E-96	2.0E-93
	Ebpl	0.549	0.782	0.407	2.9E-88	4.4E-85
	Ndufa4	0.747	0.879	0.668	4.0E-88	6.0E-85
	Cd2	1.412	0.725	0.421	1.2E-85	1.8E-82
	Ifi47	0.906	0.766	0.648	1.7E-82	2.6E-79
	Klrk1	1.640	0.666	0.100	2.0E-81	3.0E-78
	Serpinb6b	0.741	0.769	0.688	8.2E-73	1.2E-69
	Cx3cr1	0.855	0.716	0.365	3.6E-72	5.4E-69
	Reep5	0.686	0.904	0.713	1.6E-71	2.4E-68
	Pla2g16	0.823	0.780	0.548	3.0E-65	4.5E-62
	Racgap1	0.500	0.689	0.255	1.8E-63	2.7E-60
	Cd7	1.311	0.653	0.123	3.7E-59	5.5E-56
	F2r	1.007	0.699	0.655	1.7E-57	2.5E-54
	Itm2c	0.598	0.789	0.595	2.3E-57	3.5E-54
	Maf	0.484	0.773	0.713	6.1E-56	9.2E-53
	Sh2d1a	1.028	0.637	0.160	4.7E-49	7.0E-46

	Ccl5	1.061	0.893	0.492	0.0E+00	0.0E+00
	Pdcd1	1.058	0.965	0.206	0.0E+00	0.0E+00
	Icos	1.040	0.721	0.224	0.0E+00	0.0E+00
	Il2rb	0.938	0.822	0.219	0.0E+00	0.0E+00
	Trdc	0.926	0.888	0.260	0.0E+00	0.0E+00
	Ikzf3	0.876	0.895	0.238	0.0E+00	0.0E+00
	F2r	0.833	0.807	0.646	0.0E+00	0.0E+00
	mt-Nd1	0.778	0.971	0.657	0.0E+00	0.0E+00
	Tcrg-C1	0.619	0.895	0.175	0.0E+00	0.0E+00
	Cd163l1	0.484	0.811	0.167	0.0E+00	0.0E+00
	Camk2n1	0.476	0.974	0.567	0.0E+00	0.0E+00
	Il2ra	0.462	0.861	0.138	0.0E+00	0.0E+00
	Gbp4	0.441	0.879	0.589	0.0E+00	0.0E+00
	AU020206	0.440	0.957	0.687	0.0E+00	0.0E+00
	Nrp1	0.439	0.886	0.725	0.0E+00	0.0E+00
	Gpr171	0.417	0.782	0.562	3.2E-288	4.8E-285
	S100a4	1.528	0.865	0.488	9.4E-267	1.4E-263
	Crip1	0.519	0.881	0.490	1.0E-230	1.6E-227
	Ctsw	0.610	0.694	0.191	3.5E-222	5.3E-219
	Slc25a4	0.912	0.791	0.526	7.1E-222	1.1E-218
	Lat	0.926	0.715	0.457	1.0E-202	1.6E-199
	Gimap4	1.294	0.695	0.208	3.3E-200	5.0E-197
	Tcf7	0.897	0.659	0.050	2.1E-199	3.1E-196
	Gimap6	0.852	0.713	0.309	4.4E-194	6.6E-191
	Cd28	1.003	0.702	0.529	1.9E-184	2.8E-181
	Bcl11b	1.081	0.686	0.332	1.0E-181	1.6E-178
	Ebpl	0.434	0.744	0.395	1.2E-177	1.7E-174
	Itgb7	1.071	0.709	0.520	5.7E-170	8.5E-167
	Wls	0.694	0.775	0.518	1.0E-164	1.6E-161
	Cd2	1.084	0.694	0.411	6.8E-162	1.0E-158
	Gpr65	0.404	0.737	0.442	2.0E-150	3.0E-147
	4930523C07Ril	0.862	0.693	0.505	1.3E-137	1.9E-134
	Pla2g16	0.610	0.767	0.540	8.1E-130	1.2E-126
	Pgls	0.429	0.820	0.627	5.4E-121	8.1E-118
	Dut	0.633	0.647	0.443	7.5E-88	1.1E-84
	Cd7	0.448	0.374	0.119	7.0E-70	1.1E-66
	Ifi203	0.760	0.642	0.560	2.2E-69	3.3E-66
	Il18r1	0.468	0.359	0.433	9.1E-69	1.4E-65
	Ranbp1	0.564	0.715	0.643	6.1E-68	9.1E-65
	Mllt3	0.629	0.666	0.631	2.6E-67	3.8E-64
	Ar	0.464	0.380	0.208	8.1E-65	1.2E-61
	Ccnd2	0.721	0.677	0.608	5.6E-64	8.3E-61
	Tmem176a	0.693	0.737	0.580	1.5E-62	2.3E-59
	Igfbp4	0.442	0.371	0.530	4.0E-61	6.0E-58
	Hmgn1	0.444	0.702	0.651	8.9E-57	1.3E-53
	Nucks1	0.698	0.651	0.530	2.2E-56	3.2E-53
	Ifi27l2a	0.810	0.649	0.527	1.1E-55	1.7E-52
	Nrip1	0.824	0.664	0.612	7.0E-54	1.1E-50
	Esyt1	0.550	0.636	0.410	6.1E-53	9.1E-50
	Ifi47	0.471	0.662	0.649	1.9E-50	2.9E-47
	Dock10	0.556	0.688	0.678	8.2E-47	1.2E-43
	Trbc1	1.908	0.580	0.202	3.3E-44	4.9E-41
	C1qbp	0.468	0.666	0.664	9.5E-43	1.4E-39
	Ms4a4b	0.952	0.551	0.088	2.1E-41	3.2E-38
	Gm12840	1.196	0.572	0.386	9.3E-39	1.4E-35
	Gimap7	0.743	0.566	0.222	1.6E-32	2.4E-29
	Klk8	0.546	0.598	0.493	8.8E-32	1.3E-28
	Rexo2	0.515	0.636	0.637	1.1E-25	1.6E-22
	Zbtb20	0.641	0.604	0.540	1.0E-24	1.5E-21
	Evl	0.589	0.582	0.337	5.4E-18	8.2E-15
	Las1l	0.388	0.576	0.490	5.6E-17	8.4E-14
	Kcnq1ot1	0.582	0.619	0.658	8.6E-16	1.3E-12
	S1pr1	0.739	0.552	0.507	8.8E-16	1.3E-12
	Lef1	0.517	0.517	0.035	3.6E-14	5.4E-11
	Gpr183	0.598	0.436	0.265	9.7E-11	1.5E-07
	Kcnn4	0.510	0.468	0.579	5.0E-10	7.6E-07
	Gm8369	0.566	0.443	0.091	1.8E-09	2.8E-06
	Tnfsf8	0.669	0.451	0.396	5.5E-09	8.3E-06
	Ramp3	0.410	0.540	0.413	1.2E-07	1.8E-04
	Ccr2	0.445	0.449	0.261	4.5E-07	6.8E-04
	Tmem160	0.367	0.600	0.584	5.6E-07	8.5E-04
	Dapl1	0.839	0.526	0.394	4.6E-06	6.9E-03
	Cblb	0.388	0.487	0.637	1.4E-04	2.1E-01
	Odc1	0.426	0.573	0.569	1.2E-03	1.0E+00
	Tmem176b	0.675	0.581	0.569	5.8E-03	1.0E+00

	Cd3d	1.570	0.660	0.399	1.5E-48	2.3E-45
	Nme1	0.638	0.798	0.715	6.0E-44	9.0E-41
	Cd48	1.146	0.658	0.445	9.0E-42	1.3E-38
	Pycard	0.597	0.864	0.778	3.7E-39	5.5E-36
	Zbp1	0.508	0.708	0.633	3.6E-36	5.5E-33
	Itgb7	0.911	0.655	0.528	4.8E-34	7.2E-31
	Gimap7	1.216	0.618	0.234	2.7E-31	4.0E-28
	Tmem160	0.727	0.687	0.583	2.4E-28	3.7E-25
	Phf11b	0.550	0.623	0.316	8.7E-28	1.3E-24
	Dock10	0.734	0.696	0.678	3.1E-27	4.7E-24
	Klra4	1.070	0.347	0.141	2.8E-26	4.2E-23
	Kcnq1ot1	0.612	0.692	0.655	1.8E-18	2.6E-15
	Nucks1	0.618	0.643	0.535	1.4E-17	2.1E-14
	Ranbp1	0.579	0.682	0.647	3.9E-15	5.9E-12
	Trbc1	1.609	0.582	0.216	9.1E-15	1.4E-11
	Osbpl3	0.642	0.631	0.611	1.3E-13	1.9E-10
	AC149090.1	0.829	0.602	0.458	1.3E-12	2.0E-09
	Bcl11b	1.186	0.573	0.347	3.4E-12	5.1E-09
	Sept9	0.475	0.618	0.475	1.9E-10	2.8E-07
	Il18r1	0.691	0.586	0.426	6.5E-10	9.8E-07
	Il7r	0.709	0.631	0.679	2.1E-09	3.1E-06
	S100a4	1.032	0.598	0.508	3.6E-09	5.4E-06
	Evl	0.553	0.597	0.346	4.4E-08	6.6E-05
	Dok2	0.675	0.563	0.438	3.4E-07	5.0E-04
	Sms	0.505	0.435	0.441	5.8E-07	8.7E-04
	Ms4a6b	0.880	0.574	0.280	7.1E-07	1.1E-03
	Ybx3	0.569	0.449	0.607	4.5E-06	6.7E-03
	Cblb	0.518	0.449	0.632	7.0E-06	1.0E-02
	Esyt1	0.697	0.566	0.420	1.5E-05	2.2E-02
	Sept11	0.805	0.568	0.594	8.0E-05	1.2E-01
	Slamf7	0.845	0.534	0.547	8.7E-03	1.0E+00
**Prol.**	Pclaf	2.491	0.958	0.145	0.0E+00	0.0E+00
	Tpx2	1.659	0.979	0.461	0.0E+00	0.0E+00
	Hmmr	1.416	0.965	0.417	0.0E+00	0.0E+00
	Cks1b	1.929	0.966	0.434	5.1E-307	7.7E-304
	Ccnb1	1.412	0.897	0.279	9.5E-295	1.4E-291
	Spc25	1.049	0.954	0.304	7.3E-293	1.1E-289
	Stmn1	2.414	0.965	0.511	2.1E-290	3.1E-287
	Mki67	2.558	0.959	0.570	2.8E-289	4.3E-286
	Nusap1	1.629	0.920	0.239	1.2E-271	1.7E-268
	Cdca8	1.719	0.927	0.543	1.2E-265	1.8E-262
	Tuba1b	2.789	0.975	0.597	1.6E-247	2.4E-244
	Cenpe	1.483	0.904	0.409	3.6E-245	5.4E-242
	Racgap1	1.111	0.892	0.252	2.0E-234	3.0E-231
	Ptma	2.106	0.961	0.729	9.1E-234	1.4E-230
	Lig1	1.309	0.922	0.604	1.5E-224	2.2E-221
	Comt	1.054	0.981	0.624	1.5E-214	2.2E-211
	Fn1	1.115	0.950	0.408	2.4E-214	3.6E-211
	Mt1	1.467	0.989	0.771	5.7E-214	8.6E-211
	Tacc3	1.088	0.885	0.465	6.6E-212	1.0E-208
	Nucks1	2.033	0.961	0.529	6.2E-211	9.3E-208
	H2afz	1.932	0.933	0.805	2.0E-204	3.0E-201
	Tagln2	2.069	0.991	0.587	7.3E-196	1.1E-192
	Snx5	1.077	0.986	0.545	5.5E-194	8.2E-191
	Tubb5	2.638	0.927	0.597	1.1E-193	1.6E-190
	Hmgn1	1.810	0.970	0.649	2.7E-191	4.0E-188
	Atad2	1.082	0.929	0.479	9.4E-191	1.4E-187
	Ppp1r14b	1.888	0.981	0.660	1.3E-189	1.9E-186
	Lgals1	1.754	0.982	0.574	1.6E-188	2.4E-185
	Slc25a4	1.447	0.961	0.533	1.2E-185	1.8E-182
	Ccnb2	1.586	0.846	0.228	4.4E-185	6.7E-182
	Hebp1	1.259	0.989	0.644	2.7E-183	4.1E-180
	Ezh2	1.288	0.869	0.522	1.3E-180	2.0E-177
	Crip1	1.624	0.982	0.504	1.2E-176	1.8E-173
	Tmem256	1.475	0.968	0.545	1.8E-173	2.7E-170
	Prdx1	1.850	0.996	0.665	5.2E-173	7.7E-170
	Selenoh	1.849	0.871	0.469	1.9E-168	2.8E-165
	Cenpa	1.756	0.867	0.426	3.0E-166	4.4E-163
	Cenpx	1.309	0.959	0.664	6.9E-165	1.0E-161
	Rexo2	1.263	0.965	0.631	8.8E-164	1.3E-160
	Ndufa4	2.100	0.982	0.667	3.8E-158	5.8E-155
	Pycard	1.529	0.989	0.776	2.6E-157	4.0E-154
	Smc4	1.764	0.924	0.614	5.8E-155	8.7E-152
	Fabp5	1.449	0.993	0.803	1.1E-154	1.7E-151
	Cks2	1.430	0.931	0.464	1.4E-152	2.2E-149

	Igfbp4	0.442	0.371	0.530	4.0E-61	6.0E-58
	Hmgn1	0.444	0.702	0.651	8.9E-57	1.3E-53
	Nucks1	0.698	0.651	0.530	2.2E-56	3.2E-53
	Ifi27l2a	0.810	0.649	0.527	1.1E-55	1.7E-52
	Nrip1	0.824	0.664	0.612	7.0E-54	1.1E-50
	Esyt1	0.550	0.636	0.410	6.1E-53	9.1E-50
	Ifi47	0.471	0.662	0.649	1.9E-50	2.9E-47
	Dock10	0.556	0.688	0.678	8.2E-47	1.2E-43
	Trbc1	1.908	0.580	0.202	3.3E-44	4.9E-41
	C1qbp	0.468	0.666	0.664	9.5E-43	1.4E-39
	Ms4a4b	0.952	0.551	0.088	2.1E-41	3.2E-38
	Gm12840	1.196	0.572	0.386	9.3E-39	1.4E-35
	Gimap7	0.743	0.566	0.222	1.6E-32	2.4E-29
	Klk8	0.546	0.598	0.493	8.8E-32	1.3E-28
	Rexo2	0.515	0.636	0.637	1.1E-25	1.6E-22
	Zbtb20	0.641	0.604	0.540	1.0E-24	1.5E-21
	Evl	0.589	0.582	0.337	5.4E-18	8.2E-15
	Las1l	0.388	0.576	0.490	5.6E-17	8.4E-14
	Kcnq1ot1	0.582	0.619	0.658	8.6E-16	1.3E-12
	S1pr1	0.739	0.552	0.507	8.8E-16	1.3E-12
	Lef1	0.517	0.517	0.035	3.6E-14	5.4E-11
	Gpr183	0.598	0.436	0.265	9.7E-11	1.5E-07
	Kcnn4	0.510	0.468	0.579	5.0E-10	7.6E-07
	Gm8369	0.566	0.443	0.091	1.8E-09	2.8E-06
	Tnfsf8	0.669	0.451	0.396	5.5E-09	8.3E-06
	Ramp3	0.410	0.540	0.413	1.2E-07	1.8E-04
	Ccr2	0.445	0.449	0.261	4.5E-07	6.8E-04
	Tmem160	0.367	0.600	0.584	5.6E-07	8.5E-04
	Dapl1	0.839	0.526	0.394	4.6E-06	6.9E-03
	Cblb	0.388	0.487	0.637	1.4E-04	2.1E-01
	Odc1	0.426	0.573	0.569	1.2E-03	1.0E+00
	Tmem176b	0.675	0.581	0.569	5.8E-03	1.0E+00
**Mac 1**	Apoe	3.645	0.999	0.776	0.0E+00	0.0E+00
	Ctss	3.156	1.000	0.646	0.0E+00	0.0E+00
	Fn1	3.070	0.961	0.389	0.0E+00	0.0E+00
	Lyz2	2.912	1.000	0.750	0.0E+00	0.0E+00
	Trem2	2.398	0.999	0.563	0.0E+00	0.0E+00
	Lgals1	2.386	1.000	0.559	0.0E+00	0.0E+00
	Spp1	2.356	0.996	0.780	0.0E+00	0.0E+00
	Mafb	2.251	0.977	0.576	0.0E+00	0.0E+00
	C1qa	2.202	0.980	0.573	0.0E+00	0.0E+00
	Fabp5	2.165	0.994	0.796	0.0E+00	0.0E+00
	C1qb	2.126	0.982	0.493	0.0E+00	0.0E+00
	Psap	2.095	1.000	0.747	0.0E+00	0.0E+00
	Ctsl	2.035	0.999	0.840	0.0E+00	0.0E+00
	C1qc	2.002	0.983	0.633	0.0E+00	0.0E+00
	Gpnmb	1.975	0.994	0.642	0.0E+00	0.0E+00
	Vim	1.894	0.998	0.631	0.0E+00	0.0E+00
	Ms4a6c	1.887	0.981	0.393	0.0E+00	0.0E+00
	Emp3	1.866	0.993	0.440	0.0E+00	0.0E+00
	Plin2	1.859	1.000	0.843	0.0E+00	0.0E+00
	Cd68	1.847	0.999	0.699	0.0E+00	0.0E+00
	F13a1	1.817	0.892	0.611	0.0E+00	0.0E+00
	Lgmn	1.795	0.994	0.615	0.0E+00	0.0E+00
	Grn	1.771	1.000	0.840	0.0E+00	0.0E+00
	S100a4	1.755	0.940	0.486	0.0E+00	0.0E+00
	Lrp1	1.691	0.995	0.503	0.0E+00	0.0E+00
	Ms4a7	1.620	0.981	0.421	0.0E+00	0.0E+00
	mt-Nd1	1.606	0.996	0.658	0.0E+00	0.0E+00
	Hexa	1.594	0.999	0.688	0.0E+00	0.0E+00
	Npc2	1.576	1.000	0.885	0.0E+00	0.0E+00
	Hexb	1.575	0.998	0.741	0.0E+00	0.0E+00
	Crip1	1.546	0.994	0.486	0.0E+00	0.0E+00
	Lamp1	1.530	1.000	0.856	0.0E+00	0.0E+00
	Saa3	1.523	0.868	0.613	0.0E+00	0.0E+00
	Mgst1	1.502	0.992	0.589	0.0E+00	0.0E+00
	Syngr1	1.501	0.994	0.759	0.0E+00	0.0E+00
	Ctsz	1.495	1.000	0.786	0.0E+00	0.0E+00
	Ctsb	1.482	1.000	0.942	0.0E+00	0.0E+00
	Ccl9	1.476	0.919	0.528	0.0E+00	0.0E+00
	C3ar1	1.475	0.992	0.552	0.0E+00	0.0E+00
	Pycard	1.449	0.984	0.769	0.0E+00	0.0E+00
	Ctsd	1.430	1.000	0.964	0.0E+00	0.0E+00
	Smpdl3a	1.420	0.995	0.611	0.0E+00	0.0E+00
	Anxa5	1.419	0.999	0.669	0.0E+00	0.0E+00

	Racgap1	1.111	0.892	0.252	2.0E-234	3.0E-231
	Ptma	2.106	0.961	0.729	9.1E-234	1.4E-230
	Lig1	1.309	0.922	0.604	1.5E-224	2.2E-221
	Comt	1.054	0.981	0.624	1.5E-214	2.2E-211
	Fn1	1.115	0.950	0.408	2.4E-214	3.6E-211
	Mt1	1.467	0.989	0.771	5.7E-214	8.6E-211
	Tacc3	1.088	0.885	0.465	6.6E-212	1.0E-208
	Nucks1	2.033	0.961	0.529	6.2E-211	9.3E-208
	H2afz	1.932	0.933	0.805	2.0E-204	3.0E-201
	Tagln2	2.069	0.991	0.587	7.3E-196	1.1E-192
	Snx5	1.077	0.986	0.545	5.5E-194	8.2E-191
	Tubb5	2.638	0.927	0.597	1.1E-193	1.6E-190
	Hmgn1	1.810	0.970	0.649	2.7E-191	4.0E-188
	Atad2	1.082	0.929	0.479	9.4E-191	1.4E-187
	Ppp1r14b	1.888	0.981	0.660	1.3E-189	1.9E-186
	Lgals1	1.754	0.982	0.574	1.6E-188	2.4E-185
	Slc25a4	1.447	0.961	0.533	1.2E-185	1.8E-182
	Ccnb2	1.586	0.846	0.228	4.4E-185	6.7E-182
	Hebp1	1.259	0.989	0.644	2.7E-183	4.1E-180
	Ezh2	1.288	0.869	0.522	1.3E-180	2.0E-177
	Crip1	1.624	0.982	0.504	1.2E-176	1.8E-173
	Tmem256	1.475	0.968	0.545	1.8E-173	2.7E-170
	Prdx1	1.850	0.996	0.665	5.2E-173	7.7E-170
	Selenoh	1.849	0.871	0.469	1.9E-168	2.8E-165
	Cenpa	1.756	0.867	0.426	3.0E-166	4.4E-163
	Cenpx	1.309	0.959	0.664	6.9E-165	1.0E-161
	Rexo2	1.263	0.965	0.631	8.8E-164	1.3E-160
	Ndufa4	2.100	0.982	0.667	3.8E-158	5.8E-155
	Pycard	1.529	0.989	0.776	2.6E-157	4.0E-154
	Smc4	1.764	0.924	0.614	5.8E-155	8.7E-152
	Fabp5	1.449	0.993	0.803	1.1E-154	1.7E-151
	Cks2	1.430	0.931	0.464	1.4E-152	2.2E-149
	Nap1l1	1.688	0.954	0.558	1.7E-148	2.5E-145
	Hspa9	1.285	0.938	0.643	9.9E-148	1.5E-144
	Nme1	1.906	0.965	0.712	1.0E-146	1.5E-143
	Prdx2	1.788	0.940	0.640	2.6E-141	3.9E-138
	Rpl3	1.645	0.952	0.712	2.0E-140	3.0E-137
	Rps2	1.458	0.940	0.894	2.3E-130	3.5E-127
	Spp1	1.101	0.991	0.788	3.2E-128	4.8E-125
	Tubb4b	1.816	0.977	0.715	3.8E-128	5.6E-125
	Cybb	1.037	0.947	0.519	8.9E-128	1.3E-124
	Vim	1.633	0.988	0.644	2.4E-125	3.5E-122
	Mcm7	1.071	0.851	0.556	1.5E-124	2.2E-121
	Ybx3	1.869	0.865	0.599	1.9E-123	2.8E-120
	Siva1	1.363	0.931	0.730	6.4E-120	9.6E-117
	Pgls	1.338	0.950	0.632	5.3E-119	8.0E-116
	Ptms	1.038	0.988	0.620	2.7E-116	4.1E-113
	Fkbp2	1.125	0.931	0.533	4.2E-114	6.3E-111
	Tyms	1.094	0.796	0.470	1.6E-111	2.5E-108
	Lamtor4	1.288	0.982	0.702	9.9E-107	1.5E-103
	Hist1h4d	1.284	0.839	0.676	1.0E-105	1.5E-102
	Dbi	1.394	0.936	0.644	2.0E-103	3.0E-100
	Top2a	2.140	0.749	0.304	1.5E-100	2.3E-97
	mt-Nd1	1.535	0.881	0.672	2.2E-98	3.2E-95
	Birc5	2.119	0.756	0.487	3.7E-96	5.6E-93
	Hsp90b1	1.288	0.991	0.786	4.3E-96	6.5E-93
	Cdca3	1.395	0.733	0.222	6.4E-91	9.6E-88
	Reep5	1.089	0.966	0.713	1.3E-90	2.0E-87
	Gm26917	1.427	0.839	0.566	1.8E-88	2.7E-85
	Aprt	1.160	0.943	0.737	1.0E-83	1.6E-80
	Gzma	1.044	0.791	0.294	5.1E-75	7.7E-72
	C1qbp	1.452	0.809	0.661	1.8E-71	2.7E-68
	Ranbp1	1.666	0.798	0.645	3.8E-71	5.8E-68
	Cdc20	1.193	0.713	0.484	2.7E-70	4.1E-67
	Tmed3	1.101	0.873	0.617	4.5E-69	6.7E-66
	Pdia6	1.130	0.973	0.783	2.8E-67	4.2E-64
	Cenpf	1.498	0.697	0.197	9.5E-65	1.4E-61
	Calr	1.114	0.973	0.770	1.3E-59	1.9E-56
	Ramp1	1.043	0.756	0.421	6.4E-56	9.5E-53
	Atp5g1	1.539	0.749	0.648	3.7E-50	5.5E-47
	Hspd1	1.695	0.731	0.563	1.7E-49	2.6E-46
	Igkc	2.677	0.216	0.301	7.5E-49	1.1E-45
	Igha	1.754	0.694	0.385	6.0E-45	9.0E-42
	Hspe1	1.292	0.779	0.675	1.7E-44	2.6E-41
	Dtymk	1.346	0.694	0.574	2.7E-43	4.1E-40

	Cybb	1.397	0.993	0.501	0.0E+00	0.0E+00
	Ctsa	1.395	0.999	0.689	0.0E+00	0.0E+00
	Fcgr2b	1.362	0.991	0.641	0.0E+00	0.0E+00
	Ms4a6d	1.355	0.957	0.726	0.0E+00	0.0E+00
	Ctsc	1.326	0.970	0.634	0.0E+00	0.0E+00
	Rpl3	1.324	0.999	0.701	0.0E+00	0.0E+00
	Tmem256	1.321	0.978	0.530	0.0E+00	0.0E+00
	Erp29	1.320	0.994	0.701	0.0E+00	0.0E+00
	Lrpap1	1.315	0.993	0.571	0.0E+00	0.0E+00
	Gpx1	1.307	1.000	0.828	0.0E+00	0.0E+00
	Prdx1	1.306	0.998	0.653	0.0E+00	0.0E+00
	Selenop	1.301	0.939	0.505	0.0E+00	0.0E+00
	Ccl6	1.287	0.993	0.618	0.0E+00	0.0E+00
	Itgb5	1.270	0.995	0.664	0.0E+00	0.0E+00
	Snx5	1.244	0.989	0.529	0.0E+00	0.0E+00
	Gusb	1.240	0.981	0.609	0.0E+00	0.0E+00
	Aprt	1.235	0.992	0.727	0.0E+00	0.0E+00
	Dbi	1.213	0.986	0.631	0.0E+00	0.0E+00
	Rps2	1.206	0.998	0.889	0.0E+00	0.0E+00
	Cst3	1.200	0.999	0.812	0.0E+00	0.0E+00
	Atp5g1	1.188	0.996	0.631	0.0E+00	0.0E+00
	Plekho1	1.164	0.955	0.538	0.0E+00	0.0E+00
	Myof	1.160	0.994	0.484	0.0E+00	0.0E+00
	Abca1	1.158	0.989	0.723	0.0E+00	0.0E+00
	Mpeg1	1.121	0.999	0.603	0.0E+00	0.0E+00
	Creg1	1.120	0.999	0.654	0.0E+00	0.0E+00
	Irf8	1.119	0.934	0.623	0.0E+00	0.0E+00
	Cd93	1.117	0.916	0.618	0.0E+00	0.0E+00
	Lamtor4	1.117	0.985	0.692	0.0E+00	0.0E+00
	Pdia6	1.103	0.993	0.775	0.0E+00	0.0E+00
	Fam20c	1.100	0.997	0.755	0.0E+00	0.0E+00
	Hebp1	1.091	0.971	0.633	0.0E+00	0.0E+00
	Hspe1	1.089	0.996	0.660	0.0E+00	0.0E+00
	Fam96a	1.086	0.958	0.547	0.0E+00	0.0E+00
	Tubb5	1.084	0.991	0.582	0.0E+00	0.0E+00
	Sash1	1.076	0.988	0.700	0.0E+00	0.0E+00
	Msr1	1.069	0.965	0.286	0.0E+00	0.0E+00
	Nme1	1.065	0.992	0.702	0.0E+00	0.0E+00
	Fkbp2	1.048	0.993	0.516	0.0E+00	0.0E+00
	Tmem160	1.046	0.970	0.564	0.0E+00	0.0E+00
	Gyg	1.041	0.960	0.639	0.0E+00	0.0E+00
	Gatm	1.037	0.904	0.510	0.0E+00	0.0E+00
	Anxa4	1.008	0.987	0.484	0.0E+00	0.0E+00
	Pgls	1.005	0.988	0.619	0.0E+00	0.0E+00
	Pid1	1.002	0.863	0.576	0.0E+00	0.0E+00
	Ppp1r14b	1.000	0.991	0.648	0.0E+00	0.0E+00
	Ucp2	0.994	0.999	0.719	0.0E+00	0.0E+00
	Aldh2	0.986	0.970	0.496	0.0E+00	0.0E+00
	Tuba1b	0.983	0.991	0.583	0.0E+00	0.0E+00
	Timp2	0.980	0.994	0.767	0.0E+00	0.0E+00
	Tmed3	0.973	0.994	0.602	0.0E+00	0.0E+00
	Zeb2	0.969	0.991	0.611	0.0E+00	0.0E+00
	Sgk1	0.967	0.986	0.632	0.0E+00	0.0E+00
	Lat2	0.960	0.968	0.664	0.0E+00	0.0E+00
	Hal	0.960	0.984	0.673	0.0E+00	0.0E+00
	Ccr2	0.957	0.715	0.248	2.0E-241	3.0E-238
	Ccl2	1.048	0.587	0.454	4.3E-39	6.5E-36
**Macs 2**	Chil3	4.719	0.975	0.671	0.0E+00	0.0E+00
	Gpnmb	3.826	1.000	0.645	0.0E+00	0.0E+00
	Lpl	3.697	0.989	0.496	0.0E+00	0.0E+00
	Ctsk	3.692	0.959	0.550	0.0E+00	0.0E+00
	Mmp12	3.321	0.870	0.537	0.0E+00	0.0E+00
	Lyz2	3.173	1.000	0.752	0.0E+00	0.0E+00
	Psap	3.172	1.000	0.749	0.0E+00	0.0E+00
	Cybb	2.910	1.000	0.504	0.0E+00	0.0E+00
	Crip1	2.846	0.996	0.490	0.0E+00	0.0E+00
	Atp6v0d2	2.784	0.999	0.677	0.0E+00	0.0E+00
	Spp1	2.744	0.996	0.782	0.0E+00	0.0E+00
	Mfge8	2.720	0.895	0.672	0.0E+00	0.0E+00
	Ctss	2.600	0.999	0.649	0.0E+00	0.0E+00
	Fabp5	2.562	0.999	0.797	0.0E+00	0.0E+00
	Ctsl	2.553	0.996	0.841	0.0E+00	0.0E+00
	Ctsd	2.452	1.000	0.964	0.0E+00	0.0E+00

	Camp	4.266	0.267	0.373	3.4E-41	5.1E-38
	Hist1h2ap	1.712	0.662	0.481	8.0E-37	1.2E-33
	Erp29	1.042	0.758	0.715	5.1E-28	7.7E-25
	Ube2c	1.866	0.621	0.424	1.2E-24	1.7E-21
	Serpinb1a	1.100	0.363	0.542	1.4E-23	2.2E-20
	Ngp	3.416	0.294	0.366	1.9E-22	2.8E-19
	H2afx	1.423	0.598	0.441	1.4E-15	2.1E-12
	Prc1	1.160	0.595	0.429	2.0E-15	2.9E-12
	Hist1h2ae	1.473	0.600	0.599	3.2E-09	4.8E-06
	Hist1h1b	2.008	0.556	0.500	1.6E-06	2.4E-03
	Car2	1.424	0.451	0.501	5.3E-05	7.9E-02
	Smc2	1.419	0.545	0.391	1.3E-04	2.0E-01
	Jchain	1.375	0.566	0.380	9.6E-04	1.0E+00
**Neu-5 IFN**	Rsad2	3.882	0.893	0.683	2.2E-159	3.2E-156
	Isg15	2.862	0.914	0.683	9.1E-136	1.4E-132
	Ifitm3	2.426	0.937	0.699	9.8E-131	1.5E-127
	Gbp2	3.508	0.883	0.647	3.1E-123	4.6E-120
	Slfn4	3.032	0.848	0.564	3.0E-118	4.5E-115
	Ifi47	2.533	0.858	0.647	4.5E-108	6.7E-105
	Ifit2	1.494	0.827	0.689	1.6E-98	2.5E-95
	Rtp4	2.650	0.820	0.679	1.4E-96	2.1E-93
	Ifit1bl2	1.106	0.794	0.562	1.8E-92	2.6E-89
	Slfn5	2.500	0.787	0.570	5.6E-89	8.5E-86
	Ifit3b	2.020	0.698	0.421	5.4E-66	8.2E-63
	Ccl4	1.652	0.921	0.717	1.5E-61	2.2E-58
	Acod1	1.515	0.764	0.419	5.1E-59	7.7E-56
	Ifit1	2.933	0.693	0.445	1.8E-55	2.7E-52
	Isg20	2.176	0.731	0.572	2.0E-51	3.0E-48
	Cldn1	0.347	0.777	0.616	3.0E-47	4.4E-44
	Fcgr4	1.090	0.812	0.694	1.6E-45	2.3E-42
	Zbp1	1.271	0.789	0.633	5.8E-45	8.7E-42
	Cmpk2	1.335	0.647	0.457	6.9E-42	1.0E-38
	Wfdc17	0.879	0.916	0.778	1.7E-38	2.6E-35
	Oasl1	2.003	0.668	0.582	1.0E-34	1.5E-31
	Hcar2	0.498	0.830	0.679	1.6E-30	2.4E-27
	Gbp5	1.549	0.660	0.546	6.0E-30	9.0E-27
	Ifit3	2.987	0.612	0.399	9.5E-30	1.4E-26
	Gm4316	0.371	0.279	0.493	1.4E-29	2.1E-26
	Gm12840	0.275	0.201	0.399	4.9E-28	7.3E-25
	Retnlg	0.406	0.835	0.628	9.9E-28	1.5E-24
	G0s2	1.012	0.711	0.497	7.3E-27	1.1E-23
	Fyb	0.799	0.835	0.695	1.1E-26	1.6E-23
	Ffar2	0.272	0.701	0.484	2.4E-26	3.6E-23
	Cd274	1.280	0.736	0.593	5.4E-26	8.2E-23
	Bst2	1.457	0.657	0.508	1.2E-23	1.7E-20
	Egr1	0.572	0.756	0.554	1.1E-22	1.7E-19
	Ccl3	0.827	0.799	0.641	1.1E-22	1.7E-19
	Cxcl2	1.001	0.741	0.536	4.1E-22	6.2E-19
	Cxcl10	1.921	0.551	0.311	1.4E-21	2.2E-18
	Ier3	0.726	0.820	0.788	1.7E-21	2.5E-18
	Gm20234	0.309	0.609	0.491	1.1E-18	1.6E-15
	Ly6i	0.652	0.586	0.455	1.5E-18	2.2E-15
	Usp18	1.392	0.604	0.553	2.2E-18	3.4E-15
	Clec4a3	0.263	0.614	0.448	7.7E-17	1.1E-13
	Slpi	0.712	0.797	0.643	1.8E-16	2.6E-13
	Gm5483	0.695	0.728	0.616	2.9E-16	4.3E-13
	Olfm4	0.430	0.635	0.476	1.4E-15	2.1E-12
	Saa3	0.296	0.764	0.624	1.7E-15	2.6E-12
	Asprv1	0.545	0.734	0.588	2.1E-15	3.1E-12
	Gm19951	0.676	0.769	0.685	5.5E-15	8.3E-12
	Ifi202b	0.285	0.739	0.658	5.6E-15	8.4E-12
	Ifitm1	0.593	0.807	0.738	8.3E-15	1.2E-11
	Osgin1	0.663	0.678	0.588	7.4E-14	1.1E-10
	Fgd4	0.355	0.713	0.634	7.6E-14	1.1E-10
	Lcn2	0.607	0.683	0.610	1.0E-11	1.5E-08
	Cst3	0.478	0.858	0.821	2.9E-11	4.3E-08
	Fam20c	0.267	0.858	0.766	2.1E-10	3.2E-07
	Bcl2a1a	0.523	0.398	0.556	5.1E-10	7.6E-07
	Id2	0.498	0.881	0.780	1.8E-09	2.6E-06
	Wfdc21	0.437	0.713	0.653	1.8E-09	2.6E-06
	Il1f9	0.718	0.632	0.560	2.7E-09	4.0E-06
	Laptm5	0.349	0.812	0.739	3.7E-08	5.6E-05
	Id1	0.788	0.688	0.693	5.4E-08	8.1E-05

	Fabp4	2.405	0.976	0.611	0.0E+00	0.0E+00
	Lipa	2.389	0.997	0.513	0.0E+00	0.0E+00
	Mgll	2.382	0.973	0.587	0.0E+00	0.0E+00
	Trem2	2.379	0.995	0.566	0.0E+00	0.0E+00
	Mpeg1	2.367	0.999	0.606	0.0E+00	0.0E+00
	Vim	2.330	0.999	0.634	0.0E+00	0.0E+00
	Sgk1	2.323	0.999	0.634	0.0E+00	0.0E+00
	mt-Nd1	2.303	0.998	0.660	0.0E+00	0.0E+00
	S100a1	2.291	0.994	0.634	0.0E+00	0.0E+00
	Ccl6	2.274	0.994	0.621	0.0E+00	0.0E+00
	Abcg1	2.256	0.997	0.645	0.0E+00	0.0E+00
	Mrc1	2.227	0.894	0.408	0.0E+00	0.0E+00
	Myof	2.219	0.995	0.488	0.0E+00	0.0E+00
	Selenop	2.137	0.991	0.506	0.0E+00	0.0E+00
	Pld3	2.132	0.995	0.574	0.0E+00	0.0E+00
	Plin2	2.121	0.999	0.844	0.0E+00	0.0E+00
	Apoe	2.006	0.979	0.778	0.0E+00	0.0E+00
	Anxa5	1.983	0.999	0.671	0.0E+00	0.0E+00
	Cd68	1.963	0.998	0.702	0.0E+00	0.0E+00
	Aig1	1.926	0.981	0.665	0.0E+00	0.0E+00
	Mgst1	1.906	0.996	0.592	0.0E+00	0.0E+00
	Slc7a2	1.859	0.953	0.593	0.0E+00	0.0E+00
	Ear2	1.849	0.881	0.305	0.0E+00	0.0E+00
	Sh3bgrl	1.816	0.991	0.540	0.0E+00	0.0E+00
	Lrp1	1.810	0.995	0.507	0.0E+00	0.0E+00
	Hexa	1.807	0.999	0.690	0.0E+00	0.0E+00
	Gns	1.748	1.000	0.684	0.0E+00	0.0E+00
	Abhd12	1.739	0.990	0.418	0.0E+00	0.0E+00
	Ahnak2	1.738	0.981	0.542	0.0E+00	0.0E+00
	Creg1	1.723	0.999	0.657	0.0E+00	0.0E+00
	Prdx1	1.712	0.996	0.656	0.0E+00	0.0E+00
	Lgals1	1.693	0.991	0.563	0.0E+00	0.0E+00
	Lgmn	1.687	0.991	0.618	0.0E+00	0.0E+00
	Gstm1	1.679	0.977	0.546	0.0E+00	0.0E+00
	Trf	1.674	0.974	0.490	0.0E+00	0.0E+00
	Gm26917	1.673	0.905	0.556	0.0E+00	0.0E+00
	Gusb	1.658	0.988	0.611	0.0E+00	0.0E+00
	Lamp1	1.650	0.999	0.858	0.0E+00	0.0E+00
	Acp5	1.634	0.995	0.601	0.0E+00	0.0E+00
	Vat1	1.632	0.981	0.642	0.0E+00	0.0E+00
	F7	1.632	0.925	0.406	0.0E+00	0.0E+00
	Axl	1.612	0.892	0.422	0.0E+00	0.0E+00
	Ctsb	1.608	1.000	0.943	0.0E+00	0.0E+00
	Ctsz	1.605	1.000	0.787	0.0E+00	0.0E+00
	Snx5	1.602	0.992	0.532	0.0E+00	0.0E+00
	Ctsa	1.599	0.999	0.692	0.0E+00	0.0E+00
	Dusp3	1.564	0.986	0.628	0.0E+00	0.0E+00
	Cd36	1.561	0.877	0.480	0.0E+00	0.0E+00
	Myo5a	1.560	0.983	0.491	0.0E+00	0.0E+00
	Aplp2	1.550	0.995	0.630	0.0E+00	0.0E+00
	Il11ra1	1.532	0.958	0.470	0.0E+00	0.0E+00
	Kcnq1ot1	1.510	0.945	0.642	0.0E+00	0.0E+00
	Sirpa	1.506	0.996	0.609	0.0E+00	0.0E+00
	Itgax	1.499	0.986	0.527	0.0E+00	0.0E+00
	Anxa4	1.494	0.988	0.487	0.0E+00	0.0E+00
	Fstl1	1.486	0.862	0.466	0.0E+00	0.0E+00
	Dnmt3a	1.473	0.946	0.462	0.0E+00	0.0E+00
	Cdo1	1.466	0.918	0.461	0.0E+00	0.0E+00
	Shtn1	1.463	0.978	0.464	0.0E+00	0.0E+00
	Laptm5	1.462	0.999	0.728	0.0E+00	0.0E+00
	Grn	1.460	0.996	0.841	0.0E+00	0.0E+00
	Mertk	1.458	0.955	0.387	0.0E+00	0.0E+00
	Slc6a6	1.456	0.997	0.490	0.0E+00	0.0E+00
	Smpdl3a	1.454	0.996	0.614	0.0E+00	0.0E+00
	Il18	1.438	0.916	0.498	0.0E+00	0.0E+00
	Abcc5	1.434	0.946	0.408	0.0E+00	0.0E+00
	Colgalt1	1.434	0.980	0.501	0.0E+00	0.0E+00

	Csf2rb	0.657	0.721	0.686	8.4E-08	1.3E-04
	Bcl2a1b	0.643	0.751	0.631	3.8E-07	5.7E-04
	Ddx60	1.401	0.528	0.488	5.3E-07	8.0E-04
	Mpeg1	0.362	0.726	0.622	1.2E-06	1.7E-03
	Tnfrsf23	0.333	0.741	0.702	5.0E-06	7.5E-03
	Osm	0.260	0.475	0.334	5.3E-06	8.0E-03
	Ifi27l2a	2.007	0.553	0.533	5.5E-06	8.2E-03
	Cdc42ep3	0.290	0.622	0.527	7.5E-06	1.1E-02
	Il18bp	0.429	0.508	0.450	1.3E-05	2.0E-02
	Irf7	0.399	0.642	0.654	2.9E-05	4.4E-02
	Lrg1	0.382	0.668	0.699	2.1E-04	3.1E-01
	Cd300c2	0.587	0.657	0.660	2.3E-04	3.4E-01
	Hk2	0.508	0.497	0.587	2.8E-04	4.2E-01
	Steap4	0.298	0.586	0.487	3.4E-04	5.1E-01
	Ifi209	0.898	0.563	0.599	4.8E-04	7.3E-01
	Ly6c2	1.077	0.482	0.398	5.2E-04	7.8E-01
	Chil1	0.308	0.429	0.495	7.0E-04	1.0E+00
	Il18	0.297	0.556	0.516	8.5E-04	1.0E+00
	Ly6g	0.309	0.497	0.449	1.5E-03	1.0E+00
	Rgs1	0.948	0.627	0.632	1.9E-03	1.0E+00
	Unc93b1	0.563	0.569	0.521	2.0E-03	1.0E+00
	Hist1h1c	0.327	0.497	0.617	2.6E-03	1.0E+00
	Ifitm6	0.346	0.391	0.472	4.6E-03	1.0E+00
**Eryth.**	Hbb-bs	9.669	1.000	0.782	0.0E+00	0.0E+00
	Hba-a1	8.916	1.000	0.499	0.0E+00	0.0E+00
	Hbb-bt	8.788	0.996	0.481	0.0E+00	0.0E+00
	Hba-a2	8.609	1.000	0.450	0.0E+00	0.0E+00
	Bpgm	4.001	0.837	0.481	0.0E+00	0.0E+00
	Alas2	3.365	0.939	0.531	0.0E+00	0.0E+00
	Snca	2.876	0.949	0.366	0.0E+00	0.0E+00
	Fech	2.293	0.957	0.604	0.0E+00	0.0E+00
	Fam46c	1.861	0.916	0.610	0.0E+00	0.0E+00
	Rsad2	1.161	0.865	0.679	0.0E+00	0.0E+00
	Gypa	0.702	0.791	0.028	0.0E+00	0.0E+00
	Slc4a1	0.430	0.640	0.027	0.0E+00	0.0E+00
	Prdx2	1.533	0.891	0.636	2.0E-288	3.0E-285
	Fam213a	0.874	0.916	0.568	2.3E-282	3.5E-279
	Isg20	1.050	0.910	0.561	2.0E-245	2.9E-242
	Ncoa4	0.766	0.907	0.558	3.4E-228	5.0E-225
	Aldh1a1	0.377	0.748	0.376	5.6E-212	8.4E-209
	Car2	0.406	0.781	0.489	6.8E-212	1.0E-208
	Ube2c	0.553	0.642	0.419	4.5E-87	6.8E-84
	Gpx1	0.918	0.898	0.834	2.7E-17	4.0E-14
**Macs 3**	Lyz2	3.103	0.994	0.755	0.0E+00	0.0E+00
	Atp6v0d2	2.835	0.929	0.683	0.0E+00	0.0E+00
	Fabp5	2.818	0.976	0.801	0.0E+00	0.0E+00
	Fabp4	2.446	0.907	0.618	0.0E+00	0.0E+00
	Trem2	2.287	0.948	0.573	0.0E+00	0.0E+00
	Mmp12	2.260	0.889	0.540	0.0E+00	0.0E+00
	Vim	2.236	0.943	0.640	0.0E+00	0.0E+00
	Gpnmb	2.203	0.955	0.651	0.0E+00	0.0E+00
	Ftl1	1.976	1.000	0.956	0.0E+00	0.0E+00
	C1qa	1.299	0.907	0.583	0.0E+00	0.0E+00
	Il11ra1	1.068	0.868	0.479	1.4E-302	2.1E-299
	Lgals1	2.459	0.899	0.571	2.8E-293	4.2E-290
	C1qb	1.319	0.890	0.505	4.2E-288	6.2E-285
	Lpl	2.532	0.875	0.506	1.6E-278	2.4E-275
	Cdkn2a	0.952	0.852	0.657	1.9E-278	2.9E-275
	Pld3	1.832	0.903	0.582	6.5E-275	9.7E-272
	Crip1	2.187	0.866	0.500	1.9E-269	2.8E-266
	Serpinb6a	2.298	0.917	0.672	1.5E-268	2.3E-265
	Spp1	2.531	0.948	0.786	4.3E-264	6.4E-261
	Prdx1	2.470	0.877	0.664	4.4E-258	6.7E-255
	Sdc3	1.041	0.862	0.567	9.7E-254	1.5E-250
	S100a1	2.732	0.862	0.643	1.9E-248	2.8E-245
	Myof	1.428	0.860	0.499	6.7E-247	1.0E-243
	Ctsk	2.393	0.836	0.559	2.3E-238	3.5E-235

	Ucp2	1.423	0.999	0.721	0.0E+00	0.0E+00
	Serpinb6a	1.419	0.997	0.665	0.0E+00	0.0E+00
	Itgb2	1.419	0.996	0.577	0.0E+00	0.0E+00
	Cd63	1.419	0.999	0.774	0.0E+00	0.0E+00
	Dbi	1.415	0.987	0.634	0.0E+00	0.0E+00
	Tgfbr2	1.413	0.988	0.488	0.0E+00	0.0E+00
	Sdc3	1.412	0.957	0.559	0.0E+00	0.0E+00
	Npc2	1.410	1.000	0.886	0.0E+00	0.0E+00
	Dhrs3	1.394	0.995	0.661	0.0E+00	0.0E+00
	Serpine1	1.356	0.860	0.281	0.0E+00	0.0E+00
	Syngr1	1.354	0.971	0.762	0.0E+00	0.0E+00
	Atp13a2	1.353	0.978	0.468	0.0E+00	0.0E+00
	Tcf7l2	1.342	0.915	0.455	0.0E+00	0.0E+00
	Sort1	1.301	0.946	0.559	0.0E+00	0.0E+00
	Lrpap1	1.294	0.948	0.577	0.0E+00	0.0E+00
	Bhlhe41	1.284	0.943	0.552	0.0E+00	0.0E+00
	AU020206	1.254	0.941	0.692	0.0E+00	0.0E+00
**Neu-4 classic**	Retnlg	4.320	0.993	0.616	0.0E+00	0.0E+00
	Ifitm6	3.584	0.992	0.450	0.0E+00	0.0E+00
	Lcn2	3.566	0.999	0.596	0.0E+00	0.0E+00
	Wfdc21	3.439	0.999	0.640	0.0E+00	0.0E+00
	Mmp8	2.882	0.971	0.602	0.0E+00	0.0E+00
	Wfdc17	2.808	0.997	0.771	0.0E+00	0.0E+00
	Ifitm1	2.636	0.992	0.728	0.0E+00	0.0E+00
	Lrg1	2.580	0.992	0.687	0.0E+00	0.0E+00
	Prok2	2.540	0.960	0.568	0.0E+00	0.0E+00
	Ly6g	2.372	0.944	0.430	0.0E+00	0.0E+00
	Slpi	1.882	0.978	0.632	0.0E+00	0.0E+00
	Ifitm3	1.752	0.989	0.691	0.0E+00	0.0E+00
	Stfa2	1.639	0.909	0.508	0.0E+00	0.0E+00
	Cd177	1.499	0.907	0.482	0.0E+00	0.0E+00
	Chil1	1.440	0.942	0.477	0.0E+00	0.0E+00
	Anxa1	1.274	0.997	0.792	0.0E+00	0.0E+00
	Steap4	1.122	0.940	0.470	0.0E+00	0.0E+00
	Ggt1	1.101	0.897	0.480	0.0E+00	0.0E+00
	Il1f9	0.835	0.940	0.545	3.3E-294	4.9E-291
	Slfn4	0.805	0.912	0.554	3.6E-286	5.4E-283
	Tgm1	0.511	0.865	0.592	3.6E-260	5.4E-257
	Ngp	1.375	0.786	0.348	1.6E-249	2.3E-246
	Gm5483	1.224	0.916	0.606	2.1E-249	3.2E-246
	Gbp2	0.381	0.899	0.640	3.9E-247	5.9E-244
	BC100530	2.269	0.877	0.550	4.7E-247	7.0E-244
	Tgfbi	0.991	0.922	0.598	1.6E-246	2.5E-243
	Flna	0.907	0.875	0.483	5.7E-215	8.5E-212
	Csf2rb	0.767	0.972	0.676	1.4E-214	2.1E-211
	Hacd4	1.020	0.822	0.508	3.9E-214	5.8E-211
	Ccl6	0.890	0.959	0.624	1.5E-213	2.3E-210
	Gyg	0.741	0.863	0.647	1.3E-200	2.0E-197
	Stfa2l1	1.303	0.835	0.467	2.0E-193	2.9E-190
	Pi16	0.851	0.785	0.460	4.3E-188	6.4E-185
	Tceal9	0.260	0.937	0.666	1.7E-185	2.6E-182
	Id1	0.309	0.894	0.685	8.3E-174	1.2E-170
	Syne1	0.622	0.810	0.599	1.2E-162	1.8E-159
	F630028O10Ri	0.753	0.828	0.534	8.0E-158	1.2E-154
	Mgst1	0.476	0.870	0.599	2.7E-148	4.1E-145
	Gadd45a	0.597	0.832	0.616	9.7E-141	1.5E-137
	Tuba1a	0.562	0.780	0.518	2.4E-137	3.7E-134
	Napsa	0.305	0.855	0.579	6.0E-121	9.0E-118
	Adam8	0.628	0.807	0.586	3.7E-118	5.5E-115
	Ly6c2	0.635	0.708	0.387	1.9E-116	2.9E-113
	Arhgap25	0.285	0.808	0.494	8.0E-109	1.2E-105
	Fgd4	0.382	0.790	0.629	7.6E-99	1.1E-95
	Lmo4	0.595	0.715	0.404	6.3E-96	9.5E-93
	Smpdl3a	0.448	0.826	0.623	7.2E-91	1.1E-87
	Itgb2	0.339	0.867	0.585	4.4E-86	6.6E-83
	Acvrl1	0.628	0.673	0.488	1.1E-76	1.7E-73
	Saa3	0.352	0.854	0.617	1.3E-74	2.0E-71
	Sirpa	0.296	0.858	0.617	5.0E-72	7.5E-69
	Olfm4	1.468	0.683	0.470	6.3E-67	9.5E-64
	Asprv1	0.875	0.706	0.585	2.1E-58	3.2E-55

	Mt2	1.490	0.817	0.645	2.6E-238	3.8E-235
	Ccl6	2.295	0.913	0.628	1.9E-236	2.9E-233
	Cd63	2.069	0.968	0.778	4.8E-228	7.2E-225
	Apoe	1.530	0.947	0.782	7.2E-227	1.1E-223
	Chil3	2.328	0.881	0.677	1.7E-224	2.5E-221
	C1qc	1.093	0.861	0.644	9.6E-215	1.4E-211
	Lgmn	1.340	0.917	0.625	6.9E-209	1.0E-205
	Plin2	1.944	0.957	0.847	9.8E-201	1.5E-197
	Mfge8	2.225	0.887	0.675	2.6E-200	3.9E-197
	Gngt2	2.023	0.880	0.639	1.5E-198	2.2E-195
	Cd36	1.210	0.816	0.487	1.8E-196	2.6E-193
	Bhlhe41	1.075	0.805	0.561	8.0E-189	1.2E-185
	Syngr1	1.161	0.875	0.768	9.8E-184	1.5E-180
	Mt1	2.231	0.865	0.772	4.2E-183	6.3E-180
	Psap	1.901	0.939	0.754	5.6E-181	8.4E-178
	Comt	1.663	0.818	0.624	8.2E-180	1.2E-176
	Ucp2	1.750	0.878	0.729	1.1E-168	1.6E-165
	Ctsz	1.495	0.959	0.791	1.0E-166	1.5E-163
	Vat1	1.214	0.850	0.651	4.9E-166	7.4E-163
	Ctss	1.571	0.856	0.658	2.9E-164	4.3E-161
	Rps2	1.322	0.923	0.894	6.8E-152	1.0E-148
	Mgll	1.555	0.775	0.599	2.6E-149	3.9E-146
	Lipa	1.354	0.858	0.524	4.6E-147	6.9E-144
	Nme1	1.536	0.864	0.712	2.6E-139	3.8E-136
	Npc2	1.668	0.952	0.889	1.9E-138	2.9E-135
	Cd68	1.715	0.860	0.710	4.7E-137	7.0E-134
	Ndufc2	1.120	0.806	0.620	1.0E-130	1.6E-127
	Ctsd	1.460	0.982	0.965	5.3E-126	8.0E-123
	Chchd10	0.992	0.770	0.640	1.8E-116	2.7E-113
	Acp5	1.333	0.859	0.610	1.3E-111	2.0E-108
	Blvra	1.204	0.765	0.688	3.1E-107	4.6E-104
	Ckb	1.006	0.730	0.512	4.7E-105	7.1E-102
	Lrpap1	1.029	0.784	0.587	1.6E-100	2.4E-97
	Marco	1.092	0.638	0.382	1.4E-99	2.1E-96
	Hebp1	1.673	0.752	0.647	4.2E-98	6.3E-95
	Mrc1	1.060	0.678	0.421	4.6E-96	6.8E-93
	Creg1	1.335	0.866	0.665	2.1E-95	3.1E-92
	Mpeg1	1.104	0.872	0.615	1.0E-91	1.5E-88
	Rpl3	1.021	0.848	0.712	2.1E-91	3.2E-88
	Ear2	1.521	0.627	0.320	7.5E-87	1.1E-83
	Gpx1	1.184	0.891	0.835	1.3E-82	2.0E-79
	Ctsl	1.931	0.937	0.845	3.9E-82	5.8E-79
	Anxa4	1.516	0.722	0.503	2.2E-78	3.2E-75
	Gyg	1.106	0.792	0.651	6.1E-76	9.1E-73
	Akr1b3	1.361	0.704	0.519	2.5E-74	3.7E-71
	Abcg1	1.286	0.825	0.655	1.8E-73	2.7E-70
	Cybb	1.621	0.701	0.520	1.8E-71	2.7E-68
	Anxa5	1.578	0.758	0.684	1.8E-70	2.7E-67
	Il18	1.245	0.662	0.512	9.3E-69	1.4E-65
	Ftl1-ps1	0.990	0.663	0.422	6.5E-67	9.7E-64
	Lamtor4	1.225	0.821	0.703	5.3E-66	7.9E-63
	Mgst1	1.663	0.691	0.607	2.6E-64	4.0E-61
	Hexa	1.098	0.839	0.699	4.3E-62	6.4E-59
	Sh3bgrl	1.604	0.671	0.556	1.4E-58	2.1E-55
	Cndp2	1.101	0.673	0.547	2.4E-57	3.5E-54
	Lmna	1.256	0.730	0.630	2.9E-50	4.4E-47
	Gstm1	1.617	0.640	0.563	2.5E-49	3.7E-46
	Ptms	1.387	0.727	0.623	3.7E-49	5.5E-46
	Sept9	1.139	0.627	0.473	6.6E-45	9.8E-42
	Lamp1	0.986	0.887	0.863	7.9E-42	1.2E-38
	Nenf	0.948	0.658	0.522	8.2E-42	1.2E-38
	Selenop	1.239	0.639	0.523	4.1E-40	6.2E-37
	Ctsb	1.179	0.945	0.945	9.1E-39	1.4E-35
	Dbi	1.285	0.650	0.649	5.4E-32	8.1E-29
	Ccl9	1.120	0.623	0.545	1.0E-31	1.5E-28
	Ctsa	0.950	0.810	0.702	4.3E-31	6.5E-28
	Camk1	1.023	0.632	0.532	6.6E-31	9.9E-28
	Hspe1	1.301	0.691	0.676	9.4E-31	1.4E-27
	Smpdl3a	0.946	0.721	0.628	2.8E-25	4.3E-22
	Pgls	1.000	0.682	0.636	5.1E-23	7.6E-20

	Olfm4	1.468	0.683	0.470	6.3E-67	9.5E-64
	Asprv1	0.875	0.706	0.585	2.1E-58	3.2E-55
	Camp	0.968	0.597	0.362	2.9E-50	4.4E-47
	Serpinb1a	0.640	0.616	0.536	9.8E-38	1.5E-34
	Cfp	0.367	0.625	0.445	1.9E-37	2.9E-34
	Stfa3	0.514	0.382	0.548	5.2E-34	7.8E-31
	Abcd2	0.264	0.621	0.524	2.9E-28	4.3E-25
	Dgat2	0.299	0.683	0.628	9.4E-26	1.4E-22
	Rab27a	0.295	0.545	0.430	4.6E-11	6.8E-08
	Osm	0.446	0.514	0.328	4.6E-06	7.0E-03

	Smpdl3a	0.946	0.721	0.628	2.8E-25	4.3E-22
	Pgls	1.000	0.682	0.636	5.1E-23	7.6E-20
	Atp5g1	1.239	0.649	0.649	5.3E-21	8.0E-18
	Trf	0.948	0.589	0.509	6.0E-18	9.0E-15
	Fam96a	0.940	0.409	0.573	6.5E-15	9.8E-12
	Ppp1r14b	1.198	0.602	0.668	1.6E-10	2.4E-07
	Rexo2	1.352	0.558	0.640	1.2E-09	1.8E-06
	Aig1	1.010	0.561	0.683	5.7E-03	1.0E+00
