# Supplementary material for: EZH2 Inhibition Promotes Tumor Immunogenicity in Lung Squamous Cell Carcinomas
Source: Cancer Res Commun. 2024 Feb 13;4(2):388–403. doi: 10.1158/2767-9764.CRC-23-0399 (PMC10863487; doi:10.1158/2767-9764.CRC-23-0399)
Supplement: Supplementary Figure 3 — shows changes in gene expression and Gene Set Enrichment Analysis in lung squamous cell carcinoma tumoroids in response to EZH2 inhibitor and interferon-gamma treatment. [file crc-23-0399-s06.pdf]

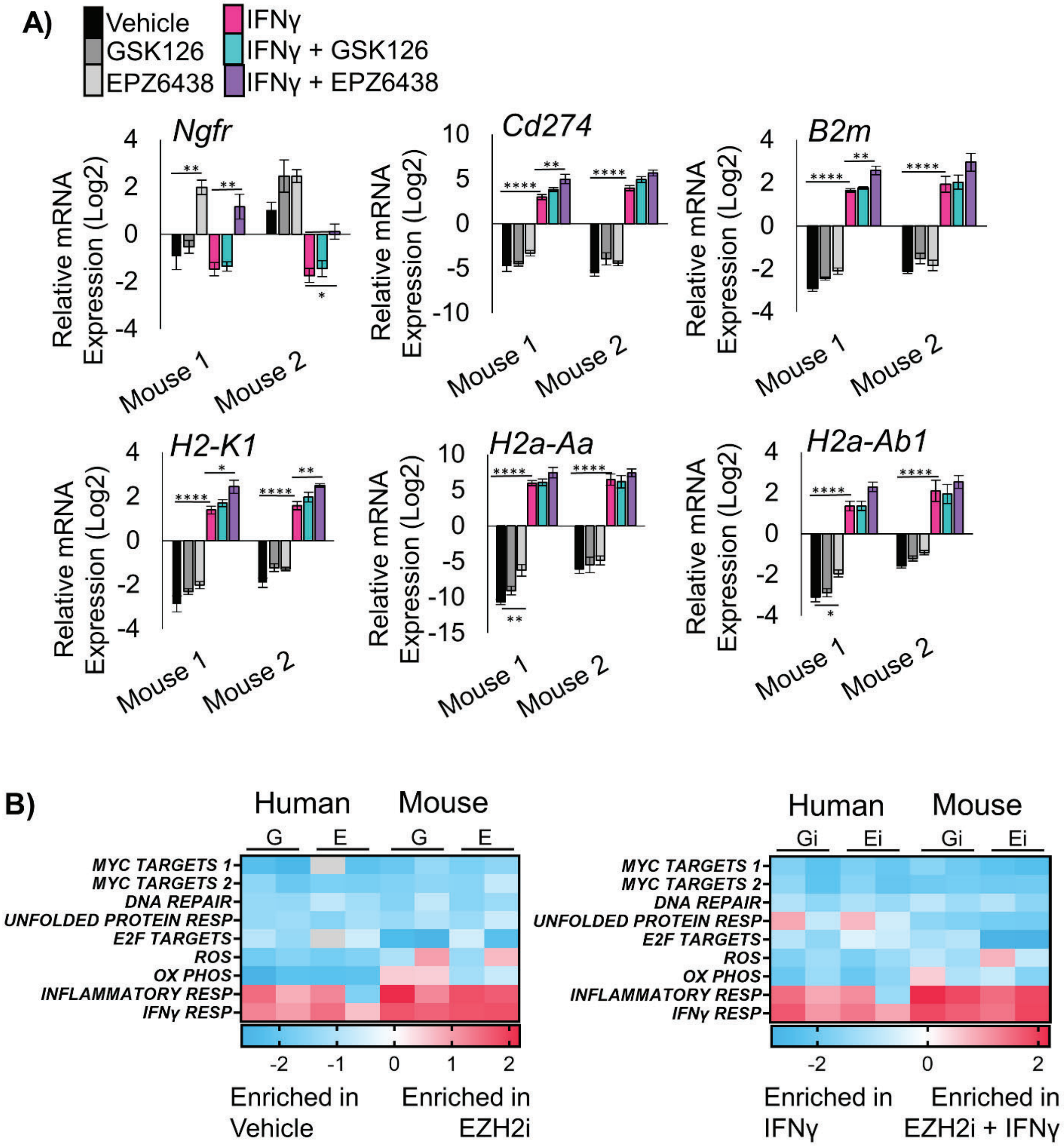

Supplementary Figure 3: Related to Figure 3

A) RT-qPCR in the indicated two unique murine tumoroid cultures treated for 11 days and IFNγ added on day 9 for the genes *Ngfr, B2m, H2-K1, Cd274,* and *H2a-Aa*, mean +/- SEM is graphed, n = 5 individual experiments for mouse 1, n=4 individual experiments for mouse 2, * indicates $p<0.05$, ** $p<0.009$, **** $p<0.0001$ by one-way ANOVA with pairwise comparisons and Holm-Šídák's *post hoc* test. **B)** Heat map of Normalized Enrichment Scores using Gene Set Enrichment Analysis on human or murine tumoroids treated with the EZH2 inhibitors GSK126 (G) or EPZ6438 (E) contrasted to vehicle control, or treated with EZH2 inhibitor and IFNγ contrasted to IFNγ alone. See also Supp. Table 2
