# Supplementary material for: EZH2 Inhibition Promotes Tumor Immunogenicity in Lung Squamous Cell Carcinomas
Source: Cancer Res Commun. 2024 Feb 13;4(2):388–403. doi: 10.1158/2767-9764.CRC-23-0399 (PMC10863487; doi:10.1158/2767-9764.CRC-23-0399)
Supplement: Supplementary Table 4 — shows Gene Set Enrichment Analysis of differentially expressed mRNAs in combination-treated tumors vs. single agent-treated tumors for three major cell sub-clusters in the single cell RNA-sequencing. [file crc-23-0399-s07.pdf]

Supplemental Table 4: GSEA on scRNAseq Populations, related to Figure 6
NES=Normalized Enrichment Score, FDR=False Discovery Rate

Group	MSigDB Signature Name	Combo vs aPD1								
		Macs/Dend			Neus			Tumor		
		NES	FDR q	-Log(q)	NES	FDR q	-Log(q)	NES	FDR q	-Log(q)
DNA Replication and Damage	REACTOME_G1_S_DNA_DAMAGE_CHECKPOINTS.v2022.1.Hs.grp	1.64	0.02	1.65	1.30	0.18	0.74	1.46	0.03	1.57
	REACTOME_ORC1_REMOVAL_FROM_CHROMATIN.v2022.1.Hs.grp	1.51	0.04	1.36	1.64	0.04	1.39	1.49	0.02	1.68
	REACTOME_SWITCHING_OF_ORIGINS_TO_A_POST_REPLICATIVE_STATE.v2022.1.Hs.grp	1.42	0.07	1.15	1.59	0.05	1.33	1.43	0.03	1.46
	REACTOME_CDT1_ASSOCIATION_WITH_THE_CDC6_ORC_ORIGIN_COMPLEX.v2022.1.Hs.grp	1.55	0.03	1.46	1.69	0.04	1.40	1.41	0.04	1.36
	REACTOME_SYNTHESIS_OF_DNA.v2022.1.Hs.grp	1.37	0.10	1.01	1.63	0.04	1.39	1.37	0.06	1.23
	REACTOME_APC_C_MEDIATED_DEGRADATION_OF_CELL_CYCLE_PROTEINS.v2022.1.Hs.grp	1.45	0.06	1.24	1.52	0.07	1.18	1.40	0.05	1.34
Protein and RNA Processing	KEGG_RIBOSOME.v2022.1.Hs.grp	2.56	0.00	4.00	1.61	0.04	1.37	-2.28	0.00	4.00
	REACTOME_EUKARYOTIC_TRANSLATION_ELONGATION.v2022.1.Hs.grp	2.56	0.00	4.00	1.52	0.07	1.17	-2.42	0.00	4.00
	Hs.grp	2.54	0.00	4.00	1.64	0.04	1.39	-2.02	0.00	2.85
	REACTOME_RESPONSE_OF_EIF2AK4_GCN2_TO_AMINO_ACID_DEFICIENCY.v2022.1.Hs.grp	2.41	0.00	4.00	1.36	0.14	0.84	-2.12	0.00	3.34
	REACTOME_EUKARYOTIC_TRANSLATION_INITIATION.v2022.1.Hs.grp	2.40	0.00	4.00	1.25	0.22	0.65	-2.11	0.00	3.37
	REACTOME_INFLUENZA_INFECTION.v2022.1.Hs.grp	2.40	0.00	4.00	1.27	0.21	0.67	-2.19	0.00	3.84
	GOBP_CYTOPLASMIC_TRANSLATION.v2022.1.Hs.grp	2.24	0.00	4.00	1.04	0.45	0.34	-1.94	0.00	2.60
	REACTOME_TRANSLATION.v2022.1.Hs.grp	2.01	0.00	3.05	1.23	0.24	0.61	-1.62	0.02	1.69
	REACTOME_SELENOAMINO_ACID_METABOLISM.v2022.1.Hs.grp	2.38	0.00	4.00	1.51	0.07	1.17	-2.38	0.00	4.00
	REACTOME_CELLULAR_RESPONSE_TO_STARVATION.v2022.1.Hs.grp	2.30	0.00	4.00	1.04	0.45	0.35	-1.80	0.01	2.14
	REACTOME_METABOLISM_OF_AMINO_ACIDS_AND_DERIVATIVES.v2022.1.Hs.grp	1.98	0.00	2.99	1.22	0.25	0.61	-1.56	0.03	1.54
	REACTOME_NONSENSE_MEDIATED_DECAY_NMD.v2022.1.Hs.grp	2.48	0.00	4.00	1.42	0.11	0.96	-2.21	0.00	4.00
	REACTOME_RRNA_PROCESSING.v2022.1.Hs.grp	2.19	0.00	4.00	1.40	0.12	0.91	-2.01	0.00	2.79
Oxidative Phosphorylation	GOBP_AEROBIC_RESPIRATION.v2022.1.Hs.grp	1.77	0.01	2.11	2.53	0.00	4.00	1.63	0.01	2.28
	GOBP_OXIDATIVE_PHOSPHORYLATION.v2022.1.Hs.grp	1.95	0.00	2.80	2.62	0.00	4.00	1.72	0.00	2.67
	GOBP_ATP_SYNTHESIS_COUPLED_ELECTRON_TRANSPORT.v2022.1.Hs.grp	2.02	0.00	3.01	2.64	0.00	4.00	1.62	0.01	2.22
	GOBP_RESPIRATORY_ELECTRON_TRANSPORT_CHAIN.v2022.1.Hs.grp	1.89	0.00	2.60	2.48	0.00	4.00	1.67	0.00	2.42
	REACTOME_RESPIRATORY_ELECTRON_TRANSPORT_ATP_SYNTHESIS_BY_CHEMIOSMOTIC_COUPLING_AND_HEAT_PRODUCTION_BY_UNCOUPLING_PROTEINS.v2022.1.Hs.grp	1.81	0.01	2.27	2.69	0.00	4.00	1.74	0.00	2.77
	GOBP_ATP_BIOSYNTHETIC_PROCESS.v2022.1.Hs.grp	1.76	0.01	2.05	2.08	0.00	3.19	1.66	0.00	2.38
	Hs.grp	1.54	0.04	1.44	2.31	0.00	4.00	1.60	0.01	2.16
	GOBP_ELECTRON_TRANSPORT_CHAIN.v2022.1.Hs.grp	1.59	0.03	1.52	2.30	0.00	4.00	1.72	0.00	2.69
	REACTOME_RESPIRATORY_ELECTRON_TRANSPORT.v2022.1.Hs.grp	1.83	0.00	2.36	2.53	0.00	4.00	1.62	0.01	2.22
	KEGG_OXIDATIVE_PHOSPHORYLATION.v2022.1.Hs.grp	1.85	0.00	2.41	2.31	0.00	4.00	1.69	0.00	2.57
	REACTOME_COMPLEX_I_BIOGENESIS.v2022.1.Hs.grp	1.83	0.00	2.37	2.13	0.00	3.23	1.58	0.01	2.10
Myeloid Migration	GOBP_NEUTROPHIL_MIGRATION.v2022.1.Hs.grp	2.02	0.00	3.03	-0.94	0.81	0.09	1.97	0.00	4.00
	GOBP_MYELOID_LEUKOCYTE_MIGRATION.v2022.1.Hs.grp	1.99	0.00	2.98	-0.95	0.83	0.08	1.84	0.00	3.20
	GOBP_NEUTROPHIL_CHEMOTAXIS.v2022.1.Hs.grp	1.99	0.00	3.00	0.88	0.72	0.14	2.12	0.00	4.00
	GOBP_GRANULOCYTE_MIGRATION.v2022.1.Hs.grp	1.97	0.00	2.99	1.08	0.42	0.38	1.94	0.00	4.00
	GOBP_LEUKOCYTE_CHEMOTAXIS.v2022.1.Hs.grp	1.90	0.00	2.61	-0.94	0.79	0.10	1.73	0.00	2.68
	GOBP_GRANULOCYTE_CHEMOTAXIS.v2022.1.Hs.grp	1.90	0.00	2.59	1.06	0.43	0.37	2.07	0.00	4.00
	GOBP_CELL_CHEMOTAXIS.v2022.1.Hs.grp	1.89	0.00	2.61	1.05	0.44	0.35	1.72	0.00	2.68
	GOBP_LEUKOCYTE_MIGRATION.v2022.1.Hs.grp	1.81	0.01	2.26	-1.04	0.76	0.12	1.68	0.00	2.45
	GOBP_TAXIS.v2022.1.Hs.grp	1.69	0.01	1.85	1.04	0.45	0.34	1.62	0.01	2.22
Myeloid Activation	GOBP_GRANULOCYTE_ACTIVATION.v2022.1.Hs.grp	1.47	0.06	1.26	1.16	0.31	0.50	1.52	0.02	1.79
	GOBP_POSITIVE_REGULATION_OF_MYELOID_CELL_DIFFERENTIATION.v2022.1.Hs.grp	1.53	0.04	1.39	1.03	0.47	0.33	1.49	0.02	1.69
	GOBP_REGULATION_OF_MYELOID_LEUKOCYTE_MEDIATED_IMMUNITY.v2022.1.Hs.grp	0.91	0.64	0.19	-0.80	0.92	0.03	1.48	0.02	1.65
	GOBP_LEUKOCYTE_MEDIATED_CYTOTOXICITY.v2022.1.Hs.grp	1.29	0.15	0.82	-0.85	0.89	0.05	1.88	0.00	3.52
	GOBP_FC_RECEPTOR_SIGNALING_PATHWAY.v2022.1.Hs.grp	1.12	0.33	0.48	-0.99	0.80	0.09	1.39	0.05	1.30
	GOBP_REGULATION_OF_LEUKOCYTE_MEDIATED_CYTOTOXICITY.v2022.1.Hs.grp	1.16	0.28	0.55	-1.03	0.73	0.14	1.72	0.00	2.66
Inflammation	GOBP_RESPONSE_TO_CHEMOKINE.v2022.1.Hs.grp	1.75	0.01	2.02	-1.26	0.38	0.42	1.69	0.00	2.57
	KEGG_CYTOKINE_CYTOKINE_RECEPTOR_INTERACTION.v2022.1.Hs.grp	1.56	0.04	1.46	-1.40	0.24	0.62	1.52	0.02	1.79
	HALLMARK_IL6_JAK_STAT3_SIGNALING.v2022.1.Hs.grp	1.55	0.04	1.45	-1.48	0.17	0.77	1.48	0.02	1.63
	GOBP_POSITIVE_REGULATION_OF_TUMOR_NECROSIS_FACTOR_SUPERFAMILY_CYTOKINE_PRODUCTION.v2022.1.Hs.grp	1.57	0.03	1.47	-1.26	0.37	0.44	1.59	0.01	2.12
	GOBP_NEGATIVE_REGULATION_OF_VIRAL_GENOME_REPLICATION.v2022.1.Hs.grp	1.93	0.00	2.72	1.53	0.06	1.20	1.35	0.07	1.17
	GOBP_TUMOR_NECROSIS_FACTOR_SUPERFAMILY_CYTOKINE_PRODUCTION.v2022.1.Hs.grp	1.81	0.01	2.26	0.97	0.56	0.25	1.56	0.01	1.98
	HALLMARK_TNFA_SIGNALING_VIA_NFKB.v2022.1.Hs.grp	1.61	0.03	1.58	-2.35	0.00	4.00	1.44	0.03	1.49
	REACTOME_INTERFERON_ALPHA_BETA_SIGNALING.v2022.1.Hs.grp	1.96	0.00	2.88	1.87	0.01	2.14	1.58	0.01	2.09
	HALLMARK_INTERFERON_ALPHA_RESPONSE.v2022.1.Hs.grp	2.17	0.00	3.82	1.79	0.02	1.76	1.57	0.01	2.06
	HALLMARK_INTERFERON_GAMMA_RESPONSE.v2022.1.Hs.grp	2.04	0.00	3.09	0.92	0.64	0.19	1.47	0.03	1.60
	GOBP_ACUTE_INFLAMMATORY_RESPONSE.v2022.1.Hs.grp	2.02	0.00	2.99	1.41	0.12	0.93	1.76	0.00	2.79
	HALLMARK_ALLOGRAFT_REJECTION.v2022.1.Hs.grp	1.72	0.01	1.92	-0.92	0.80	0.10	1.59	0.01	2.11
	HALLMARK_INFLAMMATORY_RESPONSE.v2022.1.Hs.grp	1.97	0.00	2.96	-1.51	0.15	0.82	1.49	0.02	1.67

Supplemental Table 4 Continued: GSEA on scRNAseq Populations, related to Figure 6
NES=Normalized Enrichment Score, FDR=False Discovery Rate

		Combo vs GSK								
		Macs/Dend			Neus			Tumor		
Group	MSigDB Signature Name	NES	FDR q	-Log(q)	NES	FDR q	-Log(q)	NES	FDR q	-Log(q)
DNA Replication and Damage	REACTOME_G1_S_DNA_DAMAGE_CHECKPOINTS.v2022.1.Hs.grp	1.72	0.01	2.20	1.09	0.39		1.30	0.12	0.92
	REACTOME_ORC1_REMOVAL_FROM_CHROMATIN.v2022.1.Hs.grp	1.76	0.00	2.38	1.29	0.20	0.69	1.27	0.14	0.84
	REACTOME_SWITCHING_OF_ORIGINS_TO_A_POST_REPLICATIVE_STATE.v2022.1.Hs.grp	1.65	0.01	1.96	1.20	0.26	0.58	1.23	0.18	0.75
	REACTOME_CDT1_ASSOCIATION_WITH_THE_CDC6_ORC_ORIGIN_COMPLEX.v2022.1.Hs.grp	1.76	0.00	2.37	1.30	0.20	0.69	1.21	0.19	0.71
	REACTOME_SYNTHESIS_OF_DNA.v2022.1.Hs.grp	1.62	0.01	1.84	1.18	0.29	0.54	1.20	0.20	0.69
	REACTOME_APC_C_MEDIATED_DEGRADATION_OF_CELL_CYCLE_PROTEINS.v2022.1.Hs.grp	1.66	0.01	2.00	1.22	0.25	0.60	1.20	0.20	0.69
Protein and RNA Processing	KEGG_RIBOSOME.v2022.1.Hs.grp	2.59	0.00	4.00	-1.30	0.31	0.50	-2.15	0.00	3.86
	REACTOME_EUKARYOTIC_TRANSLATION_ELONGATION.v2022.1.Hs.grp	2.61	0.00	4.00	-1.43	0.28	0.55	-2.28	0.00	4.00
	REACTOME_SRP_DEPENDENT_COTRANSLATIONAL_PROTEIN_TARGETING_TO_MEMBRANE.v2022.1.	2.51	0.00	4.00	-1.25	0.33	0.48	-2.12	0.00	3.70
	REACTOME_RESPONSE_OF_EIF2AK4_GCN2_TO_AMINO_ACID_DEFICIENCY.v2022.1.Hs.grp	2.63	0.00	4.00	-1.40	0.33	0.49	-2.27	0.00	4.00
	REACTOME_EUKARYOTIC_TRANSLATION_INITIATION.v2022.1.Hs.grp	2.52	0.00	4.00	-1.38	0.27	0.57	-2.09	0.00	3.48
	REACTOME_INFLUENZA_INFECTION.v2022.1.Hs.grp	2.53	0.00	4.00	-1.40	0.30	0.52	-2.18	0.00	3.76
	GOBP_CYTOPLASMIC_TRANSLATION.v2022.1.Hs.grp	2.41	0.00	4.00	-1.46	0.25	0.60	-2.18	0.00	3.81
	REACTOME_TRANSLATION.v2022.1.Hs.grp	2.29	0.00	4.00	-1.29	0.33	0.48	-2.02	0.00	3.36
	REACTOME_SELENOAMINO_ACID_METABOLISM.v2022.1.Hs.grp	2.56	0.00	4.00	-1.36	0.29	0.54	-2.24	0.00	4.00
	REACTOME_CELLULAR_RESPONSE_TO_STARVATION.v2022.1.Hs.grp	2.39	0.00	4.00	-1.59	0.24	0.61	-2.05	0.00	3.31
	REACTOME_METABOLISM_OF_AMINO_ACIDS_AND_DERIVATIVES.v2022.1.Hs.grp	2.21	0.00	4.00	-1.09	0.54	0.27	-1.66	0.01	1.85
	REACTOME_NONSENSE_MEDIATED_DECAY_NMD.v2022.1.Hs.grp	2.58	0.00	4.00	-1.34	0.32	0.49	-2.14	0.00	3.90
	REACTOME_RRNA_PROCESSING.v2022.1.Hs.grp	2.39	0.00	4.00	-1.38	0.28	0.55	-2.13	0.00	3.67
Oxidative Phosphorylation	GOBP_AEROBIC_RESPIRATION.v2022.1.Hs.grp	2.05	0.00	3.93	1.93	0.09	1.06	1.82	0.00	3.70
	GOBP_OXIDATIVE_PHOSPHORYLATION.v2022.1.Hs.grp	2.14	0.00	4.00	1.90	0.06	1.26	1.92	0.00	3.82
	GOBP_ATP_SYNTHESIS_COUPLED_ELECTRON_TRANSPORT.v2022.1.Hs.grp	2.14	0.00	4.00	1.83	0.06	1.23	1.88	0.00	3.97
	GOBP_RESPIRATORY_ELECTRON_TRANSPORT_CHAIN.v2022.1.Hs.grp	2.06	0.00	3.91	1.81	0.05	1.28	1.88	0.00	3.94
	REACTOME_RESPIRATORY_ELECTRON_TRANSPORT_ATP_SYNTHESIS_BY_CHEMIOSMOTIC_COUPLING_AND_HEAT_PRODUCTION_BY_UNCOUPLING_PROTEINS.v2022.1.Hs.grp	2.07	0.00	3.88	1.78	0.06	1.19	1.96	0.00	4.00
	GOBP_ATP_BIOSYNTHETIC_PROCESS.v2022.1.Hs.grp	2.08	0.00	3.85	1.72	0.06	1.24	1.98	0.00	4.00
	REACTOME_THE_CITRIC_ACID_TCA_CYCLE_AND_RESPIRATORY_ELECTRON_TRANSPORT.v2022.1.	1.96	0.00	3.45	1.72	0.05	1.26	1.87	0.00	4.04
	GOBP_ELECTRON_TRANSPORT_CHAIN.v2022.1.Hs.grp	1.84	0.00	2.70	1.71	0.05	1.26	1.88	0.00	4.19
	REACTOME_RESPIRATORY_ELECTRON_TRANSPORT.v2022.1.Hs.grp	1.95	0.00	3.41	1.70	0.05	1.30	1.91	0.00	4.04
	KEGG_OXIDATIVE_PHOSPHORYLATION.v2022.1.Hs.grp	2.07	0.00	3.87	1.65	0.06	1.21	2.05	0.00	4.00
	REACTOME_COMPLEX_I_BIOGENESIS.v2022.1.Hs.grp	1.85	0.00	2.77	1.58	0.08	1.09	1.80	0.00	3.38
Myeloid Migration	GOBP_NEUTROPHIL_MIGRATION.v2022.1.Hs.grp	1.81	0.00	2.59	1.43	0.14	0.84	1.83	0.00	3.72
	GOBP_MYELOID_LEUKOCYTE_MIGRATION.v2022.1.Hs.grp	1.88	0.00	2.95	1.38	0.17	0.78	1.84	0.00	3.72
	GOBP_NEUTROPHIL_CHEMOTAXIS.v2022.1.Hs.grp	1.84	0.00	2.70	1.26	0.21	0.68	1.75	0.00	3.07
	GOBP_GRANULOCYTE_MIGRATION.v2022.1.Hs.grp	1.89	0.00	2.99	1.54	0.09	1.03	1.75	0.00	3.07
	GOBP_LEUKOCYTE_CHEMOTAXIS.v2022.1.Hs.grp	1.91	0.00	3.14	1.35	0.17	0.76	1.51	0.02	1.72
	GOBP_GRANULOCYTE_CHEMOTAXIS.v2022.1.Hs.grp	1.90	0.00	3.05	1.39	0.16	0.79	1.71	0.00	2.84
	GOBP_CELL_CHEMOTAXIS.v2022.1.Hs.grp	1.98	0.00	3.60	1.58	0.08	1.11	1.64	0.00	2.41
	GOBP_LEUKOCYTE_MIGRATION.v2022.1.Hs.grp	1.80	0.00	2.54	1.43	0.14	0.84	1.68	0.00	2.68
	GOBP_TAXIS.v2022.1.Hs.grp	1.76	0.00	2.38	1.50	0.12	0.91	1.62	0.01	2.29
Myeloid Activation	GOBP_GRANULOCYTE_ACTIVATION.v2022.1.Hs.grp	1.57	0.02	1.66	1.55	0.09	1.03	1.73	0.00	3.01
	GOBP_POSITIVE_REGULATION_OF_MYELOID_CELL_DIFFERENTIATION.v2022.1.Hs.grp	1.29	0.15	0.83	1.08	0.39	0.41	1.76	0.00	3.16
	GOBP_REGULATION_OF_MYELOID_LEUKOCYTE_MEDIATED_IMMUNITY.v2022.1.Hs.grp	1.39	0.08	1.10	1.19	0.28	0.55	1.79	0.00	3.35
	GOBP_LEUKOCYTE_MEDIATED_CYTOTOXICITY.v2022.1.Hs.grp	1.35	0.10	1.00	0.91	0.65	0.19	1.72	0.00	2.95
	GOBP_FC_RECEPTOR_SIGNALING_PATHWAY.v2022.1.Hs.grp	1.39	0.08	1.10	0.94	0.60	0.22	1.77	0.00	3.19
	GOBP_REGULATION_OF_LEUKOCYTE_MEDIATED_CYTOTOXICITY.v2022.1.Hs.grp	1.12	0.31	0.50	-0.69	0.99	0.00	1.79	0.00	3.39
Inflammation	GOBP_RESPONSE_TO_CHEMOKINE.v2022.1.Hs.grp	1.61	0.02	1.82	-0.90	0.79	0.10	1.73	0.00	3.00
	KEGG_CYTOKINE_CYTOKINE_RECEPTOR_INTERACTION.v2022.1.Hs.grp	1.62	0.01	1.83	0.84	0.78	0.11	1.64	0.00	2.37
	HALLMARK_IL6_JAK_STAT3_SIGNALING.v2022.1.Hs.grp	1.67	0.01	2.02	0.66	0.96	0.02	1.50	0.02	1.66
	GOBP_POSITIVE_REGULATION_OF_TUMOR_NECROSIS_FACTOR_SUPERFAMILY_CYTOKINE_PRODUCTION.v2022.1.Hs.grp	1.24	0.18	0.75	-1.12	0.48	0.32	1.89	0.00	4.15
	GOBP_NEGATIVE_REGULATION_OF_VIRAL_GENOME_REPLICATION.v2022.1.Hs.grp	1.52	0.03	1.51	1.66	0.06	1.26	1.83	0.00	3.70
	GOBP_TUMOR_NECROSIS_FACTOR_SUPERFAMILY_CYTOKINE_PRODUCTION.v2022.1.Hs.grp	1.38	0.08	1.09	-0.87	0.83	0.08	1.77	0.00	3.17
	HALLMARK_TNFA_SIGNALING_VIA_NFKB.v2022.1.Hs.grp	1.65	0.01	1.96	-1.33	0.32	0.49	1.38	0.06	1.20
	REACTOME_INTERFERON_ALPHA_BETA_SIGNALING.v2022.1.Hs.grp	1.67	0.01	2.01	1.12	0.35	0.46	1.73	0.00	3.01
	HALLMARK_INTERFERON_ALPHA_RESPONSE.v2022.1.Hs.grp	1.83	0.00	2.69	1.43	0.15	0.84	1.84	0.00	3.74
	HALLMARK_INTERFERON_GAMMA_RESPONSE.v2022.1.Hs.grp	1.98	0.00	3.57	1.30	0.20	0.70	1.78	0.00	3.28
	GOBP_ACUTE_INFLAMMATORY_RESPONSE.v2022.1.Hs.grp	1.85	0.00	2.74	1.68	0.06	1.24	1.71	0.00	2.83
	HALLMARK_ALLOGRAFT_REJECTION.v2022.1.Hs.grp	1.94	0.00	3.33	0.96	0.57	0.24	1.53	0.02	1.78
	HALLMARK_INFLAMMATORY_RESPONSE.v2022.1.Hs.grp	1.82	0.00	2.66	1.07	0.40	0.39	1.71	0.00	2.84
