# Supplementary material for: EZH2 Inhibition Promotes Tumor Immunogenicity in Lung Squamous Cell Carcinomas
Source: Cancer Res Commun. 2024 Feb 13;4(2):388–403. doi: 10.1158/2767-9764.CRC-23-0399 (PMC10863487; doi:10.1158/2767-9764.CRC-23-0399)
Supplement: Supplementary Figure 4 — shows additional ChIP-sequencing data from human lung squamous cell carcinoma tumoroids. [file crc-23-0399-s08.pdf]

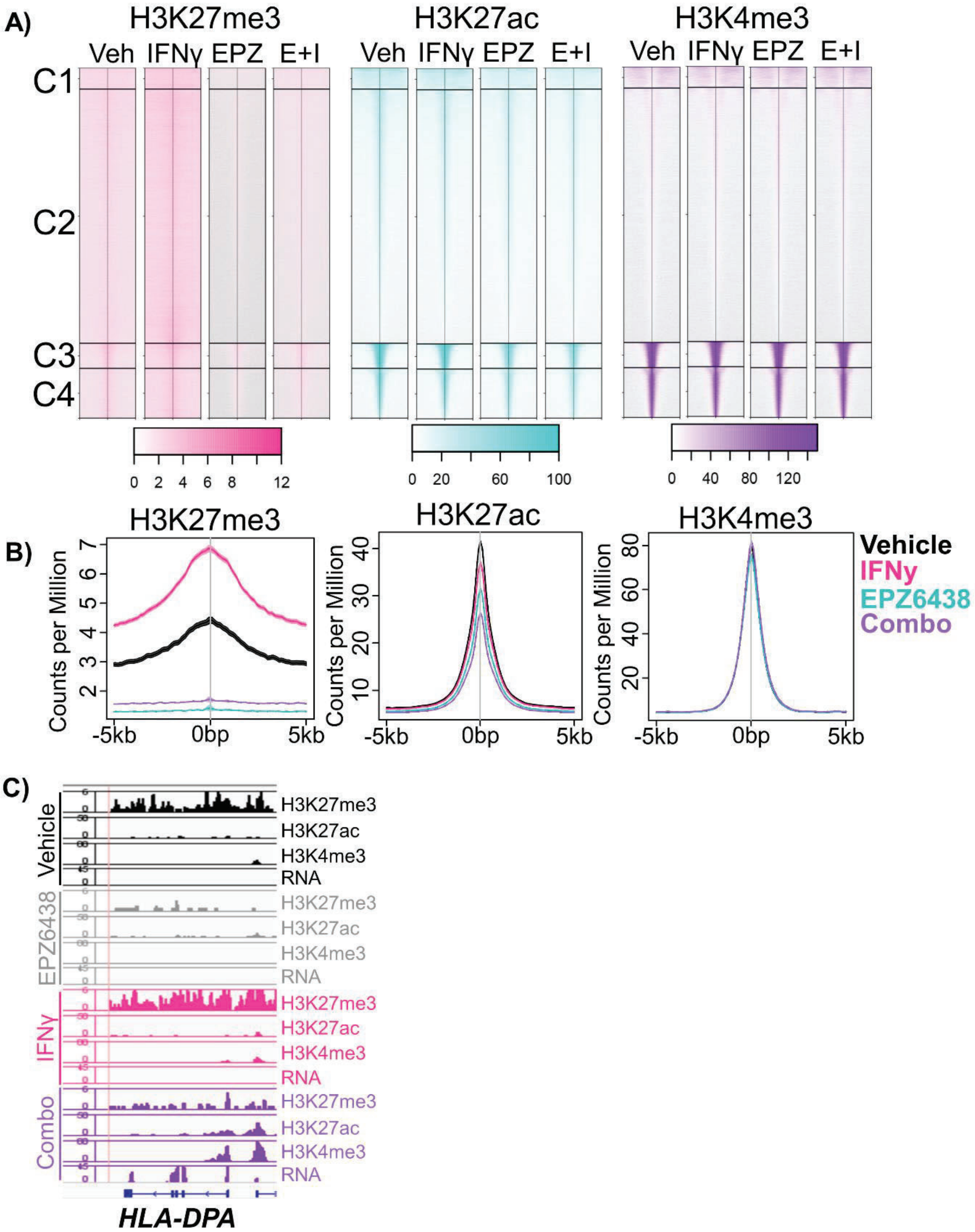

Supplementary Figure 4: Related to Figure 4

A) Heatmap representation of H3K27me3-, H3K27ac- and H3K4me3-bound chromatin peaks centered across a ±5kb window that shows occupancy in tumoroids cultures of the indicated treatments. **B)** Histogram of merged ChIP-seq peaks in the indicated 3D tumoroid samples. **C)** Wiggle plots for H3K27me3, H3K27ac, and H3K4me3 histone mark enrichments, and matched RNAseq tracks in patient-derived tumoroids from the indicated treatment groups for the gene *HLA-DPA*.
