# Supplementary material for: EZH2 Inhibition Promotes Tumor Immunogenicity in Lung Squamous Cell Carcinomas
Source: Cancer Res Commun. 2024 Feb 13;4(2):388–403. doi: 10.1158/2767-9764.CRC-23-0399 (PMC10863487; doi:10.1158/2767-9764.CRC-23-0399)
Supplement: Supplementary Table 5 — shows Gene Set Enrichment Analysis of differentially expressed mRNAs in therapy-treated tumors vs. placebo-treated tumors for three major cell sub-clusters in the single cell RNA-sequencing. [file crc-23-0399-s09.pdf]

Supplemental Table 4 Continued: GSEA on scRNAseq Populations, related to Figure 6
NES=Normalized Enrichment Score, FDR=False Discovery Rate

		Combo vs GSK								
		Macs/Dend			Neus			Tumor		
Group	MSigDB Signature Name	NES	FDR q	-Log(q)	NES	FDR q	-Log(q)	NES	FDR q	-Log(q)
DNA Replication and Damage	REACTOME_G1_S_DNA_DAMAGE_CHECKPOINTS.v2022.1.Hs.grp	1.72	0.01	2.20	1.09	0.39		1.30	0.12	0.92
	REACTOME_ORC1_REMOVAL_FROM_CHROMATIN.v2022.1.Hs.grp	1.76	0.00	2.38	1.29	0.20	0.69	1.27	0.14	0.84
	REACTOME_SWITCHING_OF_ORIGINS_TO_A_POST_REPLICATIVE_STATE.v2022.1.Hs.grp	1.65	0.01	1.96	1.20	0.26	0.58	1.23	0.18	0.75
	REACTOME_CDT1_ASSOCIATION_WITH_THE_CDC6_ORC_ORIGIN_COMPLEX.v2022.1.Hs.grp	1.76	0.00	2.37	1.30	0.20	0.69	1.21	0.19	0.71
	REACTOME_SYNTHESIS_OF_DNA.v2022.1.Hs.grp	1.62	0.01	1.84	1.18	0.29	0.54	1.20	0.20	0.69
	REACTOME_APC_C_MEDIATED_DEGRADATION_OF_CELL_CYCLE_PROTEINS.v2022.1.Hs.grp	1.66	0.01	2.00	1.22	0.25	0.60	1.20	0.20	0.69
Protein and RNA Processing	KEGG_RIBOSOME.v2022.1.Hs.grp	2.59	0.00	4.00	-1.30	0.31	0.50	-2.15	0.00	3.86
	REACTOME_EUKARYOTIC_TRANSLATION_ELONGATION.v2022.1.Hs.grp	2.61	0.00	4.00	-1.43	0.28	0.55	-2.28	0.00	4.00
	REACTOME_SRP_DEPENDENT_COTRANSLATIONAL_PROTEIN_TARGETING_TO_MEMBRANE.v2022.1.	2.51	0.00	4.00	-1.25	0.33	0.48	-2.12	0.00	3.70
	REACTOME_RESPONSE_OF_EIF2AK4_GCN2_TO_AMINO_ACID_DEFICIENCY.v2022.1.Hs.grp	2.63	0.00	4.00	-1.40	0.33	0.49	-2.27	0.00	4.00
	REACTOME_EUKARYOTIC_TRANSLATION_INITIATION.v2022.1.Hs.grp	2.52	0.00	4.00	-1.38	0.27	0.57	-2.09	0.00	3.48
	REACTOME_INFLUENZA_INFECTION.v2022.1.Hs.grp	2.53	0.00	4.00	-1.40	0.30	0.52	-2.18	0.00	3.76
	GOBP_CYTOPLASMIC_TRANSLATION.v2022.1.Hs.grp	2.41	0.00	4.00	-1.46	0.25	0.60	-2.18	0.00	3.81
	REACTOME_TRANSLATION.v2022.1.Hs.grp	2.29	0.00	4.00	-1.29	0.33	0.48	-2.02	0.00	3.36
	REACTOME_SELENOAMINO_ACID_METABOLISM.v2022.1.Hs.grp	2.56	0.00	4.00	-1.36	0.29	0.54	-2.24	0.00	4.00
	REACTOME_CELLULAR_RESPONSE_TO_STARVATION.v2022.1.Hs.grp	2.39	0.00	4.00	-1.59	0.24	0.61	-2.05	0.00	3.31
	REACTOME_METABOLISM_OF_AMINO_ACIDS_AND_DERIVATIVES.v2022.1.Hs.grp	2.21	0.00	4.00	-1.09	0.54	0.27	-1.66	0.01	1.85
	REACTOME_NONSENSE_MEDIATED_DECAY_NMD.v2022.1.Hs.grp	2.58	0.00	4.00	-1.34	0.32	0.49	-2.14	0.00	3.90
	REACTOME_RRNA_PROCESSING.v2022.1.Hs.grp	2.39	0.00	4.00	-1.38	0.28	0.55	-2.13	0.00	3.67
Oxidative Phosphorylation	GOBP_AEROBIC_RESPIRATION.v2022.1.Hs.grp	2.05	0.00	3.93	1.93	0.09	1.06	1.82	0.00	3.70
	GOBP_OXIDATIVE_PHOSPHORYLATION.v2022.1.Hs.grp	2.14	0.00	4.00	1.90	0.06	1.26	1.92	0.00	3.82
	GOBP_ATP_SYNTHESIS_COUPLED_ELECTRON_TRANSPORT.v2022.1.Hs.grp	2.14	0.00	4.00	1.83	0.06	1.23	1.88	0.00	3.97
	GOBP_RESPIRATORY_ELECTRON_TRANSPORT_CHAIN.v2022.1.Hs.grp	2.06	0.00	3.91	1.81	0.05	1.28	1.88	0.00	3.94
	REACTOME_RESPIRATORY_ELECTRON_TRANSPORT_ATP_SYNTHESIS_BY_CHEMIOSMOTIC_COUPLING_AND_HEAT_PRODUCTION_BY_UNCOUPLING_PROTEINS.v2022.1.Hs.grp	2.07	0.00	3.88	1.78	0.06	1.19	1.96	0.00	4.00
	GOBP_ATP_BIOSYNTHETIC_PROCESS.v2022.1.Hs.grp	2.08	0.00	3.85	1.72	0.06	1.24	1.98	0.00	4.00
	REACTOME_THE_CITRIC_ACID_TCA_CYCLE_AND_RESPIRATORY_ELECTRON_TRANSPORT.v2022.1.	1.96	0.00	3.45	1.72	0.05	1.26	1.87	0.00	4.04
	GOBP_ELECTRON_TRANSPORT_CHAIN.v2022.1.Hs.grp	1.84	0.00	2.70	1.71	0.05	1.26	1.88	0.00	4.19
	REACTOME_RESPIRATORY_ELECTRON_TRANSPORT.v2022.1.Hs.grp	1.95	0.00	3.41	1.70	0.05	1.30	1.91	0.00	4.04
	KEGG_OXIDATIVE_PHOSPHORYLATION.v2022.1.Hs.grp	2.07	0.00	3.87	1.65	0.06	1.21	2.05	0.00	4.00
	REACTOME_COMPLEX_I_BIOGENESIS.v2022.1.Hs.grp	1.85	0.00	2.77	1.58	0.08	1.09	1.80	0.00	3.38
Myeloid Migration	GOBP_NEUTROPHIL_MIGRATION.v2022.1.Hs.grp	1.81	0.00	2.59	1.43	0.14	0.84	1.83	0.00	3.72
	GOBP_MYELOID_LEUKOCYTE_MIGRATION.v2022.1.Hs.grp	1.88	0.00	2.95	1.38	0.17	0.78	1.84	0.00	3.72
	GOBP_NEUTROPHIL_CHEMOTAXIS.v2022.1.Hs.grp	1.84	0.00	2.70	1.26	0.21	0.68	1.75	0.00	3.07
	GOBP_GRANULOCYTE_MIGRATION.v2022.1.Hs.grp	1.89	0.00	2.99	1.54	0.09	1.03	1.75	0.00	3.07
	GOBP_LEUKOCYTE_CHEMOTAXIS.v2022.1.Hs.grp	1.91	0.00	3.14	1.35	0.17	0.76	1.51	0.02	1.72
	GOBP_GRANULOCYTE_CHEMOTAXIS.v2022.1.Hs.grp	1.90	0.00	3.05	1.39	0.16	0.79	1.71	0.00	2.84
	GOBP_CELL_CHEMOTAXIS.v2022.1.Hs.grp	1.98	0.00	3.60	1.58	0.08	1.11	1.64	0.00	2.41
	GOBP_LEUKOCYTE_MIGRATION.v2022.1.Hs.grp	1.80	0.00	2.54	1.43	0.14	0.84	1.68	0.00	2.68
	GOBP_TAXIS.v2022.1.Hs.grp	1.76	0.00	2.38	1.50	0.12	0.91	1.62	0.01	2.29
Myeloid Activation	GOBP_GRANULOCYTE_ACTIVATION.v2022.1.Hs.grp	1.57	0.02	1.66	1.55	0.09	1.03	1.73	0.00	3.01
	GOBP_POSITIVE_REGULATION_OF_MYELOID_CELL_DIFFERENTIATION.v2022.1.Hs.grp	1.29	0.15	0.83	1.08	0.39	0.41	1.76	0.00	3.16
	GOBP_REGULATION_OF_MYELOID_LEUKOCYTE_MEDIATED_IMMUNITY.v2022.1.Hs.grp	1.39	0.08	1.10	1.19	0.28	0.55	1.79	0.00	3.35
	GOBP_LEUKOCYTE_MEDIATED_CYTOTOXICITY.v2022.1.Hs.grp	1.35	0.10	1.00	0.91	0.65	0.19	1.72	0.00	2.95
	GOBP_FC_RECEPTOR_SIGNALING_PATHWAY.v2022.1.Hs.grp	1.39	0.08	1.10	0.94	0.60	0.22	1.77	0.00	3.19
	GOBP_REGULATION_OF_LEUKOCYTE_MEDIATED_CYTOTOXICITY.v2022.1.Hs.grp	1.12	0.31	0.50	-0.69	0.99	0.00	1.79	0.00	3.39
Inflammation	GOBP_RESPONSE_TO_CHEMOKINE.v2022.1.Hs.grp	1.61	0.02	1.82	-0.90	0.79	0.10	1.73	0.00	3.00
	KEGG_CYTOKINE_CYTOKINE_RECEPTOR_INTERACTION.v2022.1.Hs.grp	1.62	0.01	1.83	0.84	0.78	0.11	1.64	0.00	2.37
	HALLMARK_IL6_JAK_STAT3_SIGNALING.v2022.1.Hs.grp	1.67	0.01	2.02	0.66	0.96	0.02	1.50	0.02	1.66
	GOBP_POSITIVE_REGULATION_OF_TUMOR_NECROSIS_FACTOR_SUPERFAMILY_CYTOKINE_PRODUCTION.v2022.1.Hs.grp	1.24	0.18	0.75	-1.12	0.48	0.32	1.89	0.00	4.15
	GOBP_NEGATIVE_REGULATION_OF_VIRAL_GENOME_REPLICATION.v2022.1.Hs.grp	1.52	0.03	1.51	1.66	0.06	1.26	1.83	0.00	3.70
	GOBP_TUMOR_NECROSIS_FACTOR_SUPERFAMILY_CYTOKINE_PRODUCTION.v2022.1.Hs.grp	1.38	0.08	1.09	-0.87	0.83	0.08	1.77	0.00	3.17
	HALLMARK_TNFA_SIGNALING_VIA_NFKB.v2022.1.Hs.grp	1.65	0.01	1.96	-1.33	0.32	0.49	1.38	0.06	1.20
	REACTOME_INTERFERON_ALPHA_BETA_SIGNALING.v2022.1.Hs.grp	1.67	0.01	2.01	1.12	0.35	0.46	1.73	0.00	3.01
	HALLMARK_INTERFERON_ALPHA_RESPONSE.v2022.1.Hs.grp	1.83	0.00	2.69	1.43	0.15	0.84	1.84	0.00	3.74
	HALLMARK_INTERFERON_GAMMA_RESPONSE.v2022.1.Hs.grp	1.98	0.00	3.57	1.30	0.20	0.70	1.78	0.00	3.28
	GOBP_ACUTE_INFLAMMATORY_RESPONSE.v2022.1.Hs.grp	1.85	0.00	2.74	1.68	0.06	1.24	1.71	0.00	2.83
	HALLMARK_ALLOGRAFT_REJECTION.v2022.1.Hs.grp	1.94	0.00	3.33	0.96	0.57	0.24	1.53	0.02	1.78
	HALLMARK_INFLAMMATORY_RESPONSE.v2022.1.Hs.grp	1.82	0.00	2.66	1.07	0.40	0.39	1.71	0.00	2.84

Supplemental Table 5: GSEA on scRNAseq Populations, related to Figure 6
NES=Normalized Enrichment Score, FDR=False Discovery Rate

Group	MSigDB Signature Name	Combo vs Placebo								
		Macs/Dend			Neus			Tumor		
		NES	FDR c	-Log(q)	NES	FDR q	-Log(q)	NES	FDR q	-Log(q)
DNA Replication and Damage	REACTOME_G1_S_DNA_DAMAGE_CHECKPOINTS.v2022.1.Hs.grp	2.19	0.00	4.00	1.86	0.00	2.76	1.50	0.03	1.60
	REACTOME_ORC1_REMOVAL_FROM_CHROMATIN.v2022.1.Hs.grp	2.27	0.00	4.00	1.95	0.00	3.16	1.44	0.04	1.39
	REACTOME_SWITCHING_OF_ORIGINS_TO_A_POST_REPLICATIVE_STATE.v2022.1.Hs.grp	2.18	0.00	4.00	1.95	0.00	3.16	1.42	0.05	1.33
	REACTOME_CDT1_ASSOCIATION_WITH_THE_CDC6_ORC_ORIGIN_COMPLEX.v2022.1.Hs.grp	2.22	0.00	4.00	1.88	0.00	2.86	1.47	0.03	1.52
	REACTOME_SYNTHESIS_OF_DNA.v2022.1.Hs.grp	2.06	0.00	4.01	2.01	0.00	3.50	1.39	0.06	1.23
	REACTOME_APC_C_MEDIATED_DEGRADATION_OF_CELL_CYCLE_PROTEINS.v2022.1.Hs.grp	2.18	0.00	4.00	1.96	0.00	3.18	1.47	0.03	1.52
Protein and RNA Processing	KEGG_RIBOSOME.v2022.1.Hs.grp	3.06	0.00	4.00	3.34	0.00	4.00	1.61	0.01	2.04
	REACTOME_EUKARYOTIC_TRANSLATION_ELONGATION.v2022.1.Hs.grp	3.06	0.00	4.00	3.33	0.00	4.00	1.57	0.01	1.90
	REACTOME_SRP_DEPENDENT_COTRANSLATIONAL_PROTEIN_TARGETING_TO_MEMBRANE.v2022.1.Hs.grp	3.03	0.00	4.00	3.34	0.00	4.00	1.58	0.01	1.92
	REACTOME_RESPONSE_OF_EIF2AK4_GCN2_TO_AMINO_ACID_DEFICIENCY.v2022.1.Hs.grp	3.06	0.00	4.00	3.32	0.00	4.00	1.52	0.02	1.68
	REACTOME_EUKARYOTIC_TRANSLATION_INITIATION.v2022.1.Hs.grp	2.94	0.00	4.00	3.36	0.00	4.00	1.58	0.01	1.92
	REACTOME_INFLUENZA_INFECTION.v2022.1.Hs.grp	2.89	0.00	4.00	3.23	0.00	4.00	1.33	0.09	1.02
	GOBP_CYTOPLASMIC_TRANSLATION.v2022.1.Hs.grp	2.81	0.00	4.00	3.26	0.00	4.00	1.48	0.03	1.53
	REACTOME_TRANSLATION.v2022.1.Hs.grp	2.81	0.00	4.00	3.21	0.00	4.00	1.35	0.08	1.11
	REACTOME_SELENOAMINO_ACID_METABOLISM.v2022.1.Hs.grp	3.04	0.00	4.00	3.36	0.00	4.00	1.50	0.02	1.61
	REACTOME_CELLULAR_RESPONSE_TO_STARVATION.v2022.1.Hs.grp	2.86	0.00	4.00	3.22	0.00	4.00	1.54	0.02	1.72
	REACTOME_METABOLISM_OF_AMINO_ACIDS_AND_DERIVATIVES.v2022.1.Hs.grp	2.72	0.00	4.00	3.06	0.00	4.00	1.41	0.05	1.32
	REACTOME_NONSENSE_MEDIATED_DECAY_NMD.v2022.1.Hs.grp	2.93	0.00	4.00	3.33	0.00	4.00	1.50	0.02	1.61
	REACTOME_RRNA_PROCESSING.v2022.1.Hs.grp	2.93	0.00	4.00	3.28	0.00	4.00	1.48	0.03	1.55
Oxidative Phosphorylation	GOBP_AEROBIC_RESPIRATION.v2022.1.Hs.grp	1.70	0.01	2.12	2.59	0.00	4.00	1.86	0.00	3.69
	GOBP_OXIDATIVE_PHOSPHORYLATION.v2022.1.Hs.grp	1.88	0.00	2.91	2.66	0.00	4.00	1.96	0.00	4.13
	GOBP_ATP_SYNTHESIS_COUPLED_ELECTRON_TRANSPORT.v2022.1.Hs.grp	1.83	0.00	2.73	2.54	0.00	4.00	1.92	0.00	3.99
	GOBP_RESPIRATORY_ELECTRON_TRANSPORT_CHAIN.v2022.1.Hs.grp	1.71	0.01	2.15	2.47	0.00	4.00	1.91	0.00	3.86
	REACTOME_RESPIRATORY_ELECTRON_TRANSPORT_ATP_SYNTHESIS_BY_CHEMIOSMOTIC_COUPLING_AND_HEAT_PRODUCTION_BY_UNCOUPLING_PROTEINS.v2022.1.Hs.grp	1.75	0.00	2.32	2.69	0.00	4.00	2.10	0.00	4.00
	GOBP_ATP_BIOSYNTHETIC_PROCESS.v2022.1.Hs.grp	1.84	0.00	2.73	2.45	0.00	4.00	2.06	0.00	4.00
	REACTOME_THE_CITRIC_ACID_TCA_CYCLE_AND_RESPIRATORY_ELECTRON_TRANSPORT.v2022.1.Hs.grp	1.48	0.04	1.43	2.64	0.00	4.00	1.92	0.00	3.97
	GOBP_ELECTRON_TRANSPORT_CHAIN.v2022.1.Hs.grp	1.46	0.04	1.36	2.27	0.00	4.00	1.90	0.00	3.77
	REACTOME_RESPIRATORY_ELECTRON_TRANSPORT.v2022.1.Hs.grp	1.70	0.01	2.13	2.53	0.00	4.00	2.01	0.00	3.79
	KEGG_OXIDATIVE_PHOSPHORYLATION.v2022.1.Hs.grp	1.69	0.01	2.12	2.63	0.00	4.00	2.13	0.00	4.00
	REACTOME_COMPLEX_I_BIOGENESIS.v2022.1.Hs.grp	1.82	0.00	2.63	2.20	0.00	4.00	1.90	0.00	3.75
Myeloid Migration	GOBP_NEUTROPHIL_MIGRATION.v2022.1.Hs.grp	-1.43	0.06	1.25	-1.49	0.08	1.11	1.16	0.27	0.57
	GOBP_MYELOID_LEUKOCYTE_MIGRATION.v2022.1.Hs.grp	-1.51	0.04	1.43	-1.43	0.10	1.00	1.30	0.11	0.94
	GOBP_NEUTROPHIL_CHEMOTAXIS.v2022.1.Hs.grp	-1.41	0.07	1.18	-1.50	0.07	1.13	1.20	0.22	0.66
	GOBP_GRANULOCYTE_MIGRATION.v2022.1.Hs.grp	-1.49	0.04	1.40	-1.43	0.10	0.99	1.23	0.18	0.74
	GOBP_LEUKOCYTE_CHEMOTAXIS.v2022.1.Hs.grp	-1.50	0.04	1.43	-1.76	0.01	1.84	1.09	0.37	0.43
	GOBP_GRANULOCYTE_CHEMOTAXIS.v2022.1.Hs.grp	-1.50	0.04	1.43	-1.46	0.09	1.06	1.25	0.17	0.78
	GOBP_CELL_CHEMOTAXIS.v2022.1.Hs.grp	-1.50	0.04	1.43	-1.84	0.01	2.03	1.19	0.23	0.64
	GOBP_LEUKOCYTE_MIGRATION.v2022.1.Hs.grp	-1.60	0.02	1.64	-1.78	0.01	1.89	1.13	0.31	0.51
	GOBP_TAXIS.v2022.1.Hs.grp	-1.65	0.02	1.81	-1.72	0.02	1.77	1.14	0.31	0.51
Myeloid Activation	GOBP_GRANULOCYTE_ACTIVATION.v2022.1.Hs.grp	-1.19	0.22	0.65	-1.21	0.24	0.62	1.04	0.47	0.33
	GOBP_POSITIVE_REGULATION_OF_MYELOID_CELL_DIFFERENTIATION.v2022.1.Hs.grp	-1.44	0.05	1.28	-1.80	0.01	1.91	0.75	0.88	0.06
	GOBP_REGULATION_OF_MYELOID_LEUKOCYTE_MEDIATED_IMMUNITY.v2022.1.Hs.grp	-0.84	0.78	0.11	-1.05	0.45	0.35	-1.00	0.47	0.33
	GOBP_LEUKOCYTE_MEDIATED_CYTOTOXICITY.v2022.1.Hs.grp	1.23	0.16	0.79	-1.33	0.16	0.81	1.42	0.05	1.33
	GOBP_FC_RECEPTOR_SIGNALING_PATHWAY.v2022.1.Hs.grp	-0.91	0.66	0.18	-1.51	0.07	1.14	0.67	0.93	0.03
	GOBP_REGULATION_OF_LEUKOCYTE_MEDIATED_CYTOTOXICITY.v2022.1.Hs.grp	1.22	0.17	0.77	-1.58	0.05	1.32	0.97	0.58	0.23
Inflammation	GOBP_RESPONSE_TO_CHEMOKINE.v2022.1.Hs.grp	-1.90	0.00	2.76	-2.13	0.00	4.00	-1.25	0.16	0.81
	KEGG_CYTOKINE_CYTOKINE_RECEPTOR_INTERACTION.v2022.1.Hs.grp	-1.56	0.03	1.56	-1.79	0.01	1.93	-1.97	0.00	2.45
	HALLMARK_IL6_JAK_STAT3_SIGNALING.v2022.1.Hs.grp	-1.55	0.03	1.53	-1.97	0.00	2.37	-1.15	0.24	0.61
	p	0.94	0.58	0.24	-0.99	0.52	0.28	1.38	0.06	1.19
	GOBP_NEGATIVE_REGULATION_OF_VIRAL_GENOME_REPLICATION.v2022.1.Hs.grp	1.30	0.11	0.97	1.12	0.29	0.54	-2.23	0.00	2.47
	GOBP_TUMOR_NECROSIS_FACTOR_SUPERFAMILY_CYTOKINE_PRODUCTION.v2022.1.Hs.grp	-1.04	0.43	0.37	-0.99	0.53	0.28	1.08	0.39	0.41
	HALLMARK_TNFA_SIGNALING_VIA_NFKB.v2022.1.Hs.grp	-2.36	0.00	4.00	-3.21	0.00	4.00	-1.41	0.07	1.18
	REACTOME_INTERFERON_ALPHA_BETA_SIGNALING.v2022.1.Hs.grp	1.52	0.03	1.52	1.22	0.18	0.74	-2.17	0.00	2.77
	HALLMARK_INTERFERON_ALPHA_RESPONSE.v2022.1.Hs.grp	1.44	0.05	1.34	1.39	0.08	1.11	-2.14	0.00	2.93
	HALLMARK_INTERFERON_GAMMA_RESPONSE.v2022.1.Hs.grp	1.32	0.10	1.00	-0.98	0.54	0.27	-1.44	0.06	1.21
	GOBP_ACUTE_INFLAMMATORY_RESPONSE.v2022.1.Hs.grp	1.63	0.01	1.87	-1.54	0.06	1.21	1.36	0.08	1.12
	HALLMARK_ALLOGRAFT_REJECTION.v2022.1.Hs.grp	1.36	0.08	1.11	-1.03	0.47	0.33	1.06	0.43	0.36
	HALLMARK_INFLAMMATORY_RESPONSE.v2022.1.Hs.grp	-1.55	0.03	1.52	-1.90	0.01	2.28	-1.47	0.06	1.22
