# Supplementary material for: EZH2 Inhibition Promotes Tumor Immunogenicity in Lung Squamous Cell Carcinomas
Source: Cancer Res Commun. 2024 Feb 13;4(2):388–403. doi: 10.1158/2767-9764.CRC-23-0399 (PMC10863487; doi:10.1158/2767-9764.CRC-23-0399)
Supplement: Supplementary Figure 5 — shows additional analysis of tumors from EZH2 inhibition and anti-PD1 treated mice. [file crc-23-0399-s10.pdf]

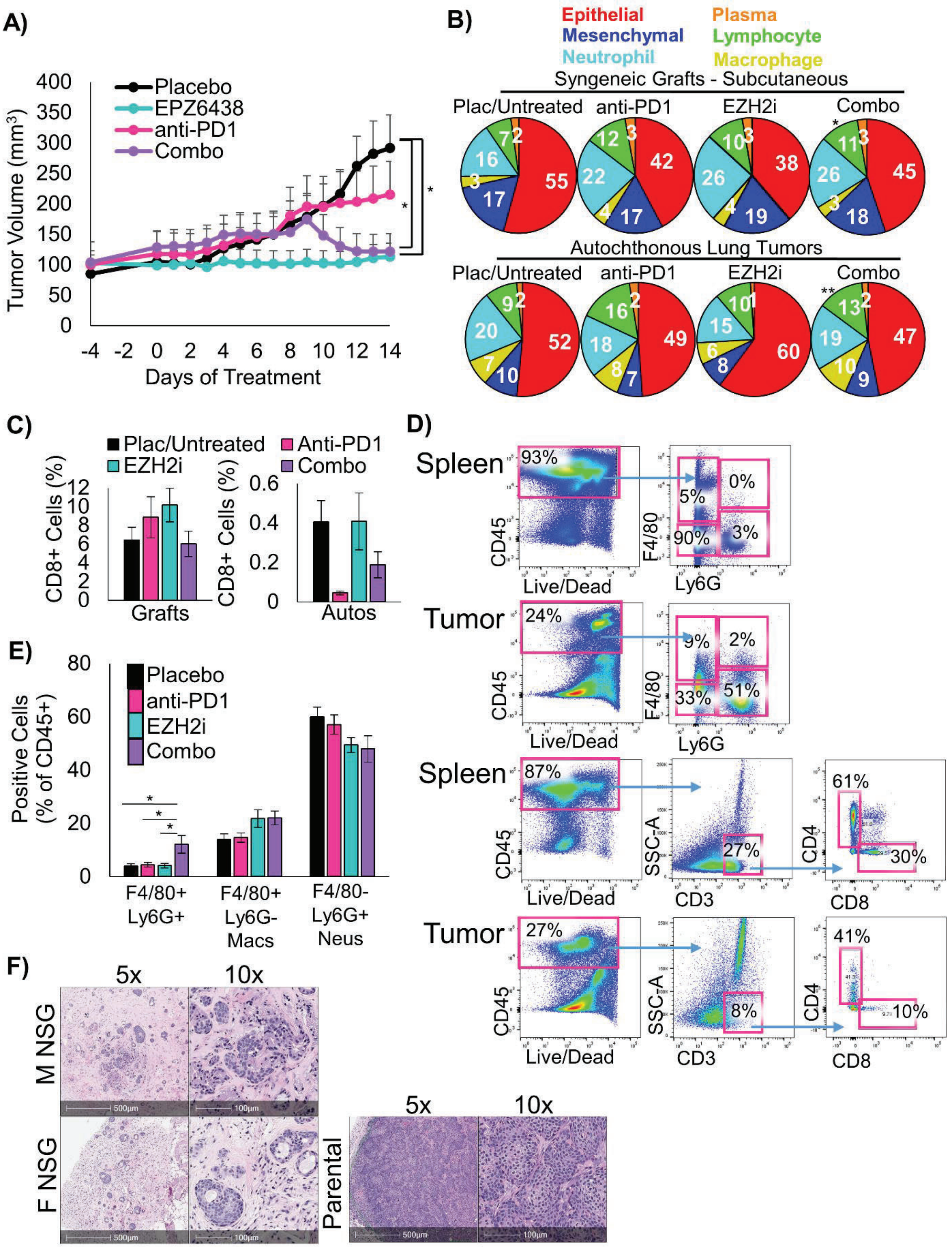

Supplementary Figure 5: Related to Figure 5

A) Tumor volume from the syngeneic mouse model during 14 days of indicated treatments. * indicates $p<0.038$ by one-way ANOVA with multiple comparisons and Holm-Šídák's *post-hoc* test, Mice/tumors n are placebo=4/8, EPZ6438=5/9, anti-PD1=6/8, combo=5/9, mean +/- upper bound s.e.m. is plotted. **B)** Pie charts depicting cell type proportions identified through the HALO nuclear phenotyping. Mice/tumors n are placebo/untreated=5/10,

EPZ6438=5/10, anti-PD1=5/9, combo=3/6 for syngeneic grafts, and placebo/untreated=5/18, EPZ6438=5/10, anti-PD1=3/8, combo=5/12 for autochthonous lung tumors. * indicates $p=0.042$, ** $p=0.0025$ between placebo and combo by two-tailed t-test. **C)** Percentage of cells staining for CD8 by immunohistochemistry on the same samples shown in (A). **D)** Representative flow cytometry plots with the indicated samples and markers. The left plots are gated on FSC/SSC and Live/Dead negative cells. Spleen samples are shown as staining controls. **E)** Flow cytometry analysis of dissociated tumors from the syngeneic grafts from the indicated treatment arms at day 14. Percentage of CD45+ cells expressing F4/80 or Ly6G are graphed, mean +/- s.e.m. is plotted, placebo n=8, EZH2 inhibitor n=8, anti-PD1 n=9, combo n=7, * indicates $p<0.012$, by one-way ANOVA with multiple comparisons and Holm-Šídák's *post-hoc* test. **F)** Representative images of mouse 1 tumoroids injected into NSG mice after 15 weeks, M indicates male and F indicated female recipients. Both 5x and 10x magnification are show, as well as a tumor from a parental strain mouse injected the same day with the same cell preparation.
