# Supplementary material for: EZH2 Inhibition Promotes Tumor Immunogenicity in Lung Squamous Cell Carcinomas
Source: Cancer Res Commun. 2024 Feb 13;4(2):388–403. doi: 10.1158/2767-9764.CRC-23-0399 (PMC10863487; doi:10.1158/2767-9764.CRC-23-0399)
Supplement: Supplementary Table 6 — shows gene highly expressed in each of the 4 neutrophil clusters called in the single cell RNA-sequencing from murine lung and lung squamous cell carcinoma samples. [file crc-23-0399-s11.pdf]

Supplemental Table 6: Genes Highly Expressed in Neutrophil Clusters, Related to Figure 6
Log2FC=Log2-fold change between cluster and all others, pct=percentage of cells expressing

Cluster	Gene Symbol	Log2FC	pct.1	pct.2	P value	Adj P value	Cluster	Gene Symbol	Log2FC	pct.1	pct.2	P value	Adj P value
Neu-1	Cst3	2.050	0.929	0.784	0.0E+00	0.0E+00	Neu-3	Gadd45b	2.421	0.877	0.664	0.0E+00	0.0E+00
	Gngt2	1.920	0.812	0.557	0.0E+00	0.0E+00		Nceh1	2.367	0.856	0.760	0.0E+00	0.0E+00
	Hexb	1.393	0.794	0.754	0.0E+00	0.0E+00		Gpnmb	2.274	0.781	0.565	0.0E+00	0.0E+00
	Gpx1	1.277	0.869	0.823	0.0E+00	0.0E+00		Psap	2.244	0.852	0.718	0.0E+00	0.0E+00
	Ptgs1	1.126	0.772	0.702	0.0E+00	0.0E+00		Ifrd1	2.039	0.819	0.707	0.0E+00	0.0E+00
	Ms4a6d	0.881	0.805	0.780	0.0E+00	0.0E+00		Hilpda	2.038	0.836	0.799	0.0E+00	0.0E+00
	Gm19951	0.635	0.840	0.837	0.0E+00	0.0E+00		Ccl3	1.982	0.920	0.759	0.0E+00	0.0E+00
	Cenpx	0.430	0.745	0.593	0.0E+00	0.0E+00		Atp6v1c1	1.914	0.857	0.766	0.0E+00	0.0E+00
	Hist1h4d	0.351	0.793	0.747	0.0E+00	0.0E+00		Lamp1	1.832	0.885	0.784	0.0E+00	0.0E+00
	Ranbp1	0.333	0.704	0.546	0.0E+00	0.0E+00		Hcar2	1.829	0.884	0.824	0.0E+00	0.0E+00
	Card11	0.298	0.831	0.810	0.0E+00	0.0E+00		Ctsb	1.789	0.977	0.916	0.0E+00	0.0E+00
	Hist1h1d	0.297	0.697	0.506	0.0E+00	0.0E+00		Cd63	1.786	0.936	0.864	0.0E+00	0.0E+00
	Dmkn	0.263	0.794	0.646	0.0E+00	0.0E+00		Zeb2	1.742	0.829	0.553	0.0E+00	0.0E+00
	Cpt1a	0.256	0.724	0.482	0.0E+00	0.0E+00		Ctsz	1.678	0.936	0.850	0.0E+00	0.0E+00
	H1f0	0.809	0.700	0.554	4.0E-279	5.9E-276		Ftl1	1.590	1.000	0.999	0.0E+00	0.0E+00
	Laptm5	0.807	0.829	0.784	8.0E-255	1.2E-251		F10	1.513	0.863	0.864	0.0E+00	0.0E+00
	Asah1	0.427	0.666	0.444	2.5E-208	3.7E-205		Gas2l3	1.194	0.818	0.737	0.0E+00	0.0E+00
	Atp1a1	1.115	0.779	0.714	3.2E-204	4.8E-201		Dock10	1.193	0.783	0.789	0.0E+00	0.0E+00
	Pmaip1	0.631	0.787	0.697	2.3E-202	3.5E-199		Lhfpl2	1.059	0.784	0.606	0.0E+00	0.0E+00
	Hist1h1e	0.525	0.794	0.723	2.0E-201	2.9E-198		P2rx7	0.743	0.760	0.628	0.0E+00	0.0E+00
	Atp5g1	0.535	0.667	0.503	3.9E-197	5.8E-194		Pdxk	0.707	0.726	0.490	0.0E+00	0.0E+00
	Ltc4s	0.855	0.644	0.562	2.4E-193	3.5E-190		Gstm1	0.948	0.697	0.537	5.0E-285	7.5E-282
	Ptma	1.199	0.760	0.667	4.4E-184	6.5E-181		Tst	0.523	0.688	0.501	9.7E-282	1.5E-278
	Reep5	0.947	0.749	0.636	1.4E-175	2.1E-172		Hexa	1.286	0.839	0.735	4.4E-268	6.5E-265
	Fam96a	0.381	0.589	0.440	2.4E-164	3.7E-161		Atp6v0d2	0.978	0.752	0.684	3.5E-267	5.3E-264
	Cd81	0.475	0.278	0.442	2.2E-155	3.3E-152		Dhfr	0.859	0.789	0.859	3.2E-265	4.7E-262
	Rps2	0.817	0.873	0.819	8.0E-152	1.2E-148		Chka	0.492	0.738	0.529	2.2E-261	3.2E-258
	Chil3	1.278	0.842	0.760	4.3E-130	6.4E-127		Plcxd2	0.704	0.728	0.788	1.7E-250	2.5E-247
	Agap1	0.711	0.586	0.439	4.9E-117	7.3E-114		Fcgr2b	0.662	0.838	0.714	5.9E-241	8.8E-238
	Cd302	0.256	0.296	0.474	2.5E-116	3.8E-113		Gns	1.455	0.782	0.705	7.0E-240	1.1E-236
	Cd300c2	0.778	0.716	0.616	2.5E-111	3.8E-108		Ctsd	0.702	0.987	0.976	1.2E-220	1.8E-217
	Lrp1	0.630	0.529	0.374	2.7E-108	4.0E-105		Atf3	1.518	0.679	0.486	1.4E-214	2.2E-211
	Krt19	0.266	0.659	0.814	1.1E-103	1.7E-100		Npc1	1.184	0.749	0.753	6.1E-211	9.1E-208
	Hebp1	0.441	0.646	0.524	5.1E-103	7.6E-100		Aprt	1.030	0.820	0.695	1.5E-210	2.3E-207
	Ccnd2	0.297	0.487	0.674	3.5E-88	5.2E-85		Cd68	1.480	0.714	0.531	1.7E-208	2.6E-205
	Ssbp4	0.336	0.596	0.543	6.5E-87	9.8E-84		Canx	0.895	0.769	0.640	1.5E-207	2.3E-204
	Tcf4	0.262	0.442	0.288	1.4E-86	2.1E-83		Cd274	1.443	0.725	0.671	2.9E-205	4.3E-202
	Dpep2	0.452	0.652	0.764	1.9E-86	2.8E-83		Plekhm2	1.059	0.785	0.766	4.3E-197	6.4E-194
	Grn	0.508	0.869	0.830	2.0E-84	2.9E-81		Prdx1	1.174	0.673	0.462	6.6E-191	9.9E-188
	Calr	0.613	0.710	0.613	1.3E-82	1.9E-79		Tpp1	1.030	0.716	0.620	1.2E-185	1.8E-182
	B930036N10Ri	0.429	0.642	0.682	1.8E-82	2.7E-79		Naglu	0.956	0.616	0.469	3.1E-165	4.6E-162
	Naaa	0.466	0.655	0.600	1.6E-81	2.4E-78		H2-Eb1	0.929	0.683	0.729	7.5E-150	1.1E-146
	Pgls	0.322	0.607	0.512	2.7E-81	4.0E-78		Dhrs3	0.764	0.674	0.525	6.6E-145	9.9E-142
	Id2	0.400	0.925	0.882	1.7E-75	2.6E-72		Dnmt1	0.341	0.626	0.526	3.8E-137	5.7E-134
	Bcl2a1b	0.796	0.640	0.563	8.2E-75	1.2E-71		Npc2	1.027	0.875	0.886	1.8E-132	2.7E-129
	Ctsa	0.531	0.744	0.686	2.7E-68	4.0E-65		Tmem86a	0.556	0.724	0.748	2.4E-128	3.5E-125
	Manf	0.354	0.814	0.794	6.8E-68	1.0E-64		Gadd45g	1.393	0.634	0.509	5.6E-122	8.4E-119
	Cybb	0.303	0.519	0.409	4.6E-65	6.8E-62		Id2	0.969	0.874	0.904	4.8E-121	7.2E-118
	Gm26917	0.282	0.585	0.474	1.5E-61	2.2E-58		Hpgds	0.522	0.680	0.724	9.2E-119	1.4E-115
	Ndufa4	0.667	0.623	0.564	1.9E-60	2.9E-57		Inhba	0.874	0.622	0.511	6.6E-114	9.9E-111
	Hist1h1c	0.752	0.664	0.572	3.1E-58	4.7E-55		Tcirg1	1.016	0.670	0.594	1.6E-112	2.5E-109
	Bcl2a1a	0.768	0.592	0.516	3.8E-58	5.7E-55		Lgmn	0.951	0.627	0.518	1.4E-111	2.1E-108
	Gm2a	0.892	0.637	0.616	1.3E-56	2.0E-53		Ccnf	0.540	0.662	0.781	5.7E-109	8.6E-106
	Phlda1	0.268	0.391	0.530	1.3E-55	1.9E-52		Creg1	1.377	0.702	0.577	1.2E-106	1.9E-103
	Mt1	0.286	0.750	0.734	4.5E-55	6.8E-52		Myo5a	0.533	0.480	0.319	2.3E-99	3.5E-96
	Hist1h4i	0.880	0.646	0.592	5.1E-54	7.6E-51		Mpeg1	0.803	0.701	0.585	5.7E-99	8.5E-96

	Nap1l1	0.311	0.514	0.434	7.4E-52	1.1E-48
	Ccng1	0.519	0.520	0.461	1.5E-51	2.2E-48
	Unc93b1	0.566	0.570	0.493	1.8E-51	2.6E-48
	Pycard	0.298	0.870	0.846	2.1E-51	3.1E-48
	P2ry6	0.379	0.710	0.721	1.9E-50	2.9E-47
	Tubb5	0.364	0.356	0.505	2.0E-50	3.0E-47
	Tubb4b	0.403	0.662	0.575	5.7E-48	8.6E-45
	Pdia6	0.555	0.775	0.771	7.2E-48	1.1E-44
	Gm20186	0.390	0.404	0.535	4.5E-47	6.7E-44
	Ucp2	0.715	0.743	0.739	8.8E-42	1.3E-38
	Clec12a	0.455	0.481	0.424	1.0E-39	1.6E-36
	Colgalt1	0.261	0.486	0.423	4.1E-39	6.1E-36
	Cks2	0.955	0.534	0.469	1.2E-36	1.8E-33
	Rpn1	0.256	0.347	0.457	3.6E-31	5.3E-28
	Id1	0.318	0.832	0.819	1.9E-28	2.8E-25
	Fam20c	0.332	0.792	0.904	6.0E-27	9.0E-24
	Ctsc	0.283	0.661	0.629	8.2E-26	1.2E-22
	Lamtor4	0.498	0.641	0.610	1.3E-25	2.0E-22
	Csf1r	0.363	0.280	0.375	2.1E-25	3.2E-22
	Rhoc	0.353	0.529	0.648	1.6E-24	2.4E-21
	Cdc42ep3	0.331	0.512	0.462	5.0E-23	7.5E-20
	Krt18	0.259	0.611	0.616	1.1E-21	1.7E-18
	Rgs1	0.444	0.650	0.843	6.1E-21	9.2E-18
	H2afz	0.536	0.744	0.728	1.6E-19	2.5E-16
	Rpl3	0.306	0.538	0.517	1.6E-16	2.4E-13
	Hist1h2ap	0.291	0.508	0.492	1.7E-16	2.5E-13
	Mpeg1	0.501	0.595	0.621	2.6E-16	3.8E-13
	Cfp	0.260	0.543	0.504	1.5E-15	2.2E-12
	Tubb6	0.260	0.394	0.341	4.6E-14	7.0E-11
	Erp29	0.740	0.600	0.627	1.3E-13	2.0E-10
	Fyb	0.264	0.751	0.780	2.7E-10	4.0E-07
	Spp1	0.341	0.831	0.843	3.7E-09	5.5E-06
	Atad2	0.270	0.495	0.481	4.1E-09	6.2E-06
	Bcl2a1d	0.651	0.380	0.413	7.1E-08	1.1E-04
	S100a10	0.397	0.513	0.610	3.7E-07	5.6E-04
	Ctss	0.529	0.679	0.716	6.8E-07	1.0E-03
	Apoe	0.328	0.748	0.737	8.0E-06	1.2E-02
	Tnf	0.345	0.327	0.311	8.5E-06	1.3E-02
	Gm12840	1.062	0.321	0.302	6.5E-05	9.7E-02
	Pou2f2	0.584	0.387	0.411	7.3E-04	1.0E+00
	Aprt	0.360	0.639	0.770	1.4E-03	1.0E+00
	Abca1	0.524	0.768	0.829	4.0E-03	1.0E+00
	Tgfbi	0.309	0.586	0.654	5.9E-03	1.0E+00
	Acp5	0.644	0.451	0.471	7.0E-03	1.0E+00
**Neu-2**	BC100530	1.757	0.815	0.739	0.0E+00	0.0E+00
	Gm5483	1.712	0.938	0.789	0.0E+00	0.0E+00
	Wfdc17	1.379	0.993	0.891	0.0E+00	0.0E+00
	Cxcl2	1.173	0.951	0.703	0.0E+00	0.0E+00
	Ifitm1	1.111	0.963	0.776	0.0E+00	0.0E+00
	Lrg1	0.856	0.929	0.751	0.0E+00	0.0E+00
	Egr1	0.840	0.912	0.633	0.0E+00	0.0E+00
	Retnlg	0.817	0.960	0.812	0.0E+00	0.0E+00
	Wfdc21	0.764	0.945	0.788	0.0E+00	0.0E+00
	Ccl6	0.733	0.910	0.693	0.0E+00	0.0E+00
	Lcn2	0.710	0.911	0.736	0.0E+00	0.0E+00
	Cxcl3	0.666	0.840	0.353	0.0E+00	0.0E+00
	Tceal9	0.526	0.888	0.729	0.0E+00	0.0E+00
	Slfn4	0.469	0.942	0.649	0.0E+00	0.0E+00
	Id1	0.277	0.915	0.784	4.8E-284	7.2E-281
	Slpi	0.579	0.904	0.662	4.9E-284	7.3E-281
	Dgat2	0.449	0.795	0.605	5.1E-282	7.6E-279
	Adam8	1.014	0.729	0.463	8.4E-262	1.3E-258
	Prok2	0.277	0.790	0.700	4.3E-239	6.4E-236
	Vim	0.636	0.831	0.556	1.3E-219	2.0E-216
	G0s2	0.703	0.819	0.568	3.6E-170	5.3E-167
	Glrx	0.709	0.678	0.390	5.3E-170	7.9E-167
	Ccl4	1.163	0.868	0.935	3.7E-95	5.6E-92
	Syngr1	0.793	0.721	0.791	7.6E-92	1.1E-88
	Aplp2	0.334	0.718	0.588	7.3E-91	1.1E-87
	Hmox1	1.205	0.790	0.778	9.1E-85	1.4E-81
	Hspa9	0.589	0.651	0.663	2.2E-81	3.4E-78
	Hspa5	0.742	0.800	0.740	2.0E-80	2.9E-77
	Dpp7	0.502	0.624	0.692	1.0E-72	1.5E-69
	Hsp90b1	0.441	0.736	0.690	3.7E-72	5.5E-69
	Rgs1	0.942	0.774	0.774	7.5E-70	1.1E-66
	Hk2	0.381	0.466	0.660	4.3E-69	6.4E-66
	Sqstm1	0.921	0.608	0.494	1.2E-67	1.8E-64
	Emp1	0.827	0.723	0.773	6.3E-65	9.4E-62
	Rps2	0.628	0.867	0.830	3.3E-63	4.9E-60
	Hspa1b	0.866	0.659	0.604	4.8E-63	7.2E-60
	Fam20c	0.379	0.847	0.869	2.1E-60	3.1E-57
	Slc37a2	0.582	0.609	0.576	3.5E-55	5.3E-52
	Ctsl	0.651	0.807	0.742	5.6E-54	8.4E-51
	Fabp5	0.488	0.626	0.784	3.1E-49	4.6E-46
	Ctsa	0.516	0.748	0.695	6.2E-49	9.3E-46
	Hsp90aa1	1.357	0.762	0.791	1.2E-48	1.8E-45
	Timp2	1.159	0.724	0.658	9.5E-47	1.4E-43
	Hal	0.601	0.514	0.776	7.5E-29	1.1E-25
	Cldn1	0.594	0.582	0.669	4.4E-28	6.6E-25
	Jdp2	0.437	0.670	0.653	2.7E-25	4.0E-22
	Acod1	0.845	0.548	0.530	8.1E-24	1.2E-20
	Slc7a11	0.925	0.565	0.555	1.8E-23	2.7E-20
	Grn	0.382	0.873	0.835	1.8E-21	2.8E-18
	Vegfa	0.831	0.495	0.452	1.0E-20	1.6E-17
	Gm26870	0.478	0.567	0.719	2.0E-19	3.0E-16
	Gusb	0.335	0.618	0.634	2.3E-19	3.5E-16
	Cd300c2	0.375	0.675	0.645	4.3E-19	6.5E-16
	Hspe1	0.432	0.639	0.663	1.4E-15	2.1E-12
	Slc43a3	0.361	0.577	0.707	1.5E-15	2.3E-12
	Abcg1	0.435	0.571	0.559	2.1E-15	3.2E-12
	Syne1	0.692	0.511	0.667	2.2E-14	3.3E-11
	Rps6ka2	0.368	0.521	0.558	4.6E-14	6.8E-11
	Ftl1-ps1	0.479	0.338	0.347	1.7E-11	2.5E-08
	C3	0.346	0.632	0.634	4.3E-11	6.5E-08
	Thbs1	1.191	0.451	0.593	1.1E-09	1.6E-06
	Hbb-bs	0.414	0.804	0.812	3.3E-09	5.0E-06
	Rpl3	0.505	0.532	0.522	5.9E-09	8.8E-06
	Acp5	0.601	0.473	0.461	1.5E-03	1.0E+00
	Hspa1a	0.817	0.527	0.564	1.7E-03	1.0E+00
	Spp1	1.001	0.762	0.862	5.5E-03	1.0E+00
**Neu-5-IFN**	Isg15	3.959	0.914	0.574	8.6E-166	1.3E-162
	Rsad2	3.922	0.893	0.799	5.5E-153	8.3E-150
	Ifi47	2.686	0.858	0.765	3.1E-121	4.7E-118
	Ifitm3	2.227	0.937	0.742	1.4E-114	2.1E-111
	Gbp2	3.371	0.883	0.701	2.0E-112	3.0E-109
	Ifit2	1.589	0.827	0.776	2.5E-109	3.8E-106
	Rtp4	2.640	0.820	0.711	1.3E-94	1.9E-91
	Slfn4	2.454	0.848	0.736	8.9E-90	1.3E-86
	Slfn5	2.495	0.787	0.619	1.1E-87	1.7E-84
	Ifit1bl2	0.982	0.794	0.613	2.2E-77	3.3E-74
	Isg20	2.415	0.731	0.518	3.4E-64	5.1E-61
	Ifit1	3.118	0.693	0.425	1.5E-61	2.2E-58
	Zbp1	1.428	0.789	0.729	2.5E-60	3.7E-57
	Ifi202b	0.654	0.739	0.617	3.0E-57	4.5E-54
	Ifit3b	2.094	0.698	0.535	7.2E-57	1.1E-53
	Oasl1	2.280	0.668	0.414	6.9E-56	1.0E-52
	Ifit3	3.099	0.612	0.263	4.7E-39	7.0E-36
	Cldn1	0.258	0.777	0.646	1.1E-38	1.6E-35
	Bst2	1.698	0.657	0.525	6.1E-38	9.1E-35
	Ctss	1.414	0.764	0.701	7.1E-37	1.1E-33
	Fcgr4	0.876	0.812	0.699	1.1E-32	1.6E-29
	Cmpk2	1.332	0.647	0.692	9.6E-30	1.4E-26

	F630028O10Ri	0.557	0.798	0.542	6.6E-167	9.8E-164
	Ier3	0.594	0.972	0.826	5.5E-157	8.2E-154
	Steap4	0.560	0.732	0.674	8.3E-127	1.2E-123
	Gadd45a	0.551	0.731	0.616	2.4E-122	3.6E-119
	Stfa2l1	1.367	0.682	0.553	2.0E-118	3.0E-115
	Csf2rb	0.259	0.921	0.714	1.3E-114	1.9E-111
	Stfa2	0.989	0.680	0.704	2.8E-87	4.2E-84
	Il1f9	0.250	0.718	0.540	2.8E-75	4.3E-72
	Asprv1	0.555	0.707	0.722	3.9E-33	5.8E-30
	Saa3	0.387	0.748	0.691	3.2E-27	4.7E-24
	Tacstd2	0.305	0.584	0.468	3.7E-24	5.5E-21
	Osm	0.506	0.460	0.331	1.0E-09	1.6E-06
	Hacd4	0.294	0.496	0.406	4.6E-03	1.0E+00
**Neu-4-classic**	Retnlg	3.329	0.993	0.845	0.0E+00	0.0E+00
	Ifitm6	3.306	0.992	0.478	0.0E+00	0.0E+00
	Lcn2	2.827	0.999	0.771	0.0E+00	0.0E+00
	Wfdc21	2.632	0.999	0.822	0.0E+00	0.0E+00
	Mmp8	2.622	0.971	0.633	0.0E+00	0.0E+00
	Ly6g	2.187	0.944	0.567	0.0E+00	0.0E+00
	Anxa1	2.167	0.997	0.716	0.0E+00	0.0E+00
	Prok2	2.127	0.960	0.707	0.0E+00	0.0E+00
	Lrg1	1.793	0.992	0.789	0.0E+00	0.0E+00
	Ifitm3	1.588	0.989	0.726	0.0E+00	0.0E+00
	Cd177	1.349	0.907	0.656	0.0E+00	0.0E+00
	Ggt1	1.045	0.897	0.603	0.0E+00	0.0E+00
	Glul	0.436	0.853	0.387	0.0E+00	0.0E+00
	Gyg	0.996	0.863	0.596	8.3E-285	1.2E-281
	Wfdc17	1.832	0.997	0.916	2.1E-279	3.1E-276
	Chil1	0.973	0.942	0.592	8.9E-269	1.3E-265
	Steap4	0.716	0.940	0.670	1.5E-254	2.2E-251
	Stfa2	1.185	0.909	0.678	2.8E-254	4.2E-251
	Tgm1	0.493	0.865	0.635	2.0E-252	3.0E-249
	Mgst1	0.981	0.870	0.580	2.4E-243	3.6E-240
	Flna	0.955	0.875	0.433	4.9E-242	7.4E-239
	Ngp	1.645	0.786	0.355	7.6E-234	1.1E-230
	Slpi	1.162	0.978	0.715	2.2E-232	3.3E-229
	Hacd4	1.004	0.822	0.400	5.4E-230	8.1E-227
	Ifitm1	1.769	0.992	0.819	1.3E-203	2.0E-200
	Ccl6	1.106	0.959	0.742	2.3E-188	3.5E-185
	Smpdl3a	0.852	0.826	0.558	3.0E-187	4.5E-184
	Tuba1a	0.578	0.780	0.474	1.3E-175	1.9E-172
	Vim	0.919	0.966	0.611	2.8E-175	4.1E-172
	Syne1	0.469	0.810	0.615	7.0E-163	1.1E-159
	Tgfbi	0.673	0.922	0.604	6.7E-159	1.0E-155
	Glrx	0.596	0.853	0.445	1.3E-157	2.0E-154
	Tacstd2	0.565	0.812	0.476	1.1E-141	1.7E-138
	Pi16	0.611	0.785	0.543	5.7E-120	8.6E-117
	Lyz2	0.426	0.978	0.755	2.3E-116	3.4E-113
	Lmo4	0.573	0.715	0.315	5.2E-112	7.9E-109
	Ly6c2	0.617	0.708	0.439	1.4E-109	2.1E-106
	BC100530	1.364	0.877	0.752	3.0E-94	4.5E-91
	Aldh2	0.324	0.698	0.433	2.9E-86	4.3E-83
	Stfa2l1	0.440	0.835	0.571	5.3E-68	7.9E-65
	Ceacam1	0.277	0.709	0.530	2.0E-64	3.0E-61
	Plac8	0.327	0.668	0.476	5.6E-64	8.4E-61
	Acvrl1	0.598	0.673	0.666	1.5E-62	2.3E-59
	Camp	1.496	0.597	0.402	1.9E-49	2.9E-46
	Serpinb1a	0.774	0.616	0.519	1.8E-45	2.7E-42
	Abcd2	0.289	0.621	0.535	1.2E-40	1.8E-37
	Stfa3	0.395	0.382	0.646	1.5E-34	2.3E-31
	Sept9	0.260	0.546	0.375	1.8E-28	2.7E-25
	Olfm4	0.922	0.683	0.609	5.7E-22	8.6E-19
	Asprv1	0.255	0.706	0.718	2.2E-13	3.3E-10
	C130026I21Rik	0.253	0.399	0.417	2.3E-11	3.5E-08

	Clec4a3	0.377	0.614	0.450	1.1E-28	1.6E-25
	Acod1	0.910	0.764	0.528	9.0E-26	1.3E-22
	Il18	0.463	0.556	0.366	4.8E-23	7.2E-20
	Fxyd3	0.255	0.571	0.443	7.7E-23	1.2E-19
	Irf7	1.505	0.642	0.564	5.3E-21	7.9E-18
	Il18bp	0.433	0.508	0.318	7.1E-20	1.1E-16
	Cxcl10	1.827	0.551	0.388	8.9E-20	1.3E-16
	Lair1	0.305	0.609	0.516	8.0E-19	1.2E-15
	Gm4316	0.443	0.279	0.343	1.0E-17	1.5E-14
	Npc2	0.571	0.904	0.883	1.2E-17	1.9E-14
	Gbp5	1.547	0.660	0.801	1.3E-17	1.9E-14
	AW112010	0.469	0.520	0.370	1.3E-17	1.9E-14
	Cd274	0.977	0.736	0.682	3.9E-16	5.9E-13
	Hes1	0.375	0.358	0.549	4.7E-16	7.0E-13
	Ifi207	0.258	0.655	0.691	6.5E-15	9.7E-12
	Usp18	1.418	0.604	0.700	7.7E-15	1.2E-11
	Abcg1	0.446	0.701	0.558	1.4E-13	2.2E-10
	Fyb	0.553	0.835	0.768	1.2E-12	1.8E-09
	Bcl2a1b	0.420	0.751	0.586	9.3E-11	1.4E-07
	Cxcl9	0.426	0.736	0.737	1.9E-08	2.8E-05
	Ifi27l2a	2.036	0.553	0.506	1.4E-07	2.1E-04
	Cybb	0.284	0.546	0.446	3.7E-07	5.5E-04
	Ly6i	0.647	0.586	0.648	9.8E-07	1.5E-03
	Plac8	1.572	0.533	0.491	1.0E-06	1.6E-03
	Mpeg1	0.339	0.726	0.608	2.2E-06	3.4E-03
	Emp3	0.292	0.447	0.314	2.4E-06	3.5E-03
	Gm20234	0.310	0.609	0.679	2.7E-06	4.1E-03
	Cst3	0.308	0.858	0.835	9.4E-06	1.4E-02
	Ddx60	1.381	0.528	0.576	2.6E-05	3.9E-02
	Ifi209	1.094	0.563	0.675	6.0E-05	9.0E-02
	Unc93b1	0.390	0.569	0.519	6.2E-05	9.3E-02
	Hba-a1	0.963	0.429	0.553	1.2E-04	1.7E-01
	Lyz2	0.310	0.838	0.771	1.9E-03	1.0E+00
	Ccl4	0.628	0.921	0.920	2.1E-03	1.0E+00
	Ly6c2	1.048	0.482	0.460	3.0E-03	1.0E+00
