# Supplementary material for: EZH2 Inhibition Promotes Tumor Immunogenicity in Lung Squamous Cell Carcinomas
Source: Cancer Res Commun. 2024 Feb 13;4(2):388–403. doi: 10.1158/2767-9764.CRC-23-0399 (PMC10863487; doi:10.1158/2767-9764.CRC-23-0399)
Supplement: Supplementary Figure 6 — shows analysis of bone marrow from mice treated with EZH2 inhibition and anti-PD1. [file crc-23-0399-s12.pdf]

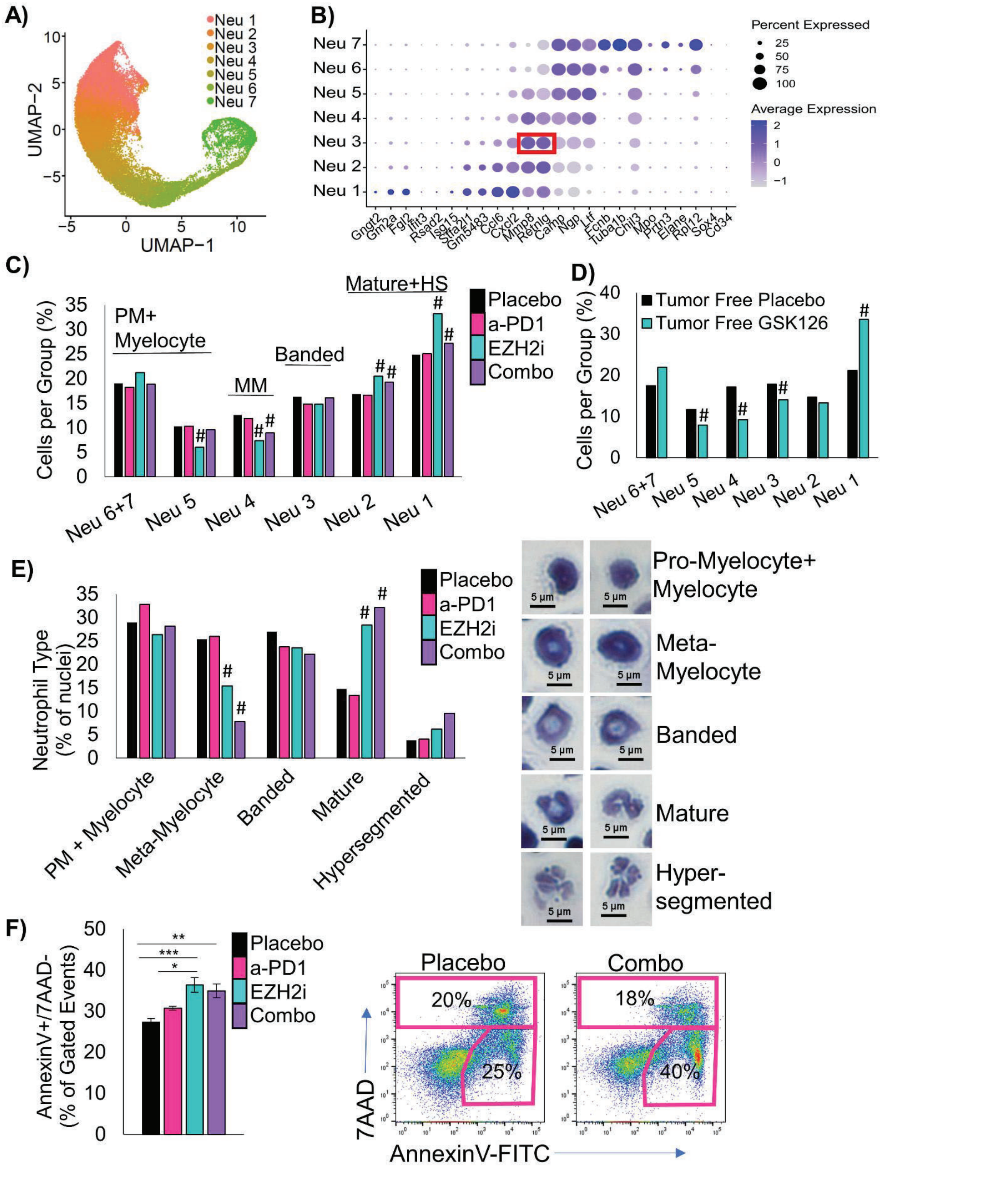

Supplementary Figure 6: Related to Figure 6

A) Annotated Uniform Manifold Approximation and Projection (UMAP) plot showing the 7 different populations of neutrophils within the bone marrow of tumor-bearing mice treated with placebo, GSK126, anti-PD1, or combined GSK126 with anti-PD1. **B)** Dot plot showing the relative expression of marker genes (x-axis) in each

neutrophil cluster (y-axis). Expression of *Mmp8* and *Rentlg* were shown to be enriched in banded neutrophils. **C)** Percentage of cells per treatment group in tumor-bearing mice graphed for the neutrophil populations, # indicates adjusted p<0.008 by proportion z-test. **D)** Percentage of cells per treatment group in tumor free mice graphed for the neutrophil populations, # indicates adjusted p<0.008 by proportion z-test. **E)** Proportions of different nuclear morphologies in bone marrow cytospins, average of n=2 samples for Placebo, n=1 sample for others, 500 nuclei were counted, # indicates p<0.0004 with Fisher's Exact test between Vehicle and EZH2 inhibitor or Vehicle and Combo for meta-myelocyte vs mature neutrophils. Representative images of nuclei types shown, scale bar = 5µm. **F)** Percentage of AnnexinV+/7AAD- cells in bone marrow cultures 48 hours post isolation from mice treated with the indicated therapies, n=3 biological replicates each with 2 experimental replicates for placebo, EPZ6438 and combo, n=4 biological replicates each with 2 experimental replicates for anti-PD1, * indicates p=0.012, **p=0.0017, ***p= 0.0003 by one-way ANOVA with pairwise comparisons and Holm-Šídák's *post-hoc* test. Representative flow plots of placebo and combination treated cultures shown.
